# Hydrogenation of CO_2_ Promoted by Silicon-Activated H_2_S: Origin and Implications

**DOI:** 10.3390/molecules26010050

**Published:** 2020-12-24

**Authors:** Xing Liu

**Affiliations:** College of Chemistry and Chemical Engineering, Southwest University, Chongqing 400715, China; xingliu1986@swu.edu.cn

**Keywords:** CO_2_ activation, quantum chemical calculation, reaction mechanisms, chemical bond

## Abstract

Unlike the usual method of CO*_x_* (*x* = 1, 2) hydrogenation using H_2_ directly, H_2_S and HSiSH (silicon-activated H_2_S) were selected as alternative hydrogen sources in this study for the CO*_x_* hydrogenation reactions. Our results suggest that it is kinetically infeasible for hydrogen in the form of H_2_S to transfer to CO*_x_* at low temperatures. However, when HSiSH is employed instead, the title reaction can be achieved. For this approach, the activation of CO_2_ is initiated by its interaction with the HSiSH molecule, a reactive species with both a hydridic H^δ−^ and protonic H^δ+^. These active hydrogens are responsible for the successive C-end and O-end activations of CO_2_ and hence the final product (HCOOH). This finding represents a good example of an indirect hydrogen source used in CO_2_ hydrogenation through reactivity tuned by silicon incorporation, and thus the underlying mechanism will be valuable for the design of similar reactions.

## 1. Introduction

In the past two decades, global warming caused by excessive CO_2_ emission has received much attention [1,2]. An effective way to alleviate this environmental problem is to reduce carbon dioxide to value-added chemicals through hydrogen [3,4]. In addition to hydrogen, some indirect hydrogen sources such as silanes have also been used to achieve this goal [5]. Can hydrogen sulfide (H_2_S), as an indirect hydrogen source, work as well? If so, it will be very important from both environmental and public safety perspectives since H_2_S is known as one of the most toxic, corrosive, and malodorous gases in various industrial processes [6,7]. Research in this field is still in its infancy compared with the area of CO*_x_* hydrogenation reactions that use H_2_ directly [8,9,10]. Nevertheless, I note that H_2_S has previously been modeled to act as a source of hydrogen atoms in the first excited state, and two pathways leading to HCOO + SH and HCOOH + S have been revealed [11]. However, UV radiation is required to trigger these reactions, which is hardly cost effective. Experimentally, although the reduction of CO_2_ with H_2_S in a simulated deep sea hydrothermal vent system has been observed [12], it is not H_2_S but H_2_O that acts as a hydrogen donor in HCO_3_^-^ hydrogenation to formate. Different roles of H_2_S during CO_2_ hydrogenation on the MoS_2_ catalyst have also been probed [13]. It was observed that under high co-feeds conditions, H_2_S could react with CO_2_ to form COS. The same product has also been reported for the H_2_S + CO_2_ reaction under a metal free dual organocatalyst [14]. The emerging role of COS deserves attention. In the presence of H_2_, this intermediate competes well with CO_2_ or CO (in H_2_S + CO reaction) [15,16,17] in the hydrogenation step to produce CH_3_SH [18] and CH_3_SCH_3_ [19] molecules. Clearly, if active hydrogens (from H_2_S) are available for CO*_x_* hydrogenation, the formation of COS intermediate should be minimized. So, how to ensure this when H_2_S is employed as a potential hydrogen source? In a recent matrix isolation study [20], it was found that Si atoms can react spontaneously with H_2_S to produce a HSiSH molecule that is stable until exposure to UV radiation. If the two hydrogens (from HSiSH) are active toward CO*_x_* hydrogenation, SiS, a very stable molecule with Si=S multiple bonds [21,22,23], will be one of the final products, which suggests little chance for the production of COS, and hence contributes to the target reaction.

In this study, with the aid of quantum chemical calculations, the reactions of H_2_S and HSiSH with CO*_x_* were investigated. I found that silicon incorporation increases the dehydrogenation of H_2_S while passivating the sulfur atom, which increases the possibility of CO_2_ hydrogenation. Furthermore, the reaction mechanisms, especially the structures and interconversion of some important reactive intermediates in the relevant reactions, were explored in detail, thus offering new ideas for the design of similar reactions involving S-H and C=O bond activation and transformation.

## 2. Results

**Figure 1:**
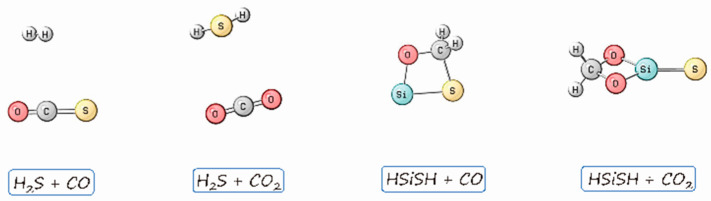
Global minima obtained for the reactions of H_2_S and HSiSH with CO*_x_* (*x* = 1, 2).

**Figure 2:**
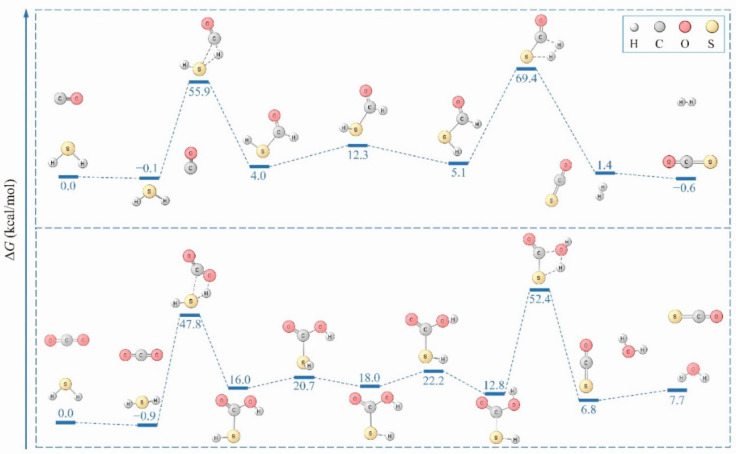
Reaction profiles for H_2_S + CO*_x_* (*x* = 1, 2) (all energetics were refined by the CCSD(T) method).

The global minimum (Figure 1) obtained on the H_2_S + CO potential energy surface (PES) was H_2_ + COS, which was computed to be exothermic by 0.6 kcal·mol^−1^ relative to the entry channel. However, similar calculations confirmed an H_2_S···CO_2_ complex as a global minimum, indicating the inertness of CO_2_ in the hydrogen transfer reaction. Interestingly, when HSiSH was used instead, C-H bonds were formed for the most stable species (Figure 1, right panel), exhibiting prominent hydrogen transfer characteristics. Are these structures kinetically accessible? I first explored the energy landscape to access H_2_ + COS, which suggested a preference for breaking the S-H and C-H bonds for H_2_ production (Figure 2, upper panel). In this pathway, three sequential steps are involved: H_2_S addition to CO to give *trans*-HCOSH; isomerization of this molecule to *cis*-HCOSH; and finally, cleavage of the C-H and S-H bonds to afford H_2_ + COS. The high activation energy predicted for this reaction implies that it is kinetically infeasible for hydrogens in the form of H_2_S to transfer to CO at low temperatures, and hence explains the difficulty in generating the H_2_ + COS species. For completeness, the pathways leading to stable but much less favorable products like CS + H_2_O were also explored, and are summarized in Figure S3 in the Supporting Information (SI).

**Figure 3:**
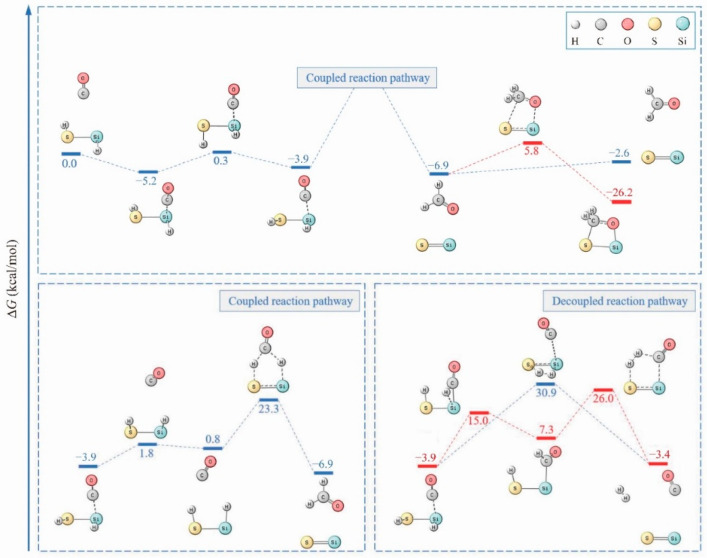
Reaction profiles for HSiSH + CO, with coupled and decoupled reaction pathways illustrated in the lower panel (all energetics were refined by the CCSD(T) method).

Although the H_2_S···CO_2_ complex is the most stable species on the CO_2_ + H_2_S PES, the reaction channels leading to the COS + H_2_O product were explored and are presented in Figure 2 (lower panel) and Figure S6. It is clear that the initial step of hydrogen transfer that occurs during CO_2_ reduction is energy demanding (48.7 kcal·mol^−1^), which is comparable to the value of 49.6 kcal·mol^−1^ reported in a previous study [11]. Additionally, the step for COS···H_2_O production is difficult at low temperatures. With an efficient catalyst [14], these barriers can be reduced significantly. However, the conversion of CO*_x_* to COS makes it less likely that the CO*_x_* hydrogenation reaction will occur. Furthermore, the active hydrogens are lost in the form of H_2_ or H_2_O. Interestingly, when H_2_S is replaced with HSiSH, disparate behaviors are predicted toward CO*_x_* activation. In the case of CO, a global minimum with a four-membered ring can form through concerted double hydrogen transfer from HSiSH to CO (Figure 3). Since the hydrogen transfer involves simultaneous HSiSH dehydrogenation and CO hydrogenation, “coupled reaction pathway” is employed here to denote it. If the hydrogen is released (from HSiSH) instead, “decoupled reaction pathway” is more appropriate. In the coupled reaction pathway, an alternative step to produce the kinetically more stable SiS + HCHO is clear. However, competitive channels for hydrogen release (in the decoupled reaction pathway) are, from an energetic point of view, more effective (Figure 3, lower panel), and CO even acts as a catalyst in this reaction. As can be inferred from the figure, the Gibbs free energy barrier for two-step dehydrogenation is about 18.8 kcal·mol^−1^ on average while that for one-step dehydrogenation amounts to 34.8 kcal·mol^−1^. In other words, the thermodynamically favored hydrogen transfer reaction to reach the global minimum is decoupled or at least partially decoupled for H_2_ production. As for the global minimum for CO_2_, the two pathways toward its formation are presented in the SI (Figure S13), and the rate-limiting step is appreciably higher: at least 57.7 kcal·mol^−1^ at the M06-2X/aug-cc-pVTZ level, revealing the difficulties in its generation.

**Figure 4:**
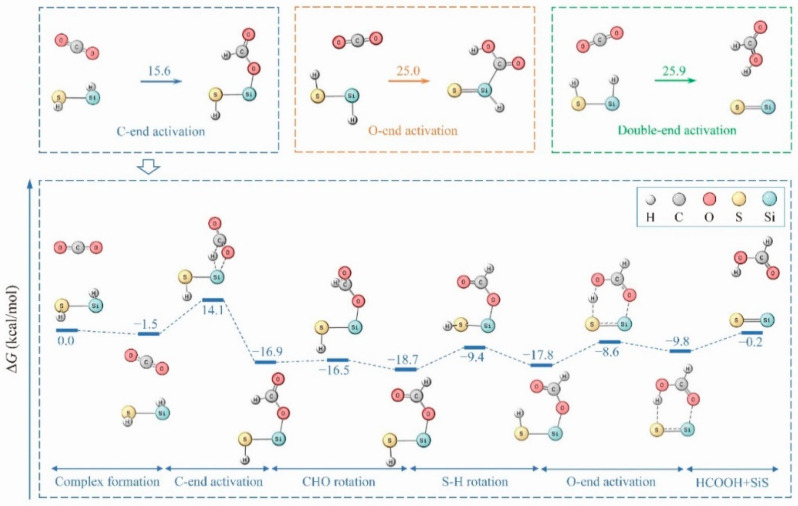
Optimal path obtained for the HSiSH + CO_2_ reaction; the activation energies for different pathways are shown in the upper panel (all energetics were refined by the CCSD(T) method).

Remarkably, HSiSH exhibits a significant reactivity toward CO_2_ in the optimal path (Figure 4). It can easily form a precursor complex with CO_2_ by *ƞ*^1^-O coordination, and a short Si-O bond distance (2.855 Å) is obtained as expected. The subsequent attack by Si-H on the carbon of CO_2_ appears to be key to initiating CO_2_ reduction. In this step, a transition state (TS) containing a near-planar ring as well as a bent CO_2_ is predicted. The contraction of Si-O and the elongation of both Si-H and C=O in this TS structure are consistent with the activation of CO_2_, which then produces an insertion product with a HCO_2_ moiety. It is worth noting that the Gibbs free energy barrier (15.6 kcal·mol^−1^) to reach this TS is the highest among the saddle points, indicating that the hydrosilylation of CO_2_ is the rate-limiting step.

To fulfill the reductive elimination of HCOOH, two successive rotations—one of the OCH moiety around the C-O bond and the other of the thiol group around the Si-S bond—are required. Such structural rearrangements give a reactive intermediate with a short O···H distance (2.120 Å) and small O-C-O-H dihedral angle (21.0°). Clearly, these geometric features are favorable for subsequent O-end activation of the formate ligand, and it is therefore not surprising that the Gibbs free energy barrier to reach the last TS is only 9.2 kcal·mol^−1^. The result is then a *η*^2^-O,H-dihapto complex, as shown in Figure 4, where the oxygen and hydrogen of the formate ligand are bound to the silicon atom and sulfur atom, respectively. Free HCOOH can be liberated from this complex with an energy demand of 9.6 kcal·mol^−1^. Although this optimal path is slightly exothermic (0.2 kcal·mol^−1^), it should be mentioned that this energy does not include the silicon incorporation step. In fact, the insertion of an Si atom into the S-H bond of H_2_S is spontaneous and highly exothermic (66.2 kcal·mol^−1^ at the CCSD(T)/6-311++g(3df,3pd)//B3LYP/6-311++g(3df,3pd) level) as proved in a previous study [20].

The pathways explored in this reaction can simply be classified into three categories according to the initial activation site(s): C-end activation pathways, O-end activation pathways, and double-end (C, O) activation pathways. The optimal pathway associated with C-end activation, as presented above, involves an HCOO intermediate and gives HCOOH as the final product. Figure S12 in the SI illustrates some less favorable products in this category; details for their formation are summarized in Figure S13. As shown in the figure, SSiO + HCHO, SiO_2_ + H_2_CS, or SiO + HCOSH lies even above the TS in the rate-limiting step of the optimal path, suggesting the unavailability of these species. Although SiS + H_2_ + CO_2_ is more stable than SiS + HCOOH by 3.5 kcal·mol^−1^, the predicted rise in the barrier height shows again the preference for HCOOH species. In addition to the C-end activation pathway, HCOOH can, alternatively, form through the double-end activation pathway as shown above. However, the pathway in the category of O-end activation produces predominantly SiS + CO + H_2_O via a COOH intermediate (SI, Figures S14 and S15). There are, indeed, several molecules that one can conceive as products in this category. However, as far as the thermodynamics of the reaction are concerned, SSiO + HCHO, SSiO + CO + H_2,_ and SiO + CO + H_2_S all lie above SiS + CO + H_2_O by 8.3, 7.1, and 4.7 kcal·mol^−1^, respectively. Besides, the channels leading to these species are kinetically less favored (SI, Figure S15). A similar situation is encountered for products HCOOH + SiS and SiS + CO_2_ + H_2_ though they are more stable than SiS + CO + H_2_O by 7.3 and 10.8 kcal·mol^−1^, respectively (SI, Figure S15). Since the activation energies of O-end activation and double-end (C, O) activation pathways are approximately 10.0 kcal·mol^−1^ higher than that of C-end activation pathways, and the interconversion between the HCOO and COOH intermediates encounters a large Gibbs free energy barrier (M06-2X/aug-cc-pVTZ: 103.7 kcal·mol^−1^), the efficient production of formic acid can be expected.

## 3. Discussion

**Figure 5:**
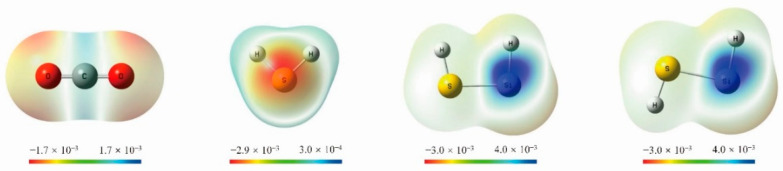
Dual descriptors computed for the H_2_S, CO_2_, and *cis*-/*trans*-HSiSH molecules; nucleophilic and electrophilic zones are represented in red and blue, respectively (isovalue for surfaces: ρ = 0.01 a.u.).

Without the incorporation of Si into H_2_S, the H_2_S···CO_2_ complex is calculated as the global minimum on the PES, and triggering the reaction at the hydrogen transfer step is predicted to be difficult. However, when H_2_S is replaced by HSiSH, the successive C-end and O-end activation of CO_2_ can proceed smoothly to produce HCOOH with appreciably lower Gibbs free energy barriers. What is the difference between H_2_S and HSiSH that accounts for these distinct chemical behaviors? I first note that the incorporation of Si into H_2_S forms Si-H bonds, and hydridic H^δ−^ may be generated by the electronegativity difference between the Si and H atoms. Interestingly, previous studies have shown that the presence of a negatively charged hydride is crucial for the activation of CO_2_ [24], even if it is from the lattice [25]. I thus computed the atomic net charge distribution for the HSiSH molecule (Table 1), which confirmed the hydridic character of the hydrogen bonded directly to silicon. In addition, the condensed dual descriptors are presented in the same table, which allow for rationalization of the reactivity in terms of the simultaneous knowledge of the nucleophilic and electrophilic sites within a molecule. It was found that one of the hydrogen atoms experiences a substantial change from electrophilic (in H_2_S) to nucleophilic (in HSiSH) with the incorporation of Si into H_2_S, and one new electrophilic site (Si) is created as well (Table 1 and Figure 5). The combination of these two sites is exactly the Si-H chemical bond, which initiates the C-end activation of CO_2_ that would otherwise be O-end activation without silicon incorporation.

It is worth mentioning here that much attention has been paid to the Si-H bond in the field of CO_2_ activation recently [26,27,28,29,30]. Another benefit from incorporating silicon into H_2_S is the formation of Si=S multiple bonds when the protonic H^δ+^ attacks HCOO. Not only can this avoid COS formation, but it also gives HCOOH as an important product [31] without requiring much extra energy. Considering that the activation energy of the rate-limiting step may be affected by the ^δ+^M − H^δ−^ bond polarity [32], lead, as a heavier element in the same group as silicon, was used instead to test this effect. Interestingly, our results demonstrated a 3.4 kcal·mol^−1^ decrease in activation energy. Although this effect may not be highly pronounced, it could contribute to the design and optimization of this kind of reaction.

Of course, the abovementioned species is not the only one to activate H_2_S in CO_2_ hydrogenation and may be replaced by appropriate clusters like “superatom” species [33,34,35,36] that mimic elements of the periodic table with similar or even greater functionalities. Studies in this field will be intriguing, considering that there is plenty of room at the bottom; that is, novel species with tailored reactivity can be discovered by finely tuning their size, shape, composition, and charge. Active surfaces mimicking the chemistry of silicon might also exist. A reassessment of the key factors of the CO_2_ hydrogenation reaction suggests that these surfaces should have a very weak interaction with CO_2_ but exhibit high reactivity toward H_2_S dissociation, so as to form species resembling HSiSH first. The fact that the bond dissociation energy of S-H in H_2_S is ~381 kJ·mol^−1^ [37] is much smaller than that of C=O in CO_2_ (~750 kJ·mol^−1^) [38] indicates that this criterion is not difficult to meet. If the surface atom (or certain species deposited onto the surface) is capable of polarizing hydrogen to produce hydridic H^δ−^ while at the same time holding sulfur throughout the reaction, the C-end activation of CO_2_ and subsequent O-end activation to produce HCOOH can be expected. Clearly, surface defects, doping, or alloying would contribute further to the reactivity.

## 4. Computational Methods

The quantum chemical calculations consisted mainly of two parts: low-lying isomer predictions and PES construction for the gas-phase reaction of H_2_S and HSiSH with CO*_x_*. First, to obtain the global minimum and some low-lying isomers, a global (or local) search based on a particle swarm optimization algorithm within an evolutionary scheme was carried out using the CALYPSO package [39]. Owing to the interfacing of this package with Gaussian [40], DFT calculations at the B3LYP/6-311+G(d) level of theory [41,42,43] were performed, which guaranteed the reliability and precision of our searches in the initial stage. To enhance the structural diversity and search efficiency, identical structures were then excluded by a structure fingerprinting technique with a bond characterization matrix [44]. For the obtained structures, further optimization at the M062X/aug-cc-pVTZ level of theory [45,46,47,48] was performed, which has been shown to provide good results for main group thermochemistry and kinetics with accuracy comparable to more sophisticated and expensive correlated molecular orbital methods [49,50]. Correlation of these optimized structures provides direct structural insight into the reaction mechanism, and the PES can then be constructed by exploring the lacking intermediates and TSs along the reaction coordinate. The presence of zero or a single imaginary frequency was used to identify all the stationary points as minima or TSs. To ascertain that the identified TSs connected reactants and products smoothly, intrinsic reaction coordinate (IRC) calculations [51] were performed. Finally, the energetics of the optimal path were further refined using the coupled cluster single and double substitution method with a perturbative treatment of triple excitations (CCSD(T)) [52]. Specifically, the M06-2X/aug-cc-pVTZ calculated vibrational frequencies were used to estimate the zero-point corrections of all the converged structures, and the zero-point-corrected CCSD(T)/aug-cc-pVTZ electronic energies (Gibbs free energies at zero temperature) at the M06-2X/aug-cc-pVTZ-optimized geometries were used for the presented analyses.

To understand the unique reactivity of HSiSH as compared to H_2_S in CO_2_ hydrogenation, the Fukui function [53], dual descriptor [54], and their condensed forms [55] of all the reactants were computed at the M062X/aug-cc-pVTZ level of theory. As one of the widely used local descriptors in conceptual DFT [56], the Fukui function is used here to assign a certain type of reactivity such as nucleophilicity (*f*^+^) or electrophilicity (*f*^−^) to a specific region within a molecule, while the dual descriptor can be interpreted as the difference between the Fukui functions for nucleophilic and electrophilic attacks (∆*f*). This latter descriptor thus gives a combination of both Fukui functions: it is negative for locations where an electrophilic attack is more probable than a nucleophilic attack and positive where nucleophilic attack is more probable. In this way, the new index is dual and can be used to simultaneously detect the electrophile or nucleophile behavior of a given atomic region in the molecule. In the present work, the values of the dual descriptor were obtained at the 0.01 a.u. isodensity surface and visualized with the GaussView software [57].

## 5. Conclusions

The current study reveals that it is kinetically infeasible for hydrogens in the form of H_2_S to transfer to CO*_x_* at low temperatures. However, when HSiSH is employed instead, successive C-end and O-end activations of CO_2_ can be achieved, with HCOOH as the final product. This observation can be well explained by the unique structure of the HSiSH molecule, in which Si-H^δ−^ with both electrophilic and nucleophilic sites can initiate the hydrosilylation of CO_2_, followed by S-H^δ+^ cleavage to facilitate the hydrogenation of HCOO. The strong tendency of Si to form a strong bond with sulfur contributes further to the release of HCOOH. Although this finding confirms only the role of the silicon atom in tuning the reactivity of H_2_S, future works will focus more on the underlying chemistry between atoms, clusters, and surface systems for investigations and improved understanding.

## Figures and Tables

**Table 1:** Hirshfeld charges, condensed Fukui functions (*f*^+^/*f*^−^), and condensed dual descriptors (∆*f*) for the CO_2_, H_2_S, and *cis*-/*trans*-HSiSH molecules.

Molecule	Atom	*q* (N) [a]	*q* (N + 1)	*q* (N − 1)	*f* ^+^	*f* ^−^	∆*f*
CO_2_	C	0.3618	−0.2960	0.5777	0.6578	0.2159	0.4419
	O	−0.1808	−0.3179	0.2112	0.1371	0.3920	−0.2549
H_2_S	H	0.0616	−0.0806	0.1720	0.1421	0.1104	0.0317
	S	−0.1231	−0.8021	0.6560	0.6789	0.7792	−0.1002
*cis*-HSiSH	H [b]	−0.1150	−0.1874	0.0262	0.0724	0.1412	−0.0688
	Si	0.2444	−0.3987	0.7966	0.6430	0.5523	0.0908
	S	−0.1938	-0.4296	0.0482	0.2358	0.2419	−0.0061
	H	0.0644	0.0187	0.1290	0.0457	0.0646	−0.0189
*trans*-HSiSH	H [b]	−0.1119	−0.1841	0.0205	0.0722	0.1324	−0.0602
	Si	0.2394	−0.4024	0.7784	0.6418	0.5390	0.1029
	S	−0.1880	−0.4252	0.0827	0.2373	0.2707	−0.0334
	H	0.0605	0.0149	0.1184	0.0456	0.0580	−0.0124

[a] *q* (N), *q* (N + 1) and *q* (N − 1) correspond to the charge values of each atom for neutral, anion, and cation, respectively. [b] The hydrogen atom that bonded with silicon.

## Data Availability

The data presented in this study are available in supplementary material.

## Supplementary Materials

The following are available online, Figure S1, Cartesian coordinates of stationary points and transition states in the reactions of H_2_S with CO; Figure S2, Intrinsic reaction coordinate calculation for the reactions of H_2_S with CO; Figure S3, Reaction profiles for the H_2_S + CO; Figure S4, Cartesian coordinates of stationary points and transition states in the reactions of H_2_S with CO_2_; Figure S5, Intrinsic reaction coordinate calculation for the reactions of H_2_S with CO_2_; Figure S6, Reaction profiles for the H_2_S + CO_2_; Figure S7, Cartesian coordinates of stationary points and transition states in the reactions of HSiSH with CO; Figure S8, Intrinsic reaction coordinate calculation for the reactions of HSiSH with CO; Figure S9, Reaction profiles for the HSiSH + CO; Figure S10, Cartesian coordinates of stationary points and transition states in the reactions of HSiSH with CO_2_; Figure S11, Intrinsic reaction coordinate calculation for the reactions of HSiSH with CO_2_; Figure S12, A brief summary (TS omitted) of C-end activation pathways for the reaction of HSiSH + CO_2_; Figure S13, C-end activation pathways for the reaction of HSiSH + CO_2_; Figure S14, A brief summary (TS omitted) of O-end activation pathways for the reaction of HSiSH + CO_2_; Figure S15, O-end activation pathways for the reaction of HSiSH + CO_2_; Figure S16, Double-end activation pathway for the reaction of HSiSH + CO_2_.

## Sample Availability

Samples of the compounds are not available from the author.
