# Supplementary material for: Hydrogenation of CO2 Promoted by Silicon-Activated H2S: Origin and Implications
Source: Molecules. 2020 Dec 24;26(1):50. doi: 10.3390/molecules26010050 (PMC7796234; doi:10.3390/molecules26010050)
Supplement: Supplementary file 1 [file molecules-26-00050-s001.pdf]

Supporting Information

Hydrogenation of CO_2 Promoted by Silicon-Activated H_2S: Origin and Implications

Xing Liu*

College of Chemistry and Chemical Engineering, Southwest University, Chongqing 400715, China;

Email Address: xingliu1986@swu.edu.cn (X.L.)

Table of Contents

General Remarks 1

Reactions of H_2S with CO

Part I Cartesian coordinates of stationary points and transition states 2

Part II Intrinsic reaction coordinate calculation (IRC) 5

Part III Reaction pathways of H_2S + CO 8

Reactions of H_2S with CO_2

Part I Cartesian coordinates of stationary points and transition states 13

Part II Intrinsic reaction coordinate calculation (IRC) 16

Part III Reaction pathways of H_2S + CO_2 19

Reactions of HSiSH with CO

Part I Cartesian coordinates of stationary points and transition states 22

Part II Intrinsic reaction coordinate calculation (IRC) 26

Part III Reaction pathways of HSiSH + CO 30

Reactions of HSiSH with CO_2

Part I Cartesian coordinates of stationary points and transition states 39

Part II Intrinsic reaction coordinate calculation (IRC) 51

Part III Reaction pathways of HSiSH + CO_2 64

General Remarks

This Supplementary Material contains mainly three parts. The first part shows the geometries (given in cartesian coordinates) of the stationary points and transition states explored in the current study. The second part summarizes the intrinsic reaction coordinate calculation (IRC) which has been used extensively in checking the transition state (TS) geometries that linked the desired reactants and products along the reaction paths. Reasonable reaction paths are illustrated in the third part, one should mention here that the associated energies are reported at the **M06-2X/aug-cc-pVTZ** level. Due to the complexity of CO_2 reaction networks, a brief summary (TS omitted) of C-end activation and O-end activation pathways are given in previous for clarity. In addition, the following symbols are used to give a full picture for certain pathway.

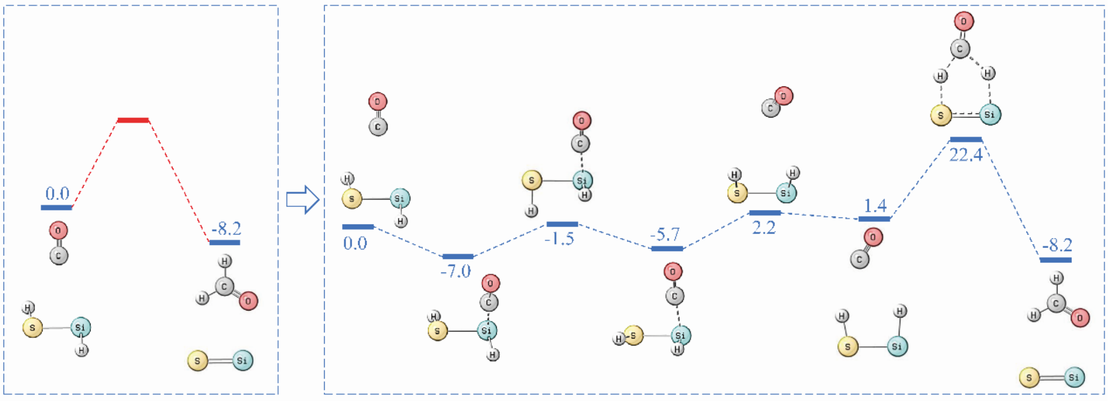

Potential Energy Surface (PES) in red indicates a multiple-step reaction, the details of which have been shown previously or are given in the following section.

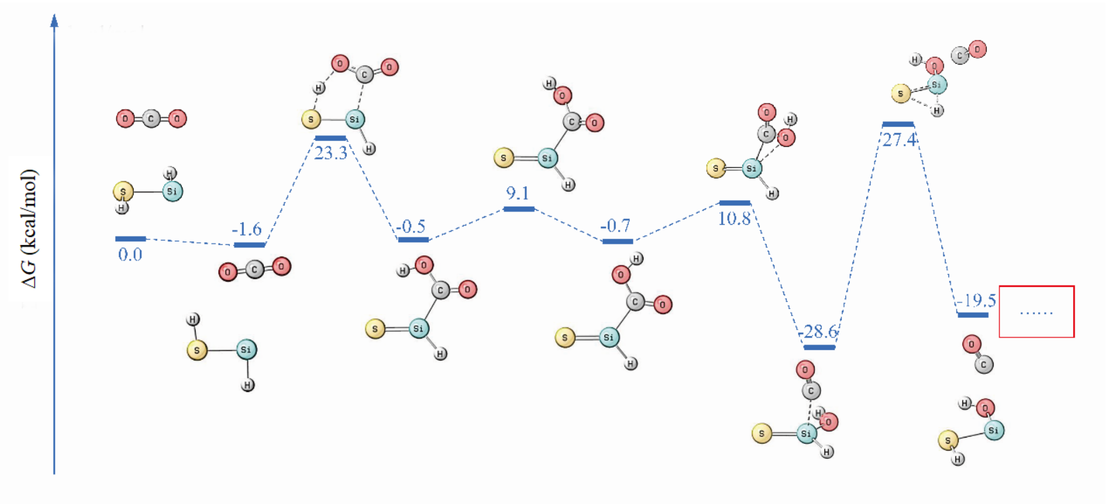

The ellipsis as marked by the red box indicates an incomplete PES, the rest of which is given in the following (or previous) section.

Reactions of H_2S with CO: Part I Cartesian coordinates of stationary points and transition states

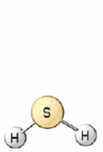

S	1.61763700	-0.10414600	0.02336300
H	2.93630400	-0.04376100	-0.20496600
H	1.45742100	1.21042500	-0.16816000
C	-1.70415000	0.48038400	0.04996500
O	-2.50637700	-0.29783000	-0.03756000

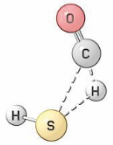

C	-1.07317200	0.48015700	-0.07064300
O	-1.85091400	-0.33318400	0.06301700
H	1.09656700	-0.67393100	-1.09331400
H	-0.18580000	1.15701300	-0.26743500
S	1.27097400	-0.04365900	0.08002900

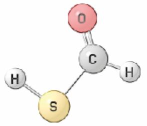

C	0.67412300	0.43479900	0.00004400
O	1.58693600	-0.33221400	-0.00004500
H	-0.81172100	-1.33097400	0.00042700
H	0.80043100	1.52836900	0.00021500
S	-1.04555800	-0.00928000	-0.00003400

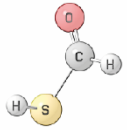

C	-0.71395100	0.42669900	0.01070700
O	-1.60715800	-0.34999500	-0.00564600
H	1.14157700	-0.18051300	1.25010100
H	-0.86625900	1.51486100	0.06038900
S	1.05410400	-0.06841100	-0.08309800

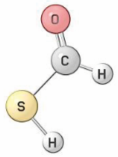

C	-0.67034000	0.42726800	-0.00001000
O	-1.60962100	-0.30565500	0.00005600
H	1.56625700	1.04750000	0.00064300
H	-0.75618500	1.52447400	-0.00040400
S	1.00555800	-0.16814600	-0.00003900

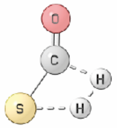

C	0.58482000	0.19608600	0.00011100
O	1.65764500	-0.26012000	0.00024100
H	-0.35613000	1.41466600	0.00004300
H	0.63510800	1.60029600	0.00013400
S	-1.06556600	-0.13190700	-0.00017300

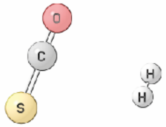

C	-0.43305300	-0.27515100	0.00134000
O	-1.56238300	-0.48392500	0.00426300
H	-0.96753200	2.73730500	0.16083400
H	-1.62601300	2.63368400	-0.15990100
S	1.10568300	0.00945700	-0.00269200

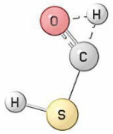

C	0.59083500	-0.52315900	0.00022700
O	1.54542000	0.34415700	-0.00006600
H	-0.77683700	1.31680200	0.00045600
H	1.79243800	-0.82881300	-0.00035000
S	-1.05774800	-0.00639300	-0.00005900

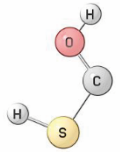

C	-0.55142400	-0.64817200	0.00000500
O	-1.45616200	0.30939100	-0.00000500
H	0.76753300	1.34074600	0.00002400
H	-2.32671100	-0.10571300	0.00001100
S	1.03231300	0.01118000	-0.00000200

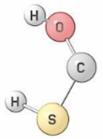

C	0.52438900	-0.69405600	0.02163900
O	1.52202900	0.21608700	-0.11058000
H	-0.72264500	1.34944600	-0.11215200
H	1.80137800	0.58065600	0.73802900
S	-1.02508200	0.03159600	0.00805800

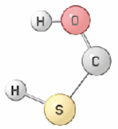

C	0.55621600	-0.67329000	0.00020400
O	1.55390300	0.16639900	-0.00005000
H	-0.76178800	1.35549400	0.00108700
H	1.30945800	1.11231100	-0.00050700
S	-1.01976200	0.01504600	-0.00008800

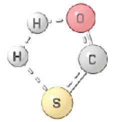

C	-0.48182000	-0.60754200	0.00007900
O	-1.49597000	0.07426700	-0.00005400
H	0.05267800	1.50091500	0.00009000
H	-1.00943600	1.24076600	0.00015300
S	0.98846500	0.01934000	-0.00001700

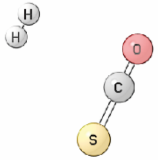

C	-0.43309100	-0.27687100	0.00132300
O	-1.56216600	-0.48710400	0.00425200
H	-0.96461300	2.75512400	0.16114600
II	-1.62357700	2.65582300	-0.15995500
S	1.10525400	0.00919500	-0.00269600

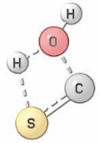

C	0.37930600	0.82884400	-0.00549100
O	1.36919000	-0.26494800	-0.07346100
H	0.45122000	-1.02978600	0.06881400
II	2.18977400	-0.10323500	0.41910100
S	-0.99189700	-0.10752900	0.00829500

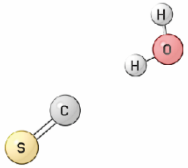

C	-0.21924000	0.12010000	0.00153200
O	2.95964100	-0.13143900	-0.00018200
H	3.35442300	0.74191800	-0.00200500
H	2.00700800	0.01851300	0.00083400
S	-1.73269500	-0.02684500	-0.00041000

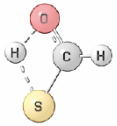

C	0.56620400	0.55490600	-0.00000500
O	1.43463000	-0.34842300	0.00007700
H	0.29426300	-1.07795300	0.00007400
II	0.84457600	1.60792500	-0.00005000
S	-1.00081900	-0.06700200	-0.00003800

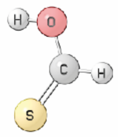

C	0.00000000	0.65621400	0.00000000
O	1.30782300	0.87252700	0.00000000
H	-0.53497900	1.60233300	0.00000000
S	-0.73047400	-0.78346400	0.00000000
H	1.75997400	0.01559900	0.00000000

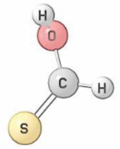

C	-0.40568400	0.48009400	0.01215900
O	-1.54086800	-0.25761300	-0.11595600
H	-0.59739700	1.55187000	0.06338800
S	1.07942200	-0.11730200	0.00316100
H	-1.91231100	-0.49469400	0.74073300

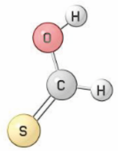

C	0.00000000	0.61330000	0.00000000
O	1.32517500	0.77292900	0.00000000
H	-0.55860500	1.54994400	0.00000000
S	-0.72492300	-0.82010200	0.00000000
H	1.55598000	1.70846000	0.00000000

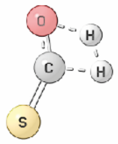

C	0.00000000	0.49838200	0.00000000
O	0.93884300	1.32383700	0.00000000
H	-1.11876500	1.28300400	0.00000000
S	-0.38141100	-1.04454300	0.00000000
H	-0.28940600	1.84870300	0.00000000

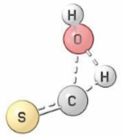

C	-0.14445100	0.66523500	0.04097500
O	-1.80694300	-0.22219500	-0.11499300
H	-1.28527100	0.96985900	0.13294300
S	1.15510300	-0.15176100	-0.00911400
H	-1.87411700	-0.75552300	0.68697300

Figure S1. Cartesian coordinates of stationary points and transition states in the reactions of H_2S with CO.

Reactions of H_2S with CO: Part II Intrinsic reaction coordinate calculation (IRC)

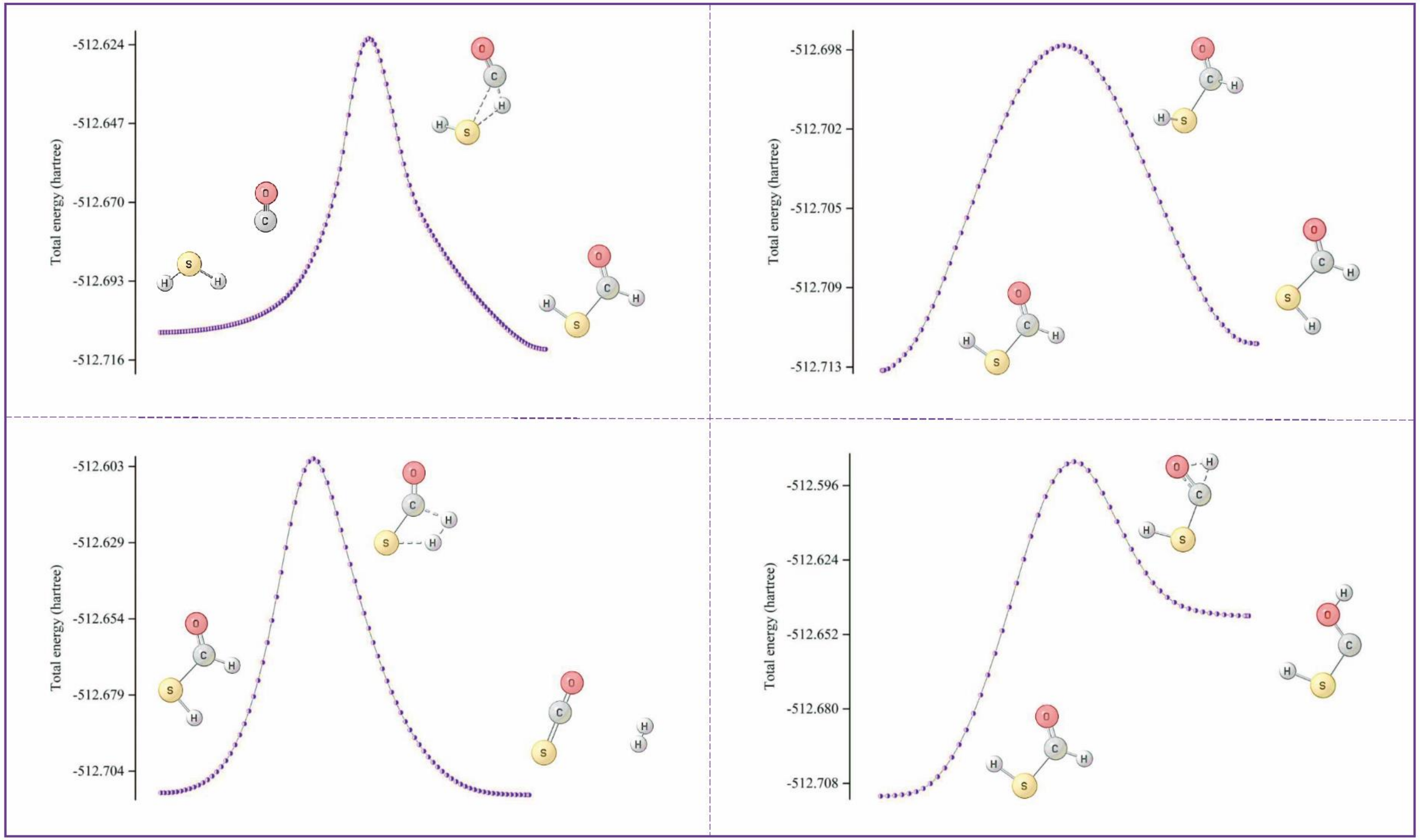

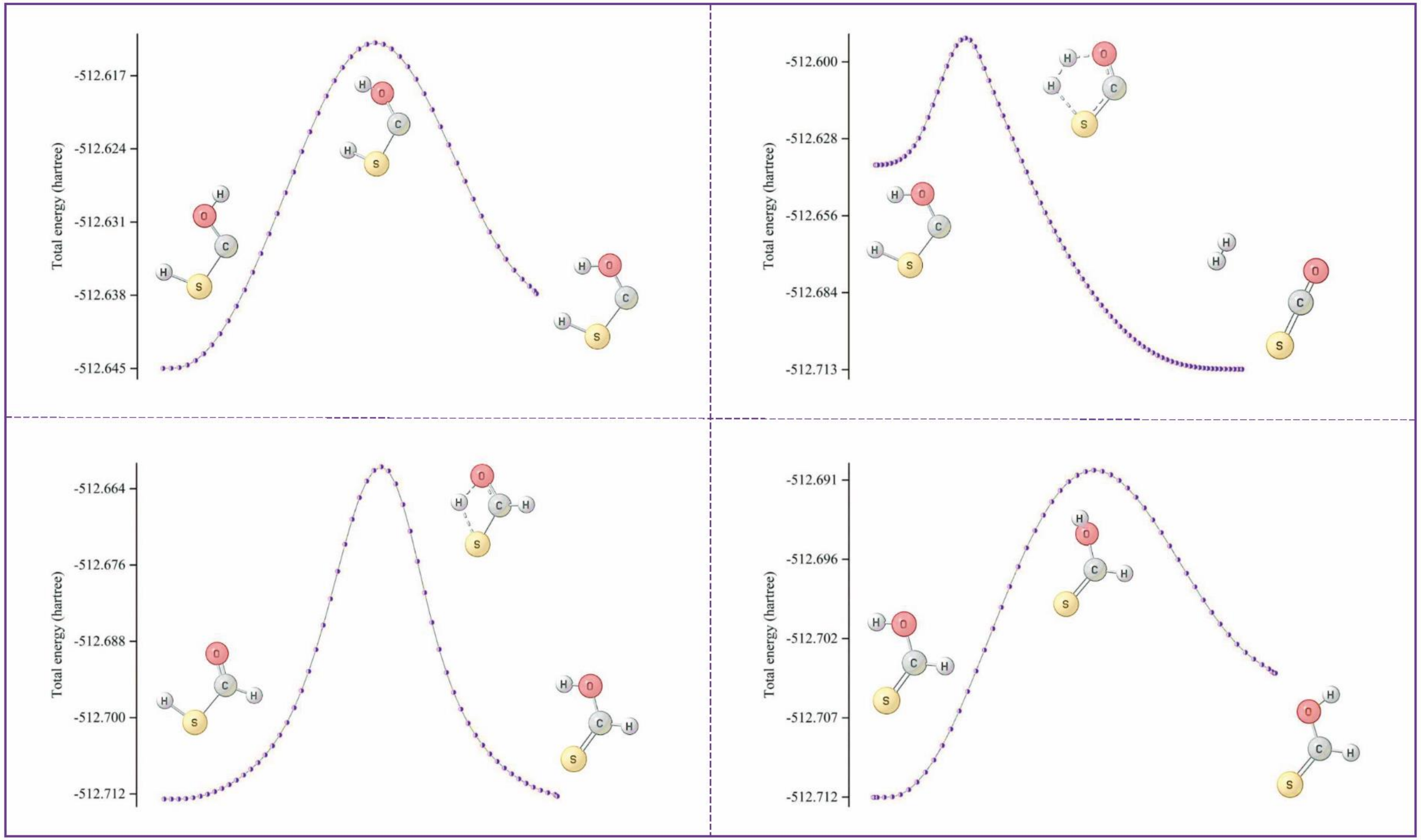

Total energy (hartree)
-512.617
-512.624
-512.631
-512.638
-512.645
-512.600
-512.628
-512.656
-512.684
-512.713
-512.664
-512.676
-512.688
-512.700
-512.712
-512.691
-512.696
-512.702
-512.707
-512.712

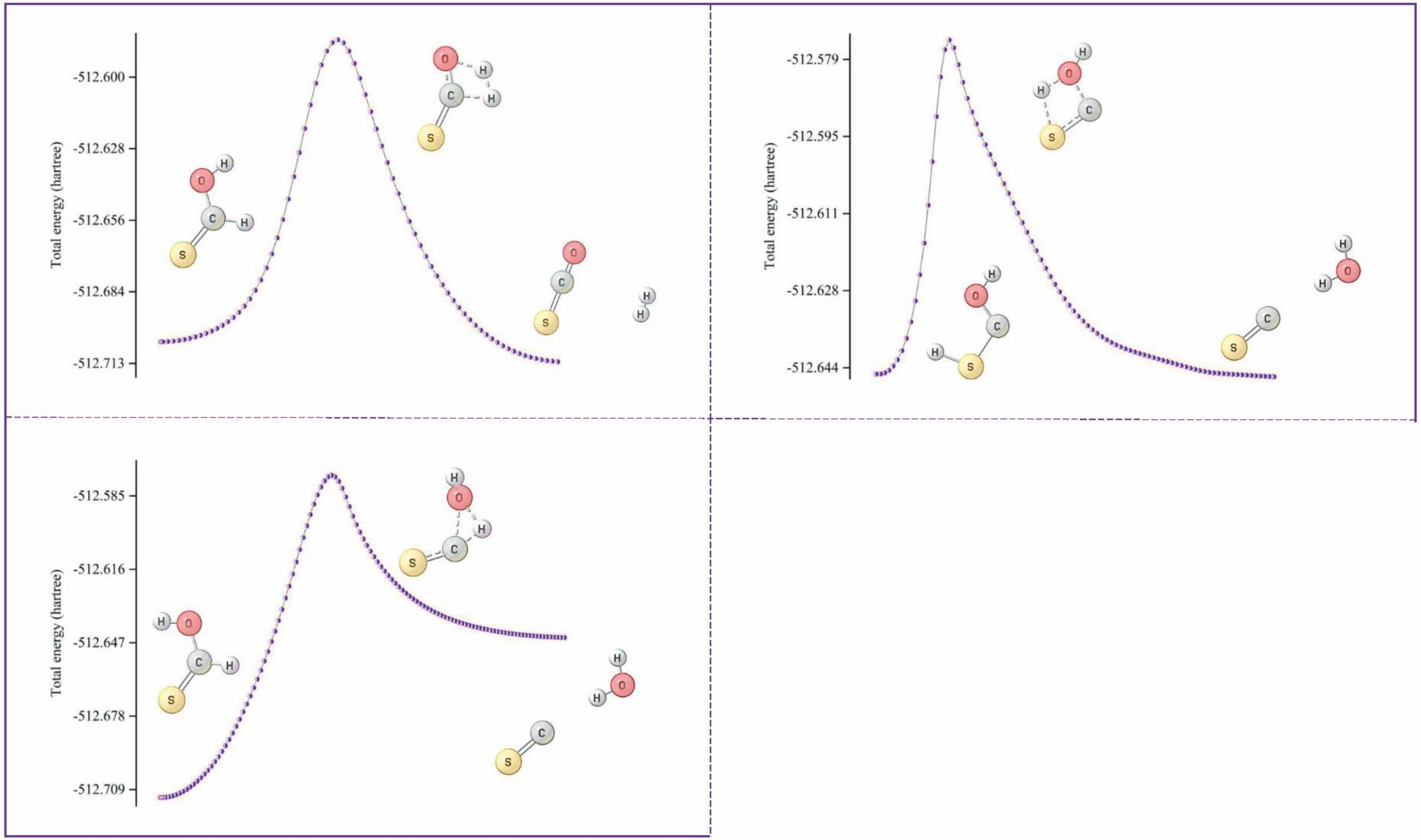

Figure S2. Intrinsic reaction coordinate calculation for the reactions of H_2S with CO.

Reactions of H_2S with CO: Part III Reaction pathways of H_2S + CO

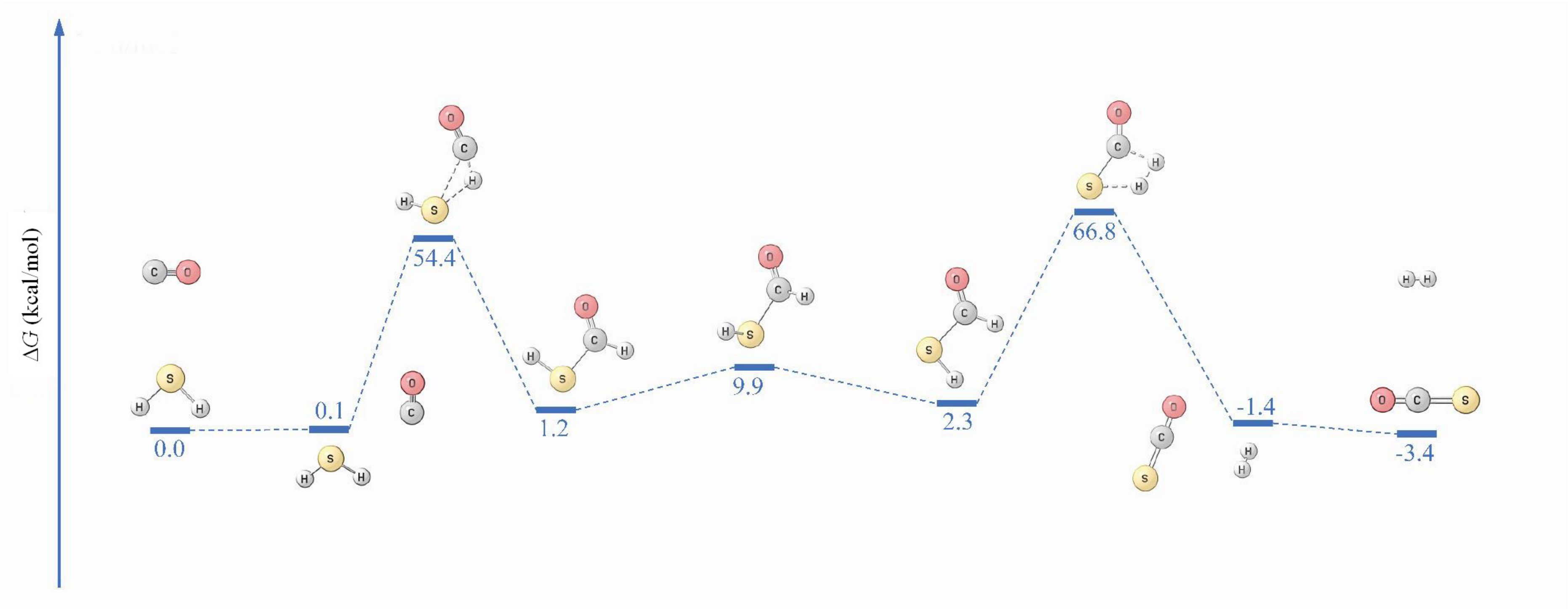

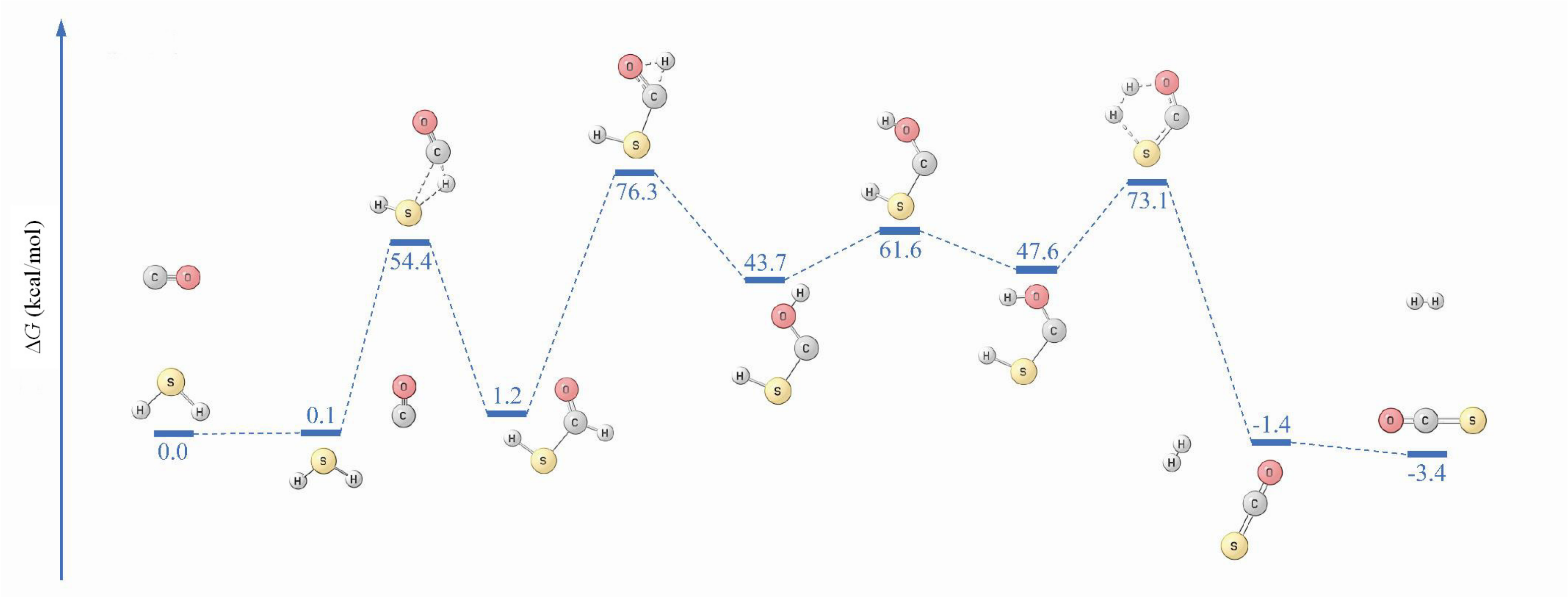

ΔG (kcal/mol)
0.0
0.1
54.4
1.2
76.3
43.7
61.6
47.6
73.1
-1.4
-3.4

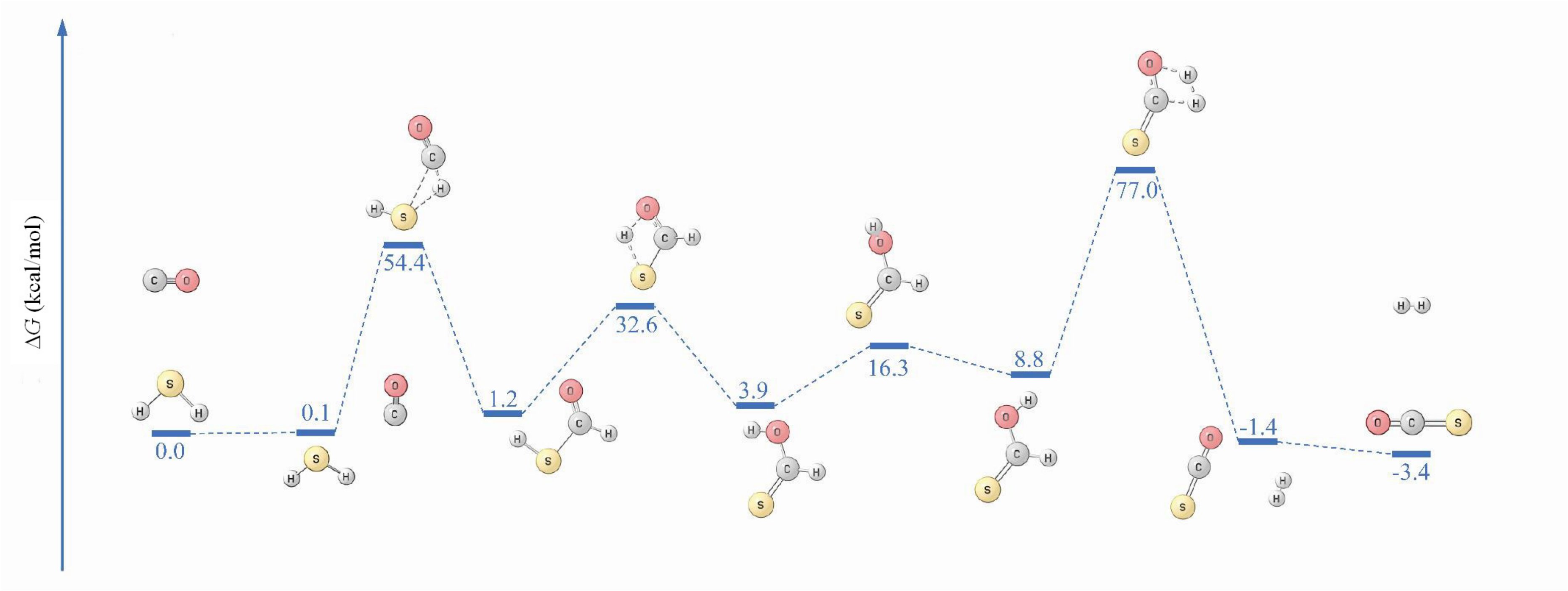

ΔG (kcal/mol)
0.0
0.1
54.4
1.2
32.6
3.9
16.3
8.8
77.0
-1.4
-3.4

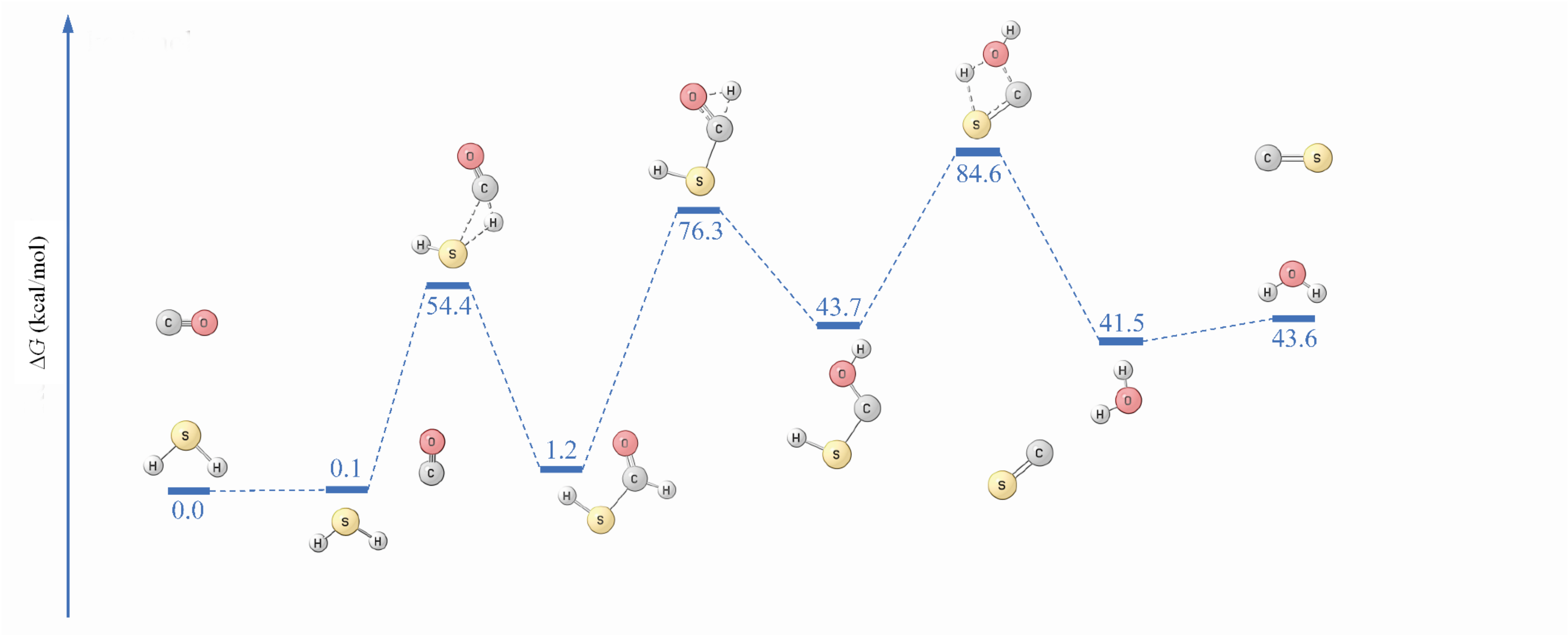
ΔG (kcal/mol)
0.0
0.1
54.4
1.2
76.3
43.7
84.6
41.5
43.6

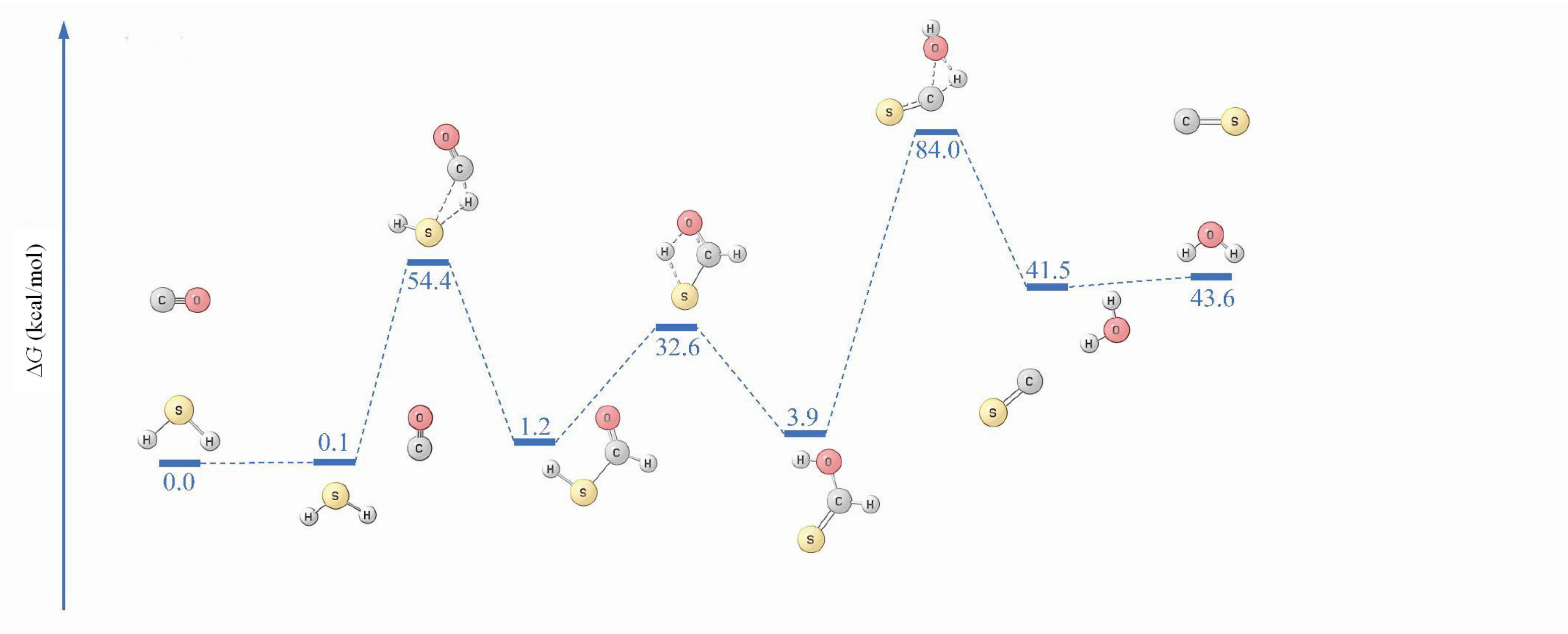

Figure S3. Reaction profiles for the H_2S + CO.

Reactions of H_2S with CO_2: Part I Cartesian coordinates of stationary points and transition states

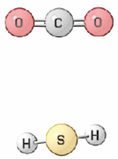

C	1.48869400	0.01606300	-0.00140000
O	1.58339800	-1.13493900	-0.00071100
O	1.41743100	1.16995800	0.00406800
H	-1.69030500	1.05003900	0.66555100
H	-1.98629300	-0.83975200	0.95674500
S	-1.82888800	-0.03667600	-0.10254700

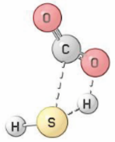

C	0.86213500	-0.01473700	-0.00543500
O	1.52806200	-0.96124700	-0.00195800
H	-1.45255800	-0.29006400	1.25303000
O	0.80604600	1.22800600	0.01352700
S	1.37211800	-0.18195500	-0.08094600
H	-0.43922300	1.15569600	-0.01784600

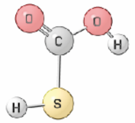

C	0.00000000	0.53320400	0.00000000
O	1.29084000	0.90253500	0.00000000
H	1.88420600	0.14377200	0.00000000
O	-0.88517900	1.32216600	0.00000000
S	-0.22317700	-1.24884000	0.00000000
H	-1.55866000	-1.15915800	0.00000000

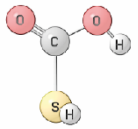

C	0.54562200	-0.13143300	0.00619500
O	1.15375900	1.05969700	-0.00535800
H	0.49535200	1.76135800	0.04631400
O	1.13505600	-1.15501200	-0.00878200
S	-1.29296900	-0.01761600	0.08022000
H	-1.39209200	0.07161700	-1.25388800

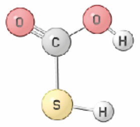

C	0.00000000	0.53513900	0.00000000
O	1.28893600	0.90944100	0.00000000
H	1.88659900	0.15725300	0.00000000
O	-0.88638100	1.32254800	0.00000000
S	-0.37399200	-1.22001100	0.00000000
H	0.87683300	-1.70382500	0.00000000

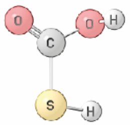

C	-0.49698600	-0.15046300	-0.01191100
O	-1.14383600	1.05065300	-0.10114000
H	-1.27033700	1.43009200	0.77585100
O	-1.05144100	-1.19762700	0.00533400
S	1.27961000	-0.03981700	0.01409400
H	1.34071300	1.28555200	-0.16343700

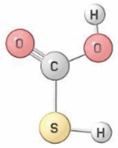

C	-0.48079800	-0.12958300	0.00050400
O	-1.05131800	1.08679900	-0.00016800
H	-2.00909500	0.94582800	-0.00012900
O	-1.08550200	-1.15786400	-0.00009000
S	1.28902300	-0.05513200	-0.00010300
H	1.36406900	1.28230400	0.00081800

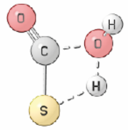

C	0.42876700	-0.29794800	0.02091300
O	0.80039900	1.22102100	0.09443900
H	1.42547400	1.47588800	-0.60134800
O	1.29317400	-1.08636200	-0.00542100
S	-1.27100400	-0.12848400	-0.01146000
H	-0.41059700	1.29026200	-0.05290800

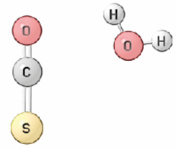

C	-0.41154600	0.61052300	-0.00020200
O	2.18650000	-0.70202300	0.01770800
H	2.46771000	0.21436200	-0.03832600
O	0.33846500	1.48376200	-0.00018600
S	-1.44881900	-0.55652500	-0.00119900
H	2.98296000	-1.22700900	-0.08145300

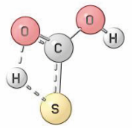

C	0.00000000	0.44829900	0.00000000
O	-1.00460900	1.29413100	0.00000000
H	-1.84376800	0.82107600	0.00000000
O	1.17773000	0.86655100	0.00000000
S	-0.05594100	-1.27036800	0.00000000
H	1.35384100	-0.47044300	0.00000000

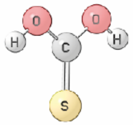

C	0.00000000	0.00000000	-0.33907400
O	0.00000000	1.07331800	-1.10866100
O	0.00000000	-1.07331800	-1.10866100
H	0.00000000	1.84821800	-0.53382900
H	0.00000000	-1.84821800	-0.53382900
S	0.00000000	0.00000000	1.30254200

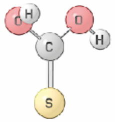

C	-0.31434900	0.00606000	-0.01405700
O	-1.13675200	-1.05393900	-0.10881500
O	-1.02142600	1.13041500	0.00756300
H	-1.40734300	-1.36793600	0.76113400
H	-0.40208600	1.87139900	0.03365800
S	1.31005900	-0.07197700	0.00622300

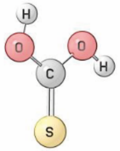

C	0.00000000	0.30286600	0.00000000
O	-1.02393000	1.13868400	0.00000000
O	1.15461400	0.97631600	0.00000000
H	-0.69973300	2.04971900	0.00000000
H	1.87330300	0.33168400	0.00000000
S	-0.13869000	-1.31991200	0.00000000

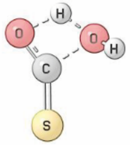

C	0.18484600	0.21453000	-0.01537900
O	1.24193600	-0.95888900	-0.10087200
H	1.15043700	-1.58474300	0.63034800
H	1.79214500	0.08634700	0.04711500
O	1.03394700	1.13000500	0.02671000
S	-1.39117000	-0.07235700	0.00050700

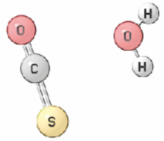

C	0.52919800	0.65295400	0.00182000
O	-2.16719200	-0.41768300	-0.03566800
H	-1.94967700	-1.34400400	0.08960600
H	-3.10568700	-0.34132800	0.14838500
O	0.10912900	1.71904500	0.00366400
S	1.14654200	-0.79020500	0.00044500

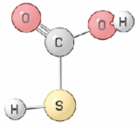

C	0.49083900	0.15839900	-0.01000800
O	1.17700600	-1.02022500	-0.10400900
H	1.33680000	-1.39275700	0.77035200
O	1.00573400	1.22557100	0.00935700
S	-1.26000800	-0.14676900	-0.00424500
H	-1.58363200	1.14789300	0.11483200

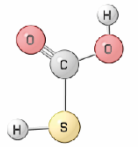

C	0.00000000	0.49500700	0.00000000
O	1.31013200	0.78109700	0.00000000
H	1.39203900	1.74525400	0.00000000
O	-0.87160400	1.30998700	0.00000000
S	-0.20968600	-1.26703600	0.00000000
H	-1.54528000	-1.17139300	0.00000000

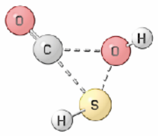

C	-0.97945600	-0.24863600	0.10932300
O	0.57236300	1.27089400	-0.09194500
H	0.84260000	1.63860900	0.75756800
H	0.53136500	-1.76364700	0.24210000
S	1.03196000	-0.51226400	-0.03242600
O	-2.07343600	-0.04425900	-0.05015200

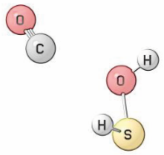

C	-1.88919000	-0.57386700	0.21449600
O	0.65583000	0.99741400	-0.02656100
H	0.75348600	1.41081800	0.83638000
H	0.71641700	-1.25113400	0.34874000
S	1.63134400	-0.35493700	-0.06337600
O	-2.68536300	0.12290000	-0.15569900

Figure S4. Cartesian coordinates of stationary points and transition states in the reactions of H_2S with CO_2.

Reactions of H_2S with CO_2: Part II Intrinsic reaction coordinate calculation (IRC)

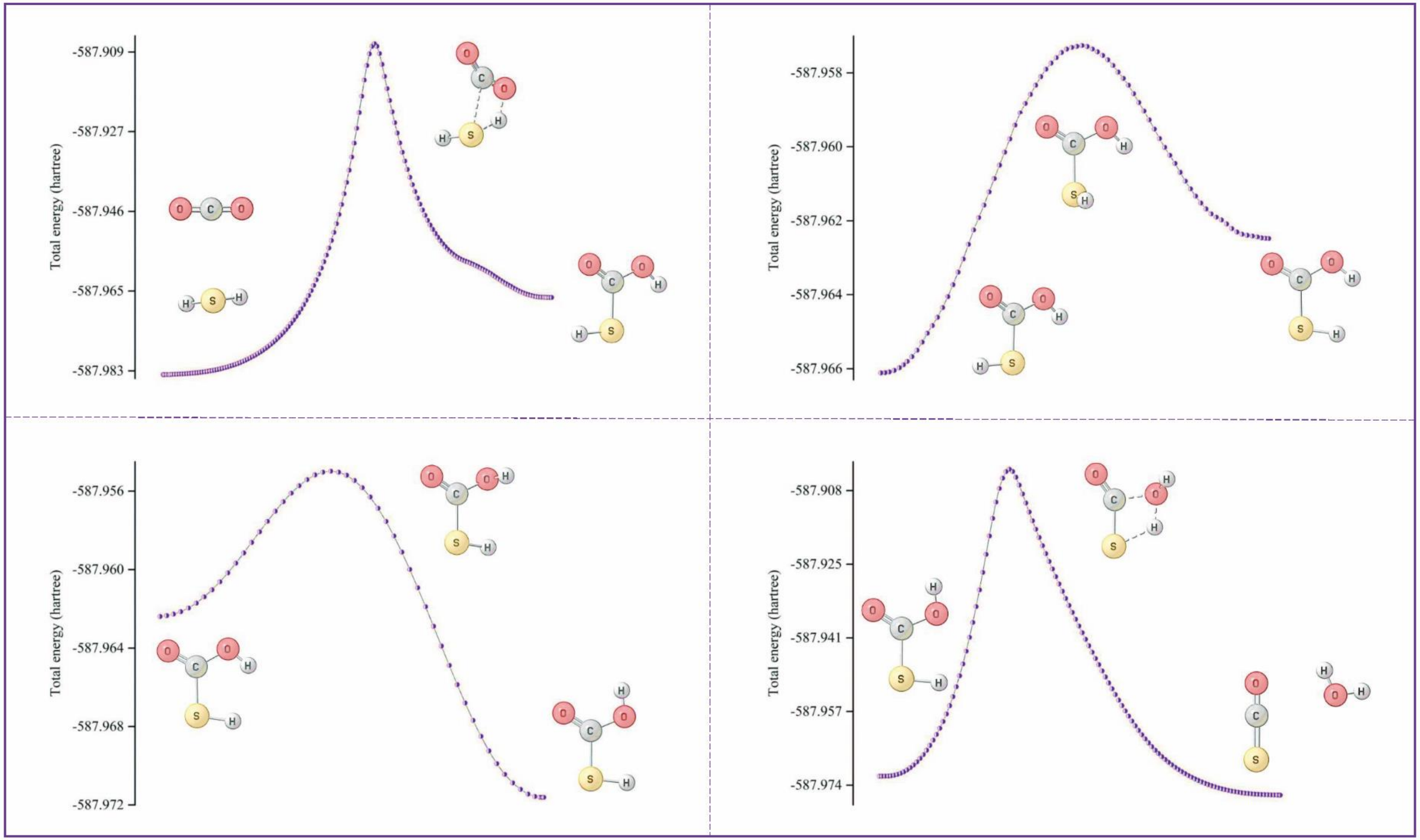

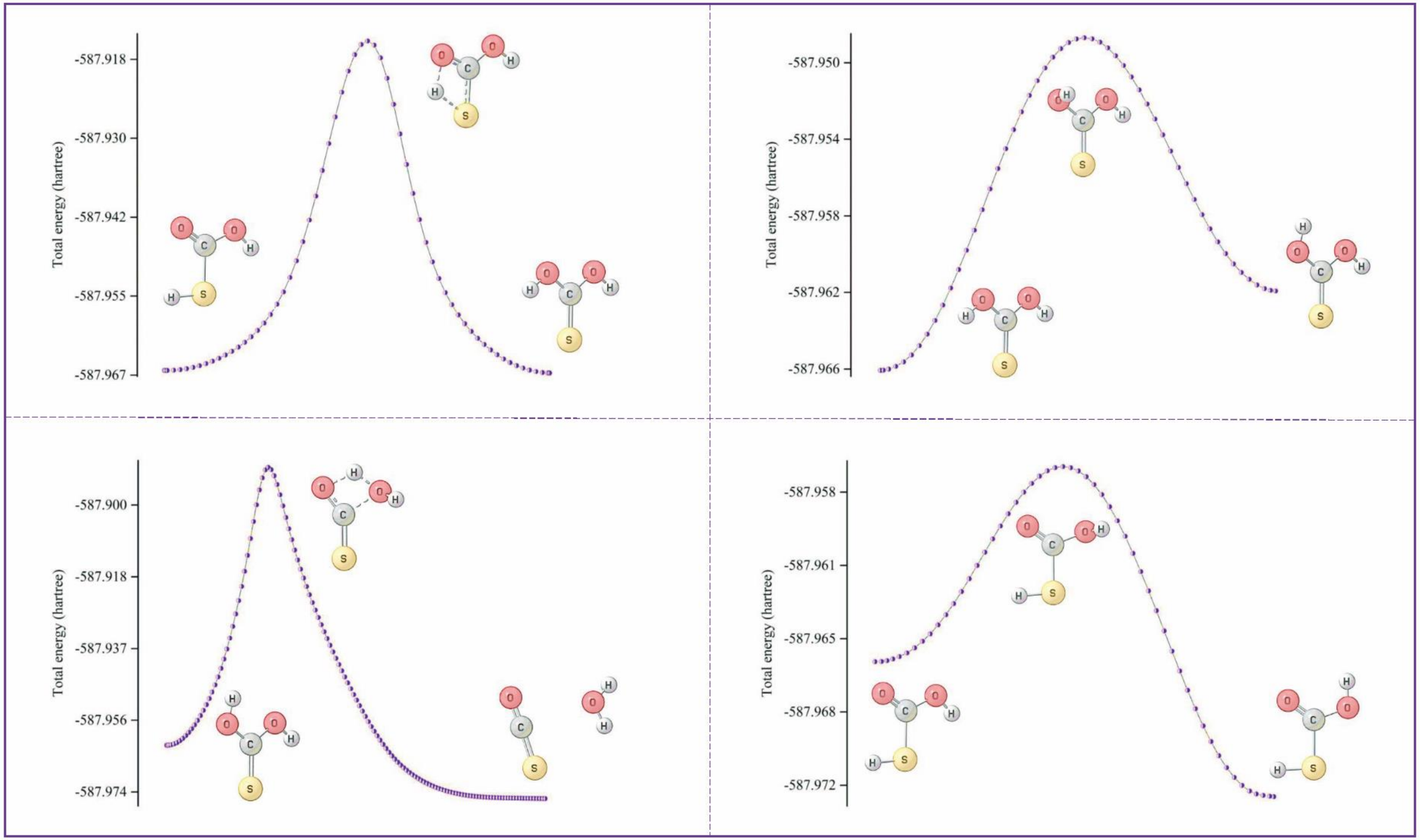

-587.918
-587.930
-587.942
-587.955
-587.967
Total energy (hartree)
-587.950
-587.954
-587.958
-587.962
-587.966
Total energy (hartree)
-587.900
-587.918
-587.937
-587.956
-587.974
Total energy (hartree)
-587.958
-587.961
-587.965
-587.968
-587.972
Total energy (hartree)

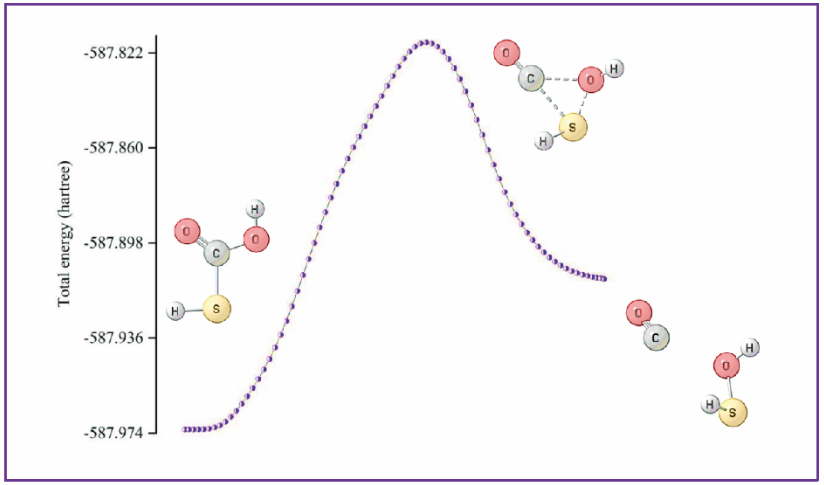

Figure S5. Intrinsic reaction coordinate calculation for the reactions of H_2S with CO_2.

Reactions of H_2S with CO_2:   Part III Reaction pathways of $H_2S + CO_2$

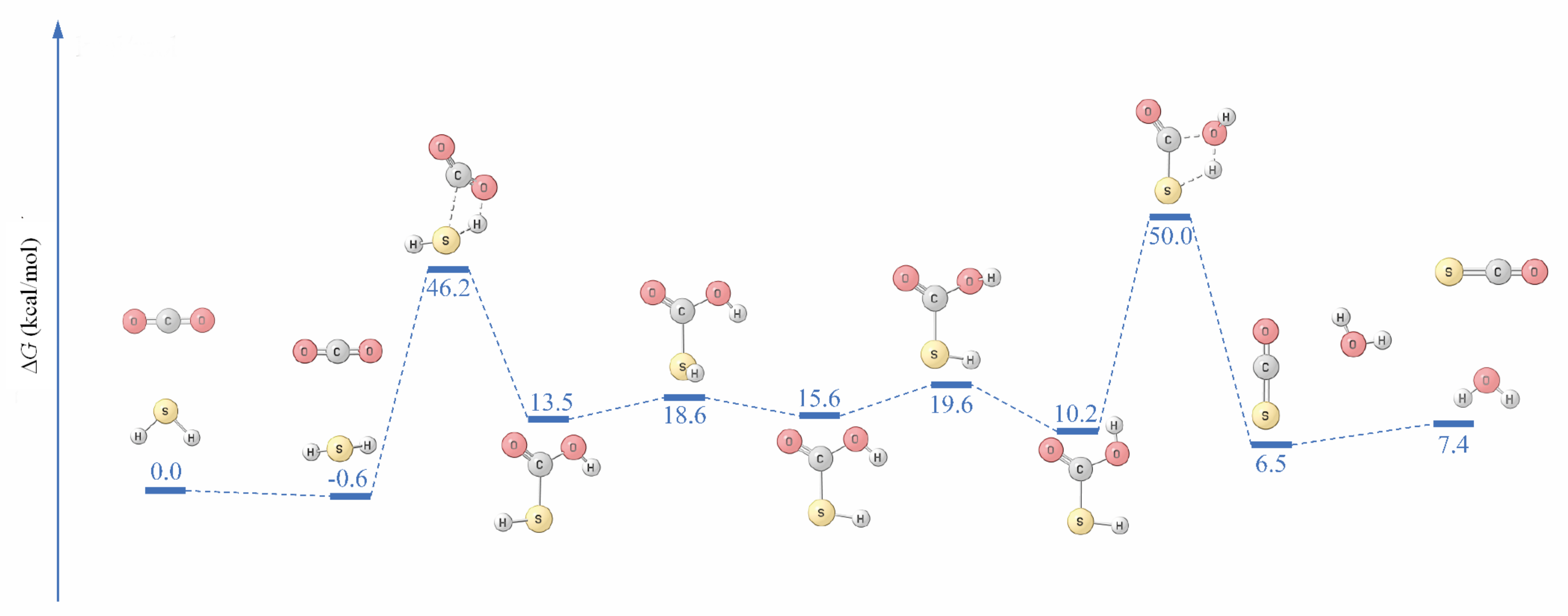

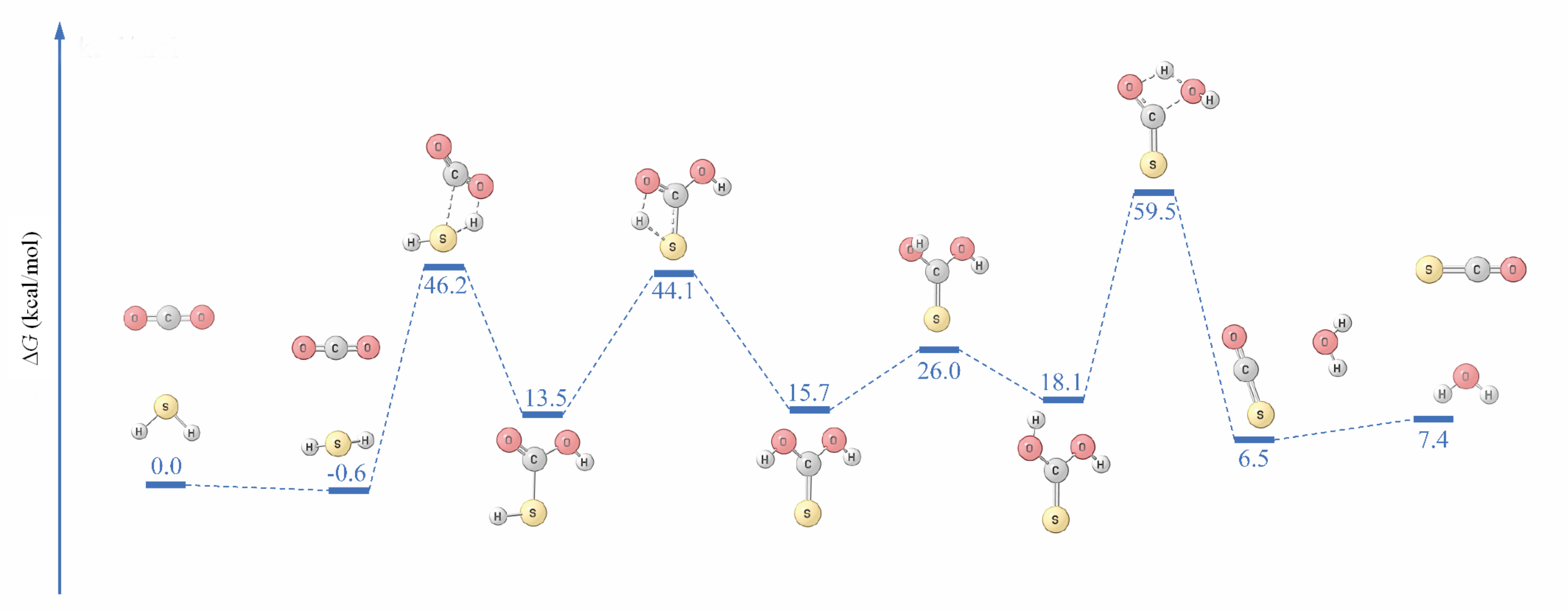
ΔG (kcal/mol)
0.0
-0.6
46.2
13.5
44.1
15.7
26.0
18.1
59.5
6.5
7.4

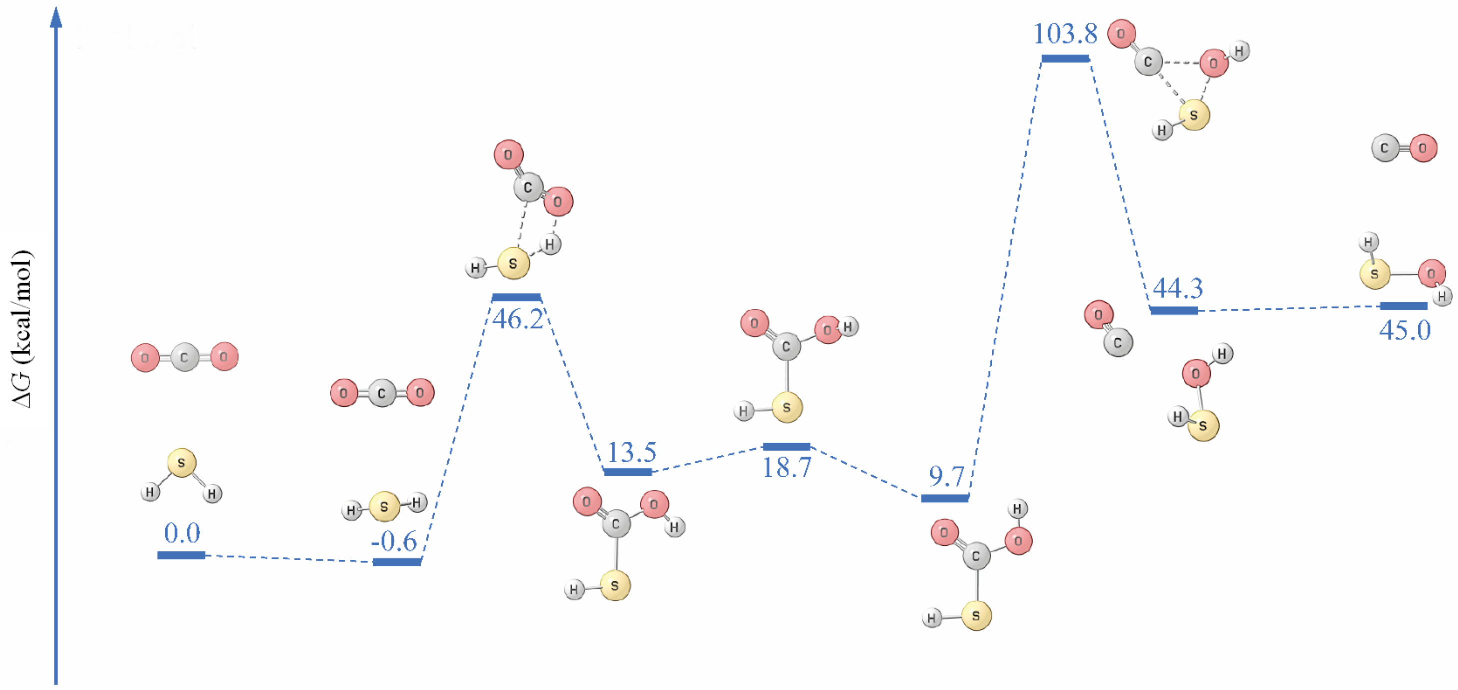

Figure S6. Reaction profiles for the H_2S + CO_2.

Reactions of HSiSH with CO: Part I Cartesian coordinates of stationary points and transition states

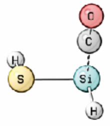

H	1.93053600	-0.56871900	-1.16213600
H	-0.05977700	1.36749100	1.35359600
C	-1.43195200	-0.06152100	0.04885700
O	-2.34147500	-0.71630300	-0.04318900
Si	0.14342400	1.10955800	-0.11558300
S	1.46530100	-0.63956500	0.09244300

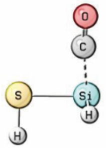

H	2.42777600	0.08897600	0.57112700
H	-0.16597400	1.28378700	1.35321800
C	-1.41858100	-0.09611500	0.04239100
O	-2.36428800	-0.70431500	0.00287400
Si	0.09867300	1.09811700	-0.13000700
S	1.48641000	-0.65844900	-0.02384900

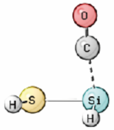

H	1.32457600	-1.02869500	1.19642600
H	-0.02610000	1.37772800	1.37241800
C	-1.41420800	-0.05924600	0.05927800
O	-2.31175600	-0.72959200	-0.04524700
Si	0.13776100	1.12819400	-0.10190500
S	1.48451000	-0.62197100	-0.07099200

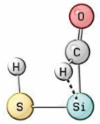

H	-1.06806100	0.31698500	1.25500400
H	0.84056600	-1.54620100	-0.42119500
C	-1.34886700	-0.03103400	0.08981500
O	-2.30439500	-0.69163500	-0.09288900
Si	0.11285400	1.09036200	-0.08311400
S	1.57349400	-0.51978500	0.03337500

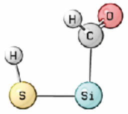

H	-1.23014900	-1.09313200	1.22526300
H	0.96136700	-1.53902800	0.24985000
C	-1.31339300	-0.34267900	0.40357800
O	-2.22545700	-0.41668100	-0.38268100
Si	0.02506200	1.03486800	0.04668400
S	1.60012000	-0.40415400	-0.09304400

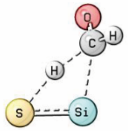

H	-1.89014200	-0.00830100	1.49978500
H	-0.10762400	-0.56379100	0.35520700
C	-1.49958200	-0.13067000	0.46709300
O	-2.20744600	-0.50458800	-0.42679500
Si	0.27675700	1.03970000	-0.08221500
S	1.54876400	-0.57268600	-0.00576100

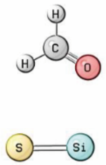

H	3.13872400	-1.00192000	-0.00024200
H	1.29884600	-1.43654700	0.00076700
C	2.09041700	-0.67169700	0.00003500
O	1.81903300	0.50035600	-0.00009400
Si	-0.71224600	1.06549200	0.00026600
S	-1.34755600	-0.77819300	-0.00023100

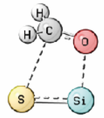

H	1.27379400	-1.50384800	0.93389900
H	1.27379600	-1.50384900	-0.93389700
C	1.36358500	-0.94315600	0.00000100
O	1.69424100	0.24816600	0.00000100
Si	-0.23243300	1.12009800	0.00000000
S	-1.31431000	-0.56250400	-0.00000100

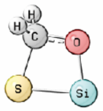

H	0.62265700	1.77272900	0.89369200
H	0.62312300	1.77315000	-0.89286000
C	0.35219500	1.21485000	0.00021000
O	-1.02267700	0.89781800	-0.00028600
Si	-0.98537600	-0.78226000	0.00010300
S	1.16360800	-0.44161800	-0.00007800

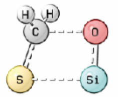

H	0.50637200	1.64540000	-0.91592200
H	0.50783400	1.64609300	0.91596900
C	0.65942300	1.08971000	0.00009900
Si	-1.17885200	-0.72885200	0.00009800
S	1.45152800	-0.37468000	-0.00006600
O	-1.46140800	0.79613200	-0.00011900

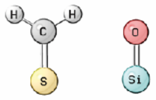

H	-0.46893500	1.57133600	0.00217400
H	-2.34619700	1.58223700	0.00098900
C	-1.41453900	1.02506500	0.00056700
Si	1.53745000	-0.55583500	0.00055000
S	-1.41487300	-0.57879900	-0.00038900
O	1.55200500	0.96731300	-0.00100600

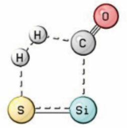

H	0.70271600	-1.59891900	0.00011900
H	-0.23557100	-1.59487700	0.00011200
C	1.40330400	-0.28215800	0.00002100
O	2.54701100	-0.24028900	0.00000700
Si	-0.31301700	1.07014300	-0.00006700
S	-1.55505100	-0.51081000	0.00003300

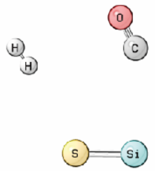

H	1.63384700	3.28218700	0.00123700
H	1.10601600	2.76407000	0.00132200
C	2.20520600	-0.62519500	-0.00009700
O	3.12308300	0.01723000	-0.00016900
Si	-1.21966900	-1.10191200	0.00028100
S	-1.49252500	0.81211500	-0.00028600

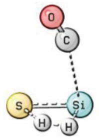

Si	0.94143300	1.08820500	-0.12887900
S	1.51719400	-0.89570300	-0.03292300
H	0.37290300	0.91653000	1.46163500
H	0.74802600	0.00229900	1.19898600
C	-2.23628200	0.24323500	0.00358300
O	-3.14480000	-0.41023400	-0.04388200

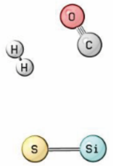

Si	-1.20310500	-1.04927100	0.25921800
S	-1.47298600	0.78090000	-0.30195100
H	1.42711700	2.58922700	1.77373000
H	0.91688700	2.09876300	1.55847600
C	2.21951100	-0.53812600	0.02298600
O	3.09377200	0.09202000	-0.28349500

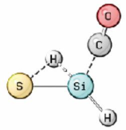

Si	-0.15663900	0.88737500	0.08569900
S	-1.62041000	-0.57042700	-0.06923500
H	-0.62573100	0.07862000	1.33617100
H	0.07957800	1.99592700	-0.86486400
C	1.51059100	-0.03677900	-0.12028000
O	2.45026500	-0.64378500	0.01979300

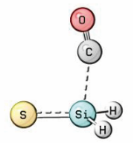

Si	0.40746000	0.92807800	0.00008500
S	1.43906800	-0.75016900	-0.00001800
H	0.14465900	1.75974900	1.19447300
C	-1.59096200	0.09587500	-0.00053600
O	-2.43431100	-0.63580300	0.00018500
H	0.14607500	1.76103000	-1.19364800

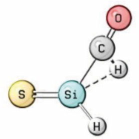

Si	-0.16427300	0.63573200	-0.05378100
S	-1.79632000	-0.42396100	0.00705400
H	0.15154600	2.06925300	-0.08580900
C	1.52637700	-0.04971900	-0.00551000
O	2.57949600	-0.53252200	-0.05799400
H	1.09517900	0.37236700	1.22288500

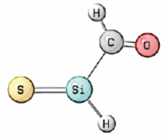

H	0.31093400	1.92036700	0.00041500
C	1.51502900	-0.55723800	-0.00012600
O	2.57818900	0.00420000	-0.00008300
Si	-0.09482000	0.50054700	0.00010100
S	-1.88429000	-0.24727300	-0.00000900
H	1.44949900	-1.66182500	-0.00027100

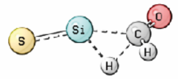

H	0.78758700	0.35527900	1.17928900
C	1.78795800	0.37707500	0.03484200
O	2.62577400	-0.47828200	0.04207700
Si	-0.13023600	0.08918600	-0.21953300
S	-2.03908400	-0.09394600	0.08171300
H	1.92713300	1.46305900	0.04109400

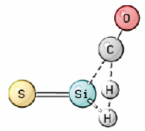

H	-0.42942200	1.64489000	0.91834500
H	-1.16639300	0.88974000	1.19688300
C	-1.60750300	-0.05147600	0.15085400
O	-2.59526600	-0.57047900	-0.09082500
Si	0.23812300	0.67976400	-0.25145800
S	1.79182700	-0.44866400	0.07666600

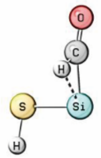

H	-1.06834400	0.26752000	1.26378900
H	2.52069600	0.14132700	0.39680600
C	-1.36110300	-0.04706800	0.09533700
O	-2.34906100	-0.65430900	-0.09788700
Si	0.11773900	1.05928900	-0.07543400
S	1.49115000	-0.60762600	-0.02459000

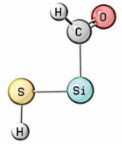

H	-1.44140100	-0.93095600	1.36862500
H	2.50565300	0.26690100	-0.47020200
C	-1.35304600	-0.30680600	0.45401000
O	-2.15684400	-0.43897100	-0.44070800
Si	0.01698300	1.01455200	0.02986700
S	1.50443800	-0.51169200	-0.03218500

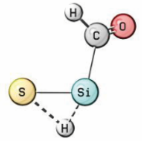

H	1.15116100	-1.22535300	-0.96257900
H	-1.10431900	1.02817500	1.07520300
C	1.39491000	-0.35881000	-0.29960100
O	2.40911600	-0.29113300	0.32206000
Si	-0.12083400	0.89108500	-0.13547800
S	-1.62484700	-0.48725600	0.06282400

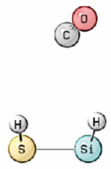

H	0.45123100	-1.36492800	0.65049400
H	-0.11618100	1.10000300	0.74102900
C	-2.44649200	-0.29045600	-0.40021900
O	-3.34157600	0.01772000	0.20083500
Si	1.17953100	1.18706800	-0.04076100
S	1.53519200	-0.92206600	-0.00164000

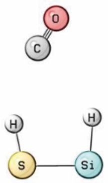

H	0.01405000	-1.20435200	0.05476100
H	-0.11220000	1.30058600	0.05827100
C	-2.38362800	-0.37237000	0.01203100
O	-3.32128900	0.24089700	-0.01145900
Si	1.39746400	1.14558700	-0.00178700
S	1.33785800	-0.98921300	-0.00428300

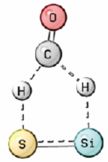

H	-0.51916700	-0.94318000	0.00011400
H	-0.61630700	1.01985100	0.00021500
C	-1.63606000	-0.11880600	0.00020500
O	-2.76259200	0.04894800	-0.00012700
Si	1.09418700	1.09107000	-0.00001500
S	1.10837200	-0.93940000	-0.00002100

Figure S7. Cartesian coordinates of stationary points and transition states in the reactions of HSiSH with CO.

Reactions of HSiSH with CO: Part II Intrinsic reaction coordinate calculation (IRC)

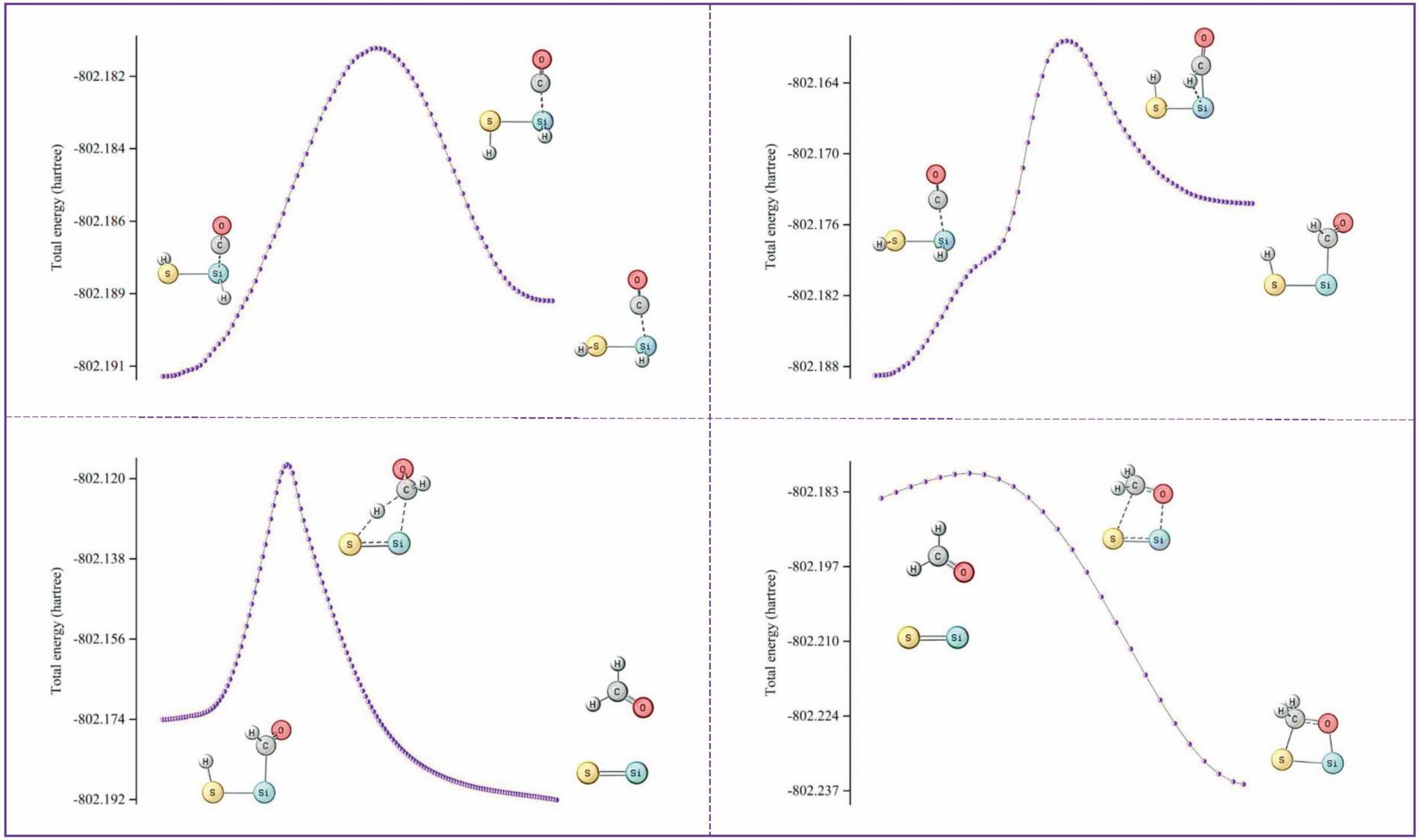

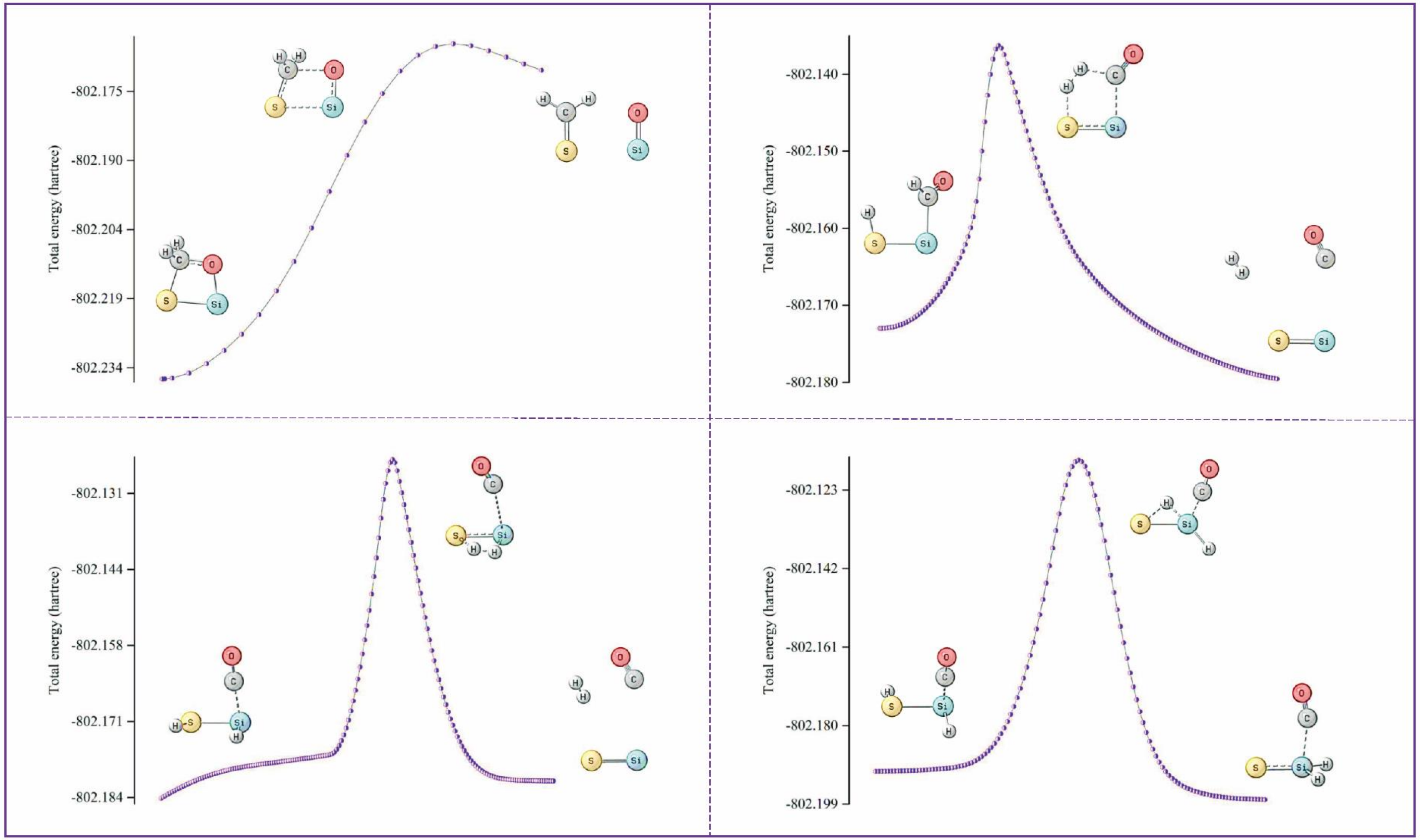

Total energy (hartree)
-802.175
-802.190
-802.204
-802.219
-802.234
-802.140
-802.150
-802.160
-802.170
-802.180
-802.131
-802.144
-802.158
-802.171
-802.184
-802.123
-802.142
-802.161
-802.180
-802.199

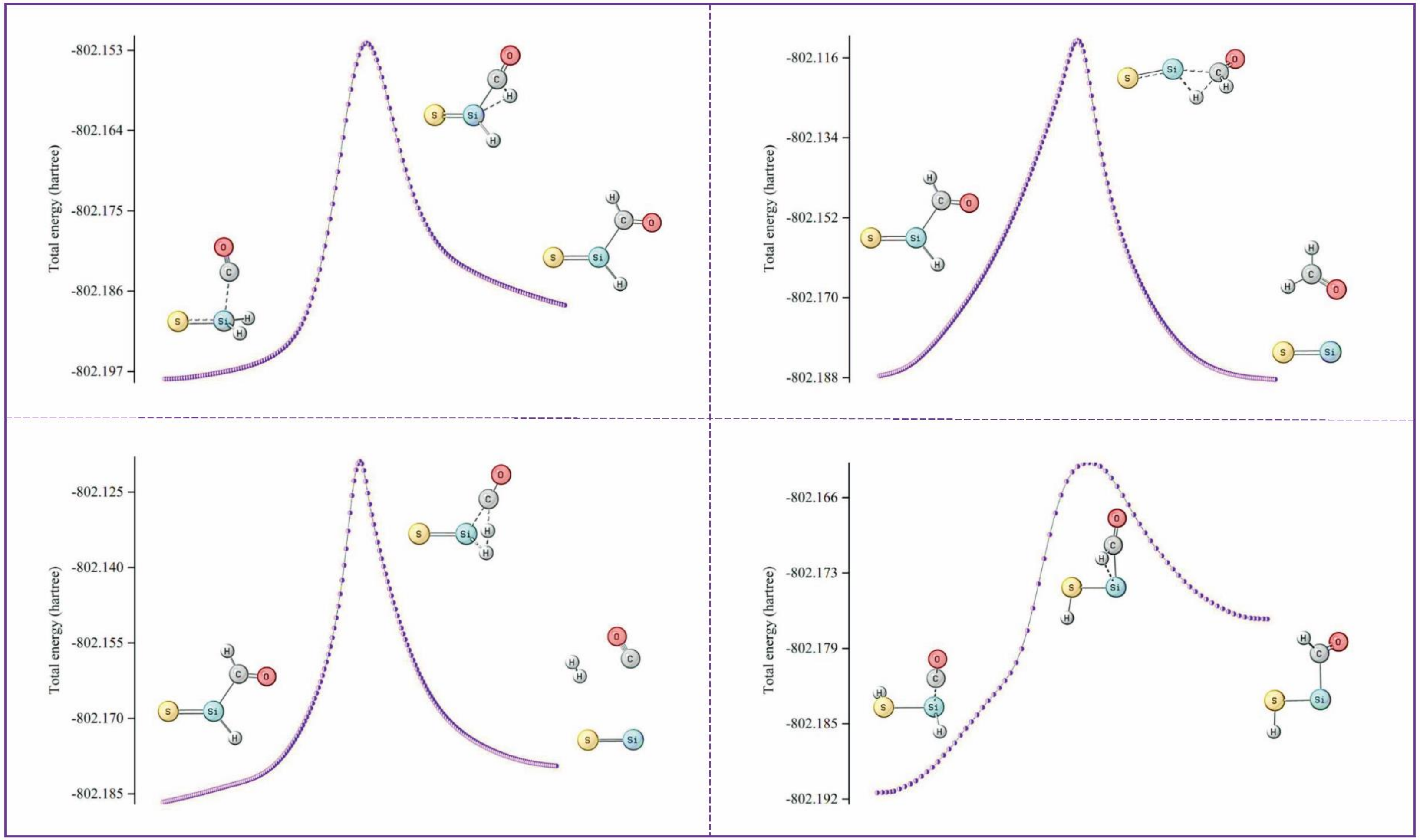

Total energy (hartree)
-802.153
-802.164
-802.175
-802.186
-802.197
Total energy (hartree)
-802.116
-802.134
-802.152
-802.170
-802.188
Total energy (hartree)
-802.125
-802.140
-802.155
-802.170
-802.185
Total energy (hartree)
-802.166
-802.173
-802.179
-802.185
-802.192

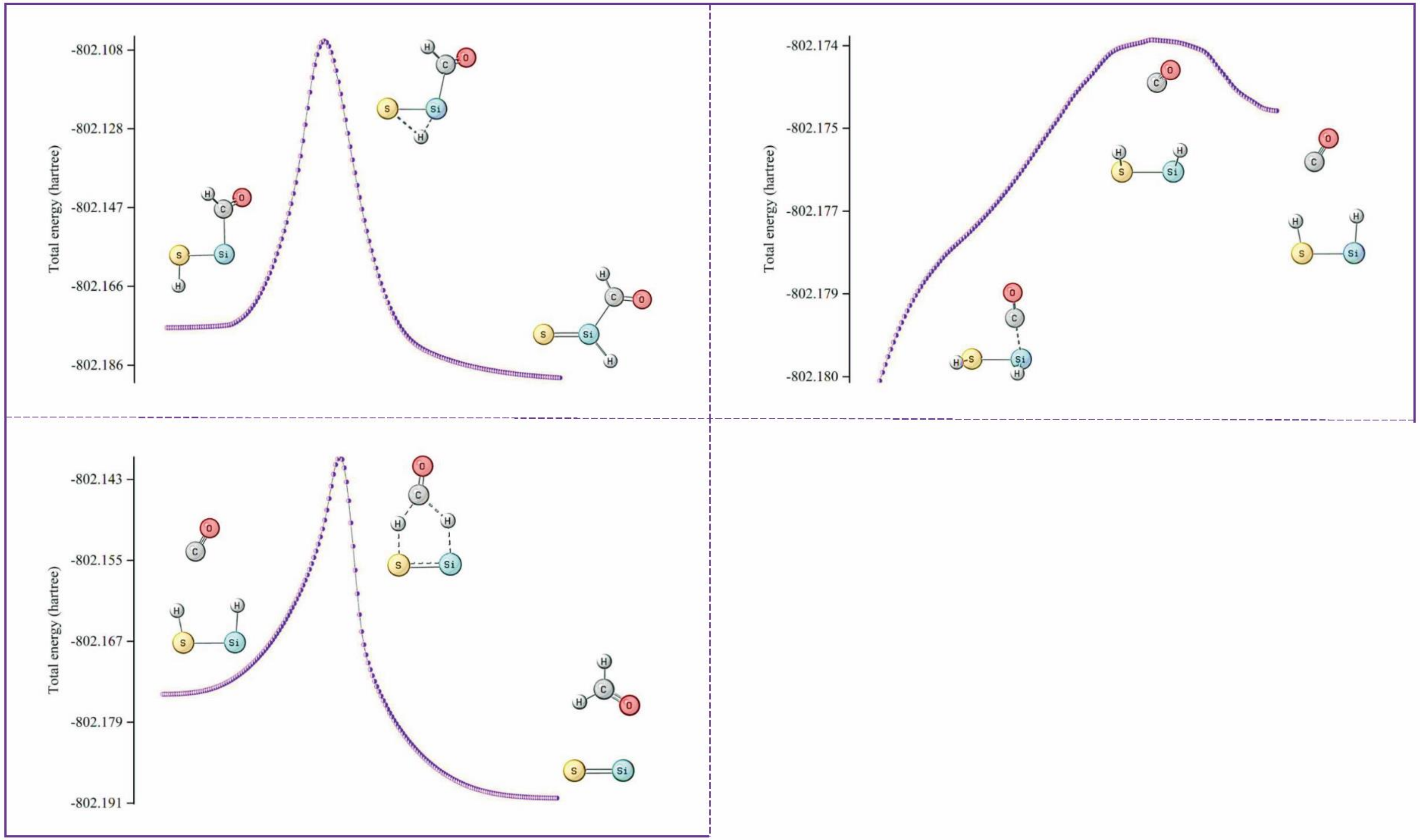

Figure S8. Intrinsic reaction coordinate calculation for the reactions of HSiSH with CO.

Reactions of HSiSH with CO: Part III Reaction pathways of HSiSH + CO

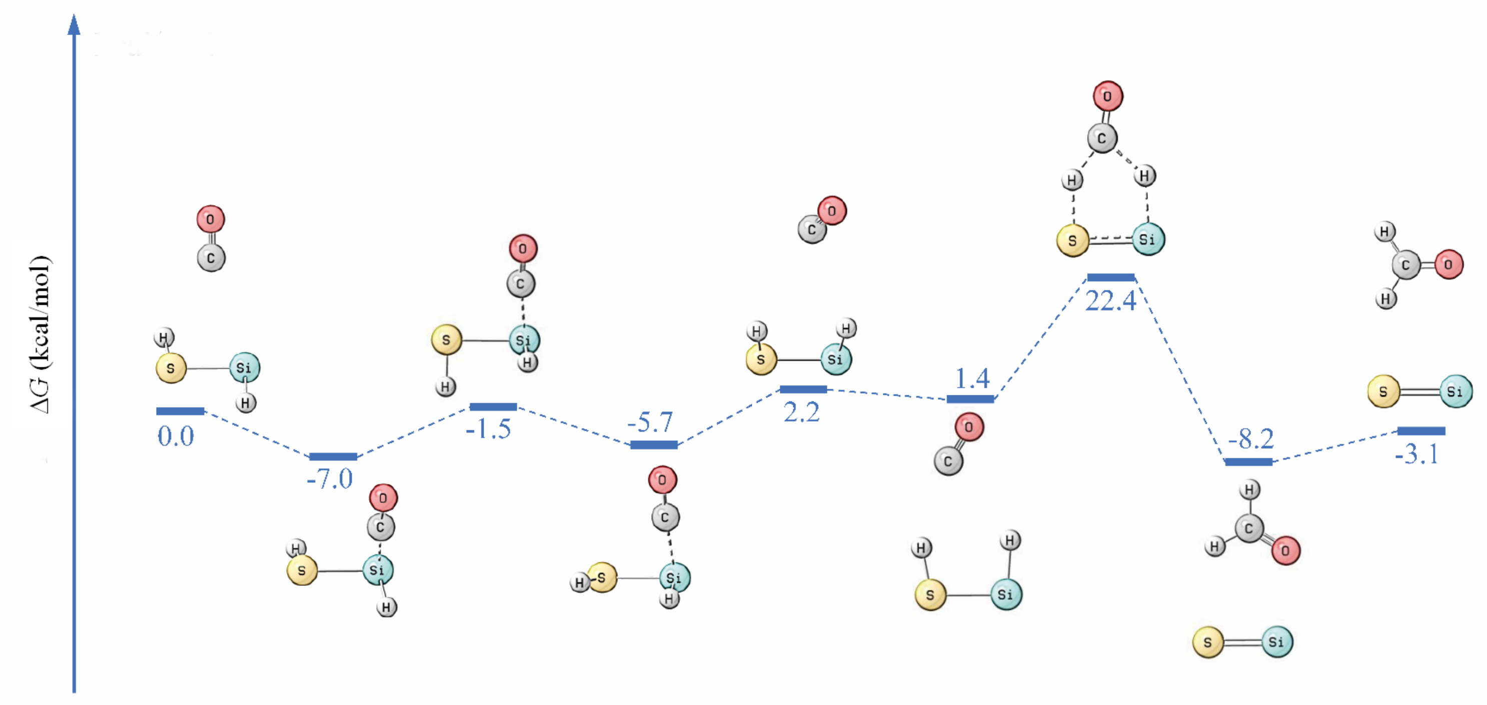

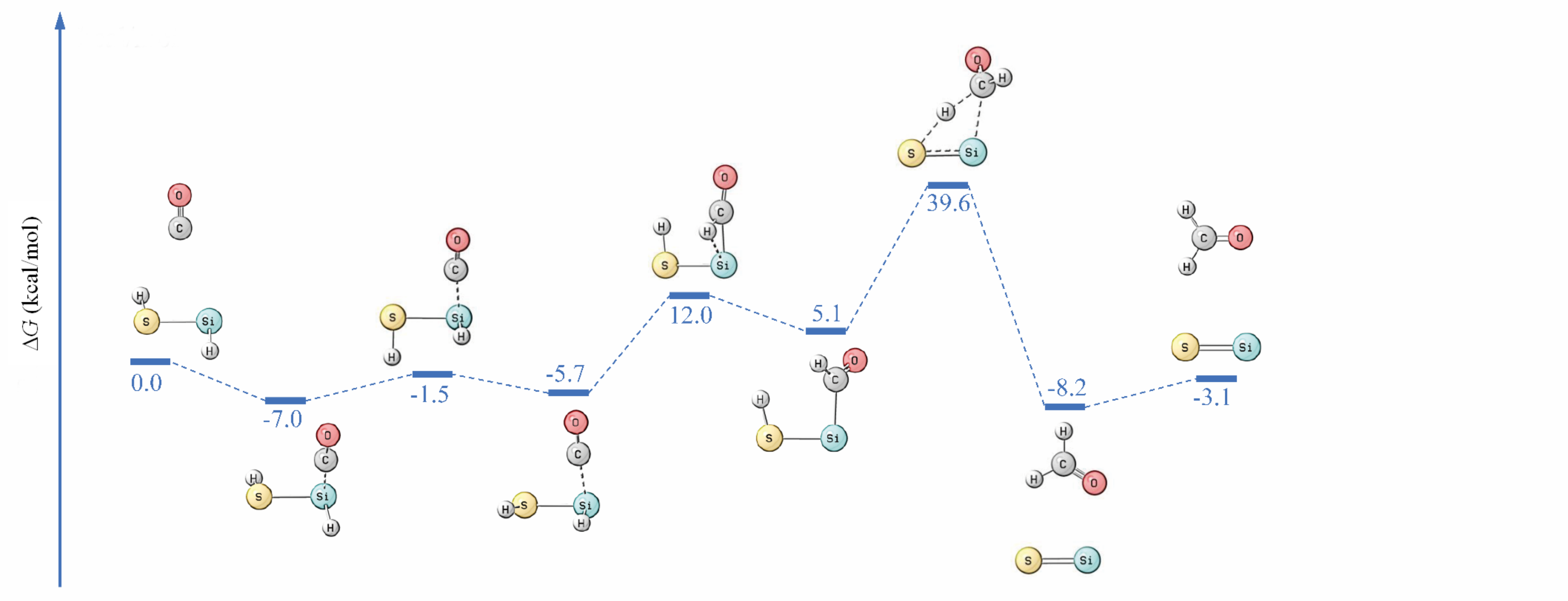
ΔG (kcal/mol)
0.0
-7.0
-1.5
-5.7
12.0
5.1
39.6
-8.2
-3.1

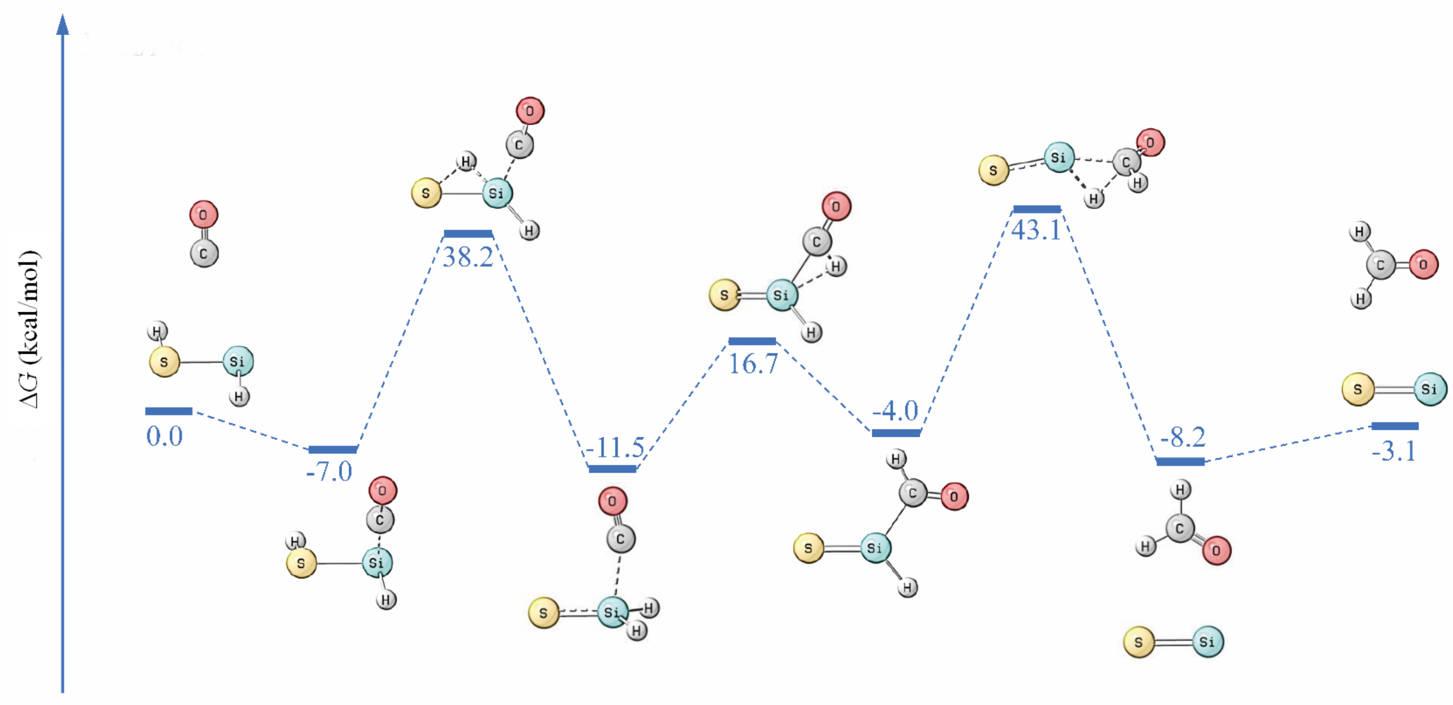

ΔG (kcal/mol)
0.0
-7.0
38.2
-11.5
16.7
-4.0
43.1
-8.2
-3.1

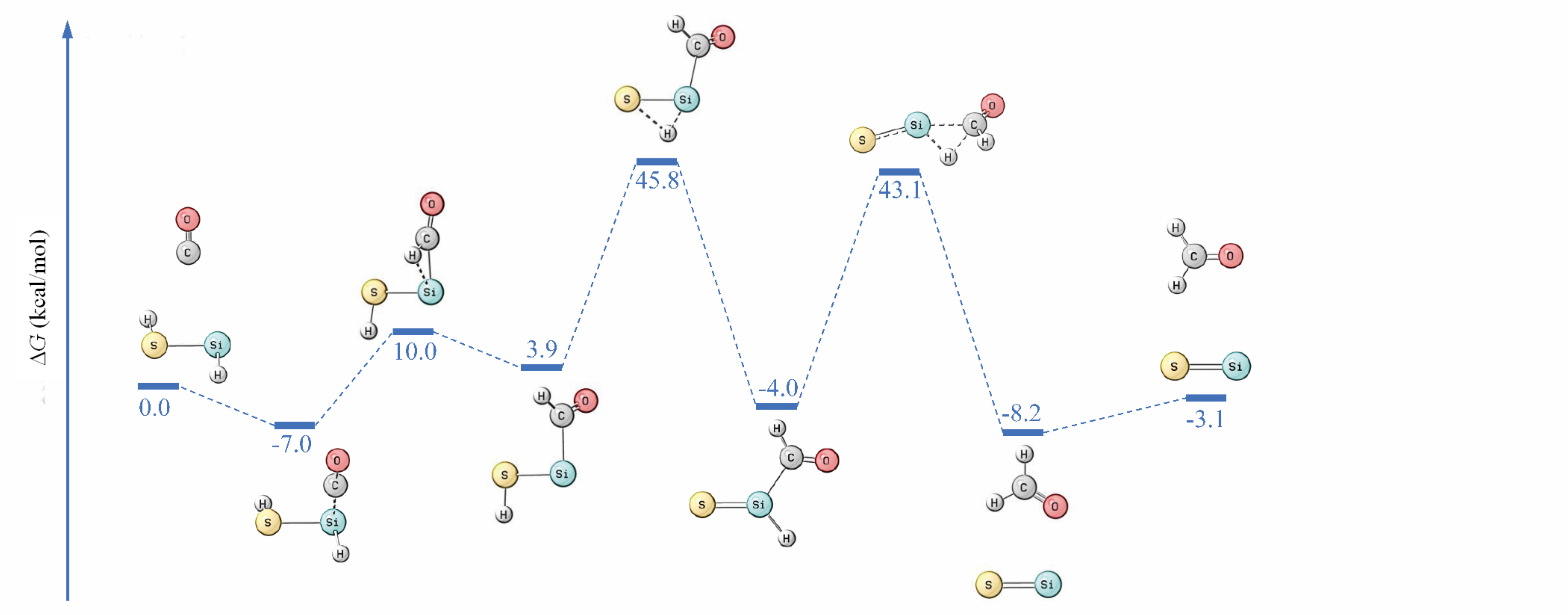
ΔG (kcal/mol)
0.0
-7.0
10.0
3.9
45.8
-4.0
43.1
-8.2
-3.1

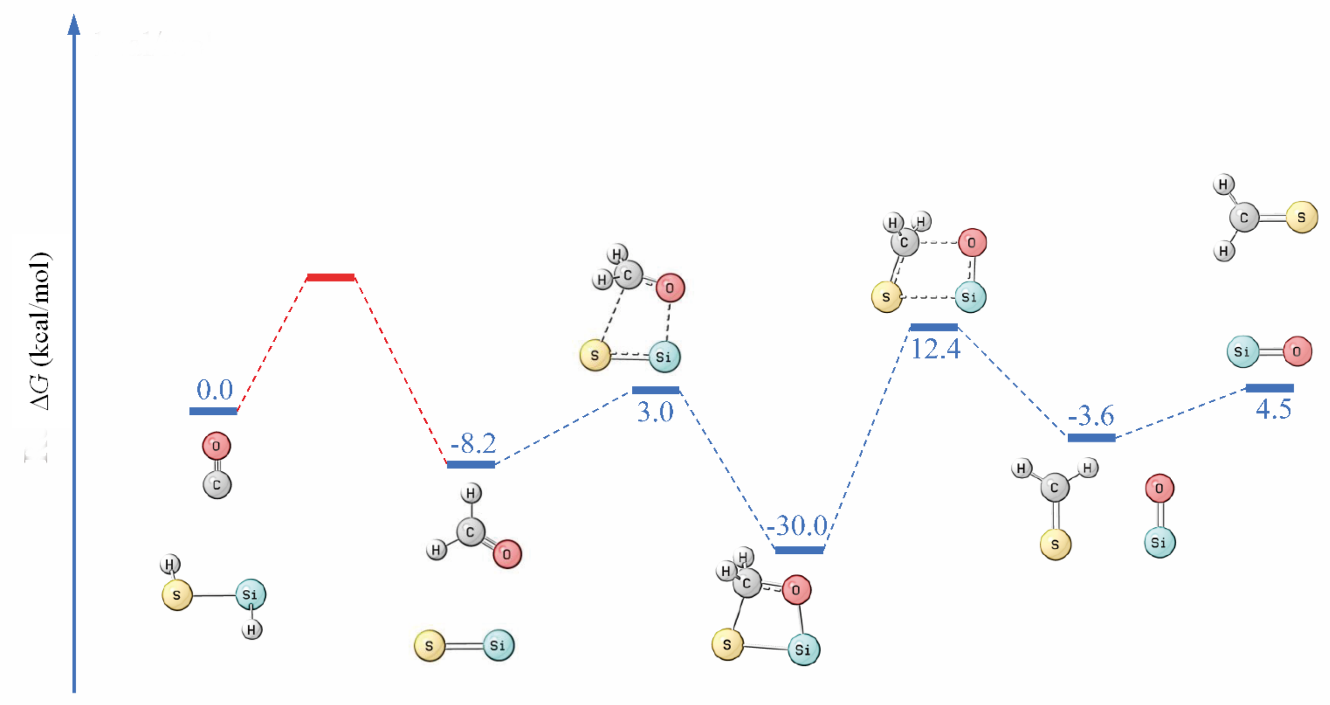

ΔG (kcal/mol)
0.0
-8.2
3.0
-30.0
12.4
-3.6
4.5

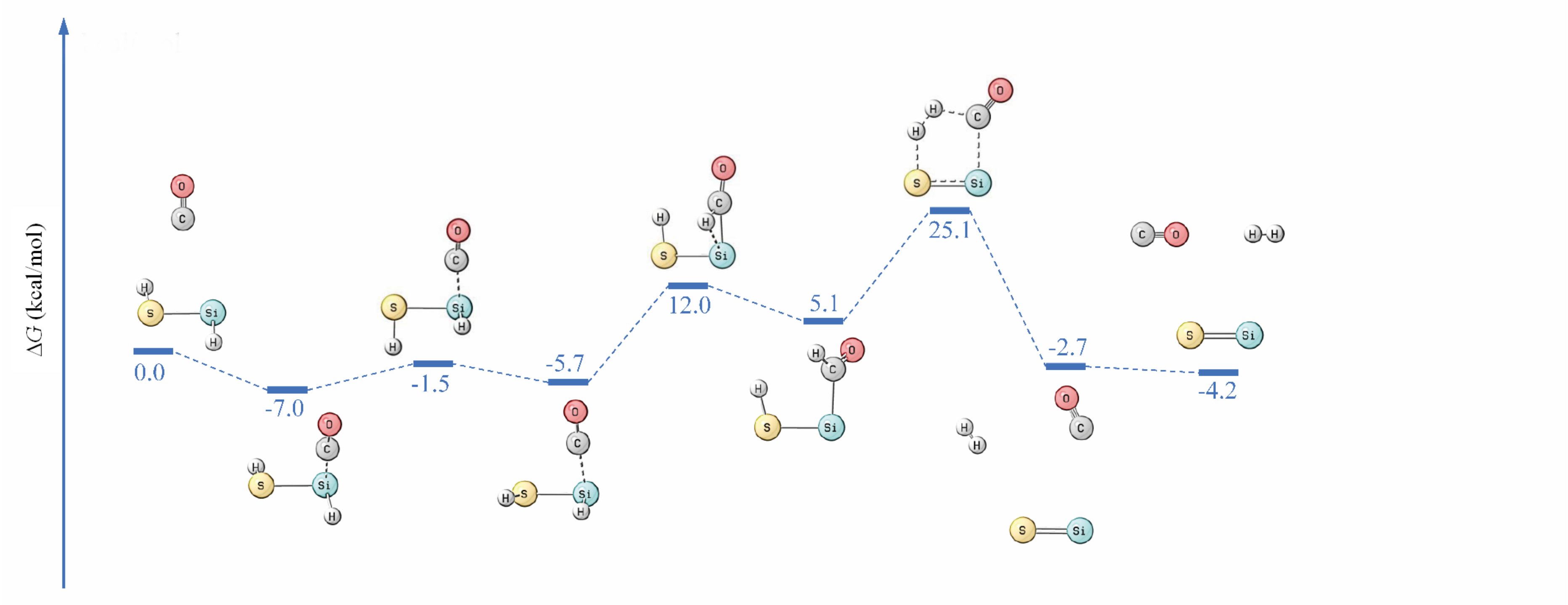
ΔG (kcal/mol)
0.0
-7.0
-1.5
-5.7
12.0
5.1
25.1
-2.7
-4.2
O
C
H
S
Si
H

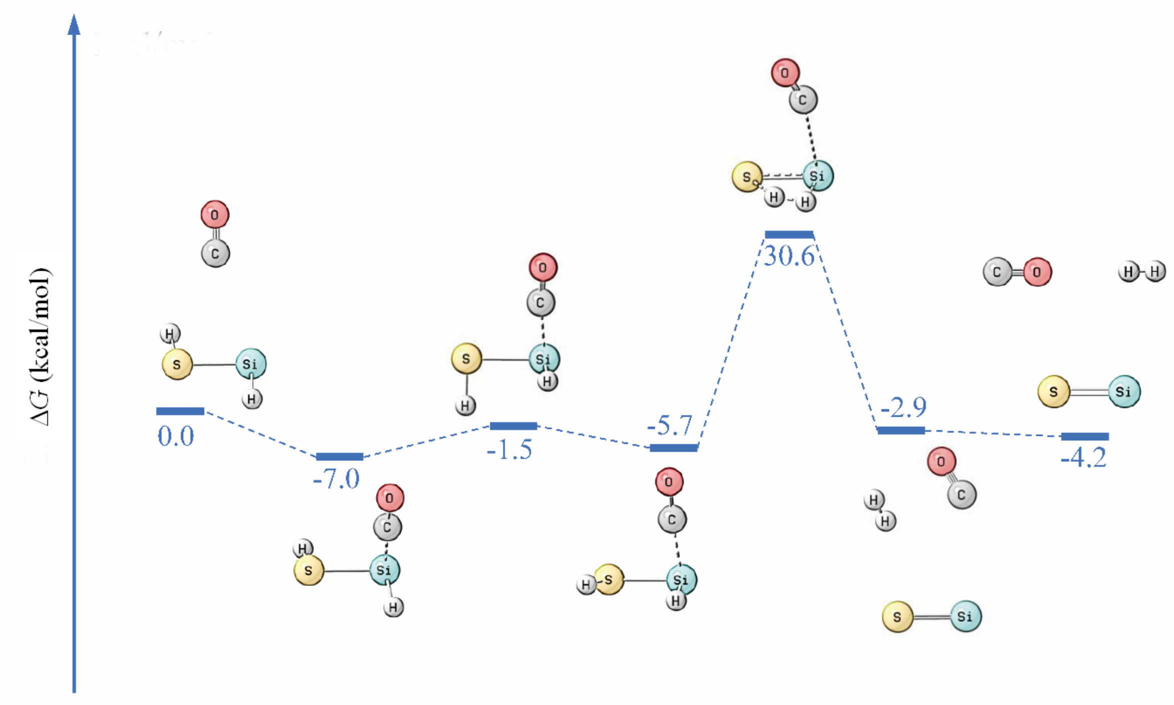

ΔG (kcal/mol)
0.0
-7.0
-1.5
-5.7
30.6
-2.9
-4.2
O
C
H
S
Si

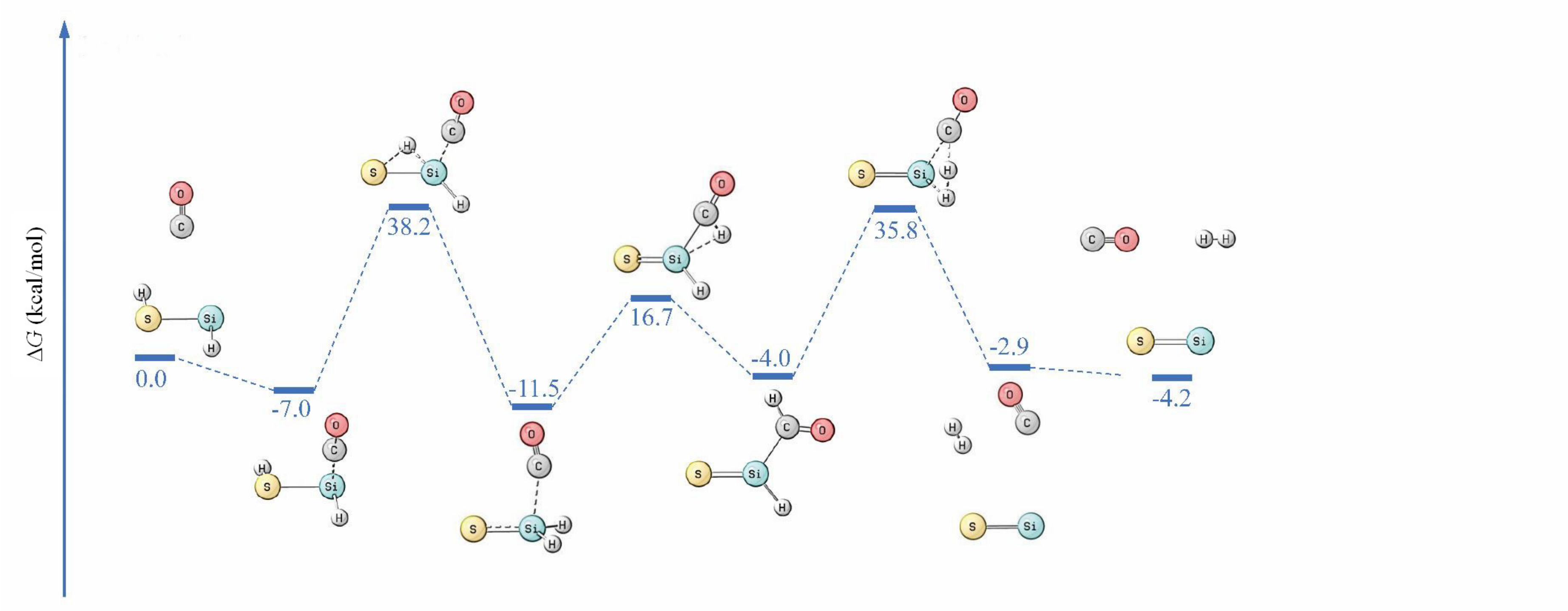
ΔG (kcal/mol)
0.0
-7.0
38.2
-11.5
16.7
-4.0
35.8
-2.9
-4.2

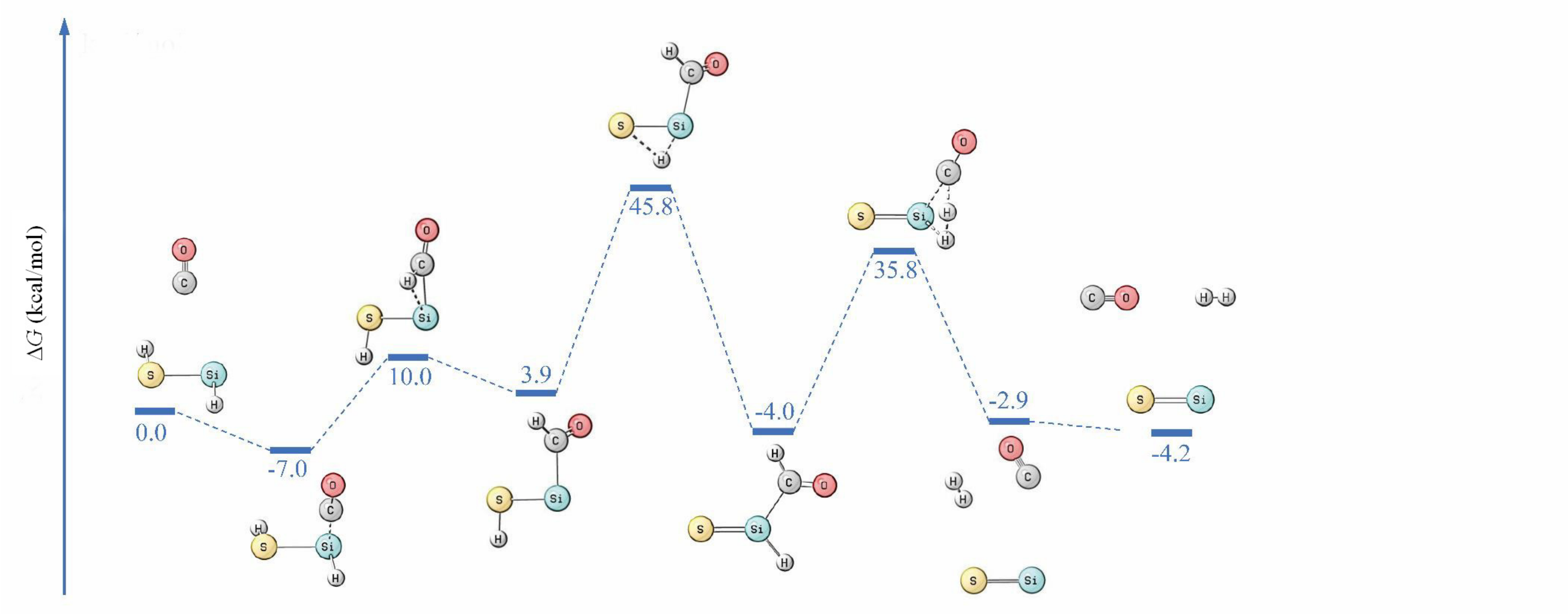

Figure S9. Reaction profiles for the HSiSH + CO.

Reactions of HSiSH with CO_2: Part I Cartesian coordinates of stationary points and transition states

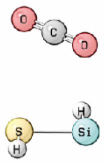

H	-0.59439600	0.91104100	1.50127200
H	-1.91012900	-1.07138700	-1.27022700
C	1.89045700	0.07574900	-0.06501600
O	2.32460900	-0.90861800	0.34398600
O	1.46340400	1.07271700	-0.47695600
Si	-1.30883400	1.08178600	0.17363100
S	-1.30116500	-1.04699700	-0.07550200

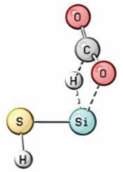

H	-0.56241600	0.16957100	-1.20272900
H	2.70267100	-0.03045000	0.55140700
C	-1.51361500	-0.12322500	0.02721400
O	-2.28486600	-0.92518000	-0.30770800
O	-1.05909400	0.65310400	0.85015900
Si	0.50440200	1.04311300	-0.37373900
S	1.66446800	-0.73917100	0.08629900

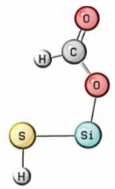

H	0.96172200	-1.30953900	0.34111900
H	-2.87635800	-0.29730200	-0.01948300
C	1.58461800	-0.44008100	0.09148700
O	2.75504600	-0.49491400	-0.08625900
O	0.88738500	0.71805200	0.01799600
Si	-0.73821000	1.13307000	-0.00123800
S	-1.64984900	-0.83754700	-0.01919500

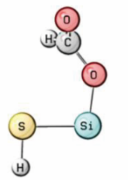

H	1.49804400	-0.58067700	1.41432200
II	-2.72929300	-0.57365500	-0.12391400
C	1.61079000	-0.22340300	0.38243100
O	0.86138100	0.87857900	0.13546300
O	2.32247100	-0.72315100	-0.42311700
Si	-0.77408200	1.13934800	-0.11510600
S	-1.44169700	-0.91872100	0.02048300

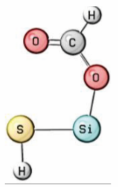

H	-2.89621800	0.13550500	0.00057000
H	2.65479300	-0.30017700	-0.00006600
C	-1.83973800	-0.15442200	0.00014600
O	-1.04858900	0.93478800	0.00034400
O	-1.45625800	-1.28134000	-0.00040400
Si	0.62827400	1.19505400	-0.00026500
S	1.40767400	-0.80419600	0.00017500

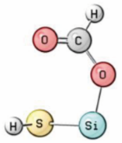

H	-2.38559300	-0.24615700	-1.22060400
H	1.35530600	-1.16578600	1.05371100
C	-1.74397100	-0.23180100	-0.33343800
O	-0.98509300	0.88246100	-0.28920000
O	-1.73614700	-1.07280300	0.50768500
Si	0.58004300	1.16542500	0.26774100
S	1.57146400	-0.74940500	-0.20804600

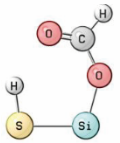

H	2.82691200	0.27808300	0.52047800
H	-0.42049600	-1.48785200	0.05752300
C	1.86657300	-0.07623300	0.12959400
O	0.98145700	0.92621400	0.06058700
O	1.66498600	-1.20623500	-0.19801000
Si	-0.69436200	1.17637700	-0.09324900
S	-1.56602000	-0.78512200	0.06558200

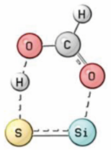

H	2.86568000	0.19405500	0.00007400
H	0.21572000	-1.20964700	0.00004100
C	1.79257700	-0.01432500	0.00006200
O	1.02399400	1.00258300	0.00004800
O	1.42235300	-1.19937300	0.00006000
Si	-0.81587900	1.14782900	-0.00003200
S	-1.37408300	-0.83710900	-0.00005600

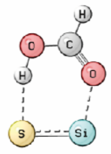

H	2.93145600	0.22664100	0.00217900
H	0.47894100	-1.24570500	-0.00135200
C	1.85389300	0.04779200	0.00047200
O	1.51318300	-1.17940000	-0.00060100
O	1.08202900	1.02072900	-0.00002100
Si	-0.91346900	1.10903900	-0.00024500
S	-1.40668100	-0.84530400	0.00029600

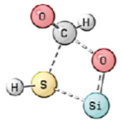

H	-1.04424700	-0.72860800	1.45242800
H	-0.72668100	1.33065800	-1.19517300
C	-1.05122100	-0.58671800	0.37507100
O	0.72275900	-1.25081800	0.23728000
O	-1.74722000	-1.02409700	-0.46182900
Si	1.64441300	-0.05775500	-0.17677400
S	-0.42174000	1.37038400	0.11022200

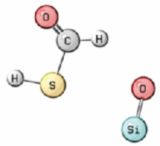

H	-0.63341200	-1.30171700	0.47148300
H	-2.05253300	1.53097300	-0.40051000
C	-1.42552300	-0.65060200	0.08064000
O	1.62703500	-0.89010100	0.52231800
O	-2.47294500	-1.01804700	-0.35262800
Si	2.34897900	0.12659500	-0.33519000
S	-0.92995900	1.07295100	0.17377000

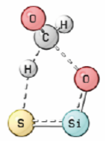

H	-1.78138300	-0.17666500	-1.47414900
H	-0.51493900	-0.88931500	-0.18739000
C	-1.61995600	-0.34239900	-0.39750000
O	-0.47920900	1.26785500	-0.07516900
O	-2.47058400	-0.44883400	0.41809100
Si	1.04300900	0.95261200	0.08517600
S	1.31326700	-1.04802300	0.00691900

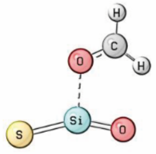

H	3.04858000	-1.43738500	-0.00009400
H	2.54604900	0.41507800	0.00068000
C	2.29275400	-0.64947900	0.00019200
O	0.73804400	1.63570200	0.00006200
O	1.11651500	-0.98142100	-0.00017600
Si	-0.21272700	0.44198700	-0.00018500
S	-1.95059100	-0.40643100	0.00011000

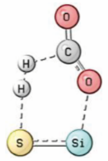

H	-0.94280900	-1.38107000	0.00024600
H	-0.08787300	-1.30973400	0.00025100
C	-1.73918900	-0.11757200	0.00001700
O	-0.97427900	0.82630500	-0.00001500
O	-2.84667900	-0.48926300	-0.00003500
Si	1.09180300	1.04882300	0.00001700
S	1.67176500	-0.87397600	-0.00002800

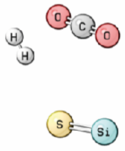

H	-0.99196000	-0.17342100	2.54719300
H	-1.65069100	-0.49635600	2.64513600
C	-1.87531900	0.19517800	-0.18527500
O	-2.28001400	-0.87940700	-0.28269100
O	-1.48230500	1.28113500	-0.09112300
Si	1.68577400	0.88431500	-0.00679800
S	1.27451800	-1.00597100	-0.06218700

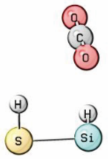

H	0.89237300	-1.20958000	0.87394900
H	-0.02216900	0.84951200	-1.32854400
C	-1.96891200	-0.12177900	0.14733200
O	-2.68805500	-0.56343700	-0.63114800
O	-1.25510700	0.32199700	0.95267500
Si	0.94330900	1.18841000	-0.19473200
S	1.83013900	-0.85096700	-0.01721000

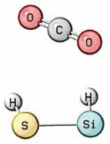

H	-0.70301200	-1.52550100	0.83066900
C	1.84967600	0.07053400	-0.05935000
O	1.51183000	1.09553500	-0.48210500
O	2.19104900	-0.94494000	0.36509600
Si	-1.29425200	1.09194400	0.19757500
S	-1.33414000	-1.01873100	-0.23831400
H	-0.55231200	0.91002100	1.50847200

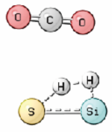

H	1.07826300	-0.13477700	1.21589300
C	-1.95613900	-0.19086700	-0.00552900
O	-1.48517400	-1.23899900	0.14974600
O	-2.43726900	0.84365000	-0.16125300
Si	1.59605100	-0.88143600	-0.25178700
S	1.15531900	1.12016100	0.06630700
H	1.20830300	-1.13971100	1.37343600

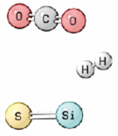

H	1.10396600	-0.00212400	2.61204500
C	1.87957900	0.11729500	-0.18927300
O	1.53801100	1.21813800	-0.30895500
O	2.23252900	-0.97323500	-0.07104700
Si	-1.63654100	0.90369400	-0.00622300
S	-1.30328700	-1.00219600	-0.05371900
H	1.21839800	0.72253800	2.51023500

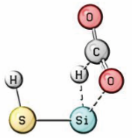

H	-0.55771100	0.22991500	-1.21893600
H	0.91188700	-1.65705700	-0.17555500
C	-1.50612300	-0.11697200	0.02538500
O	-2.24248300	-0.94540200	-0.32385500
O	-1.06490100	0.65756400	0.85280400
Si	0.50986500	1.06724400	-0.36839100
S	1.75022000	-0.65685800	0.13550400

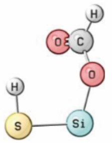

H	-2.64511200	-0.23138600	1.04126400
H	0.85212600	-1.30108500	0.84652800
C	-1.82939000	-0.22228000	0.30992000
O	-1.78008200	-0.93464300	-0.65144300
O	-0.90191600	0.68291200	0.61490500
Si	0.45931000	1.05102700	-0.36871700
S	1.73718600	-0.61464800	0.10669000

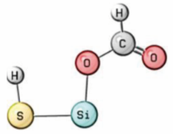

H	1.66120500	1.43796800	0.18270000
C	-1.99873700	0.56531600	-0.07448100
O	-0.65987900	0.60945300	-0.18558800
O	-2.61750300	-0.37270900	0.32555700
Si	0.29960500	-0.81600300	-0.17914200
S	2.17544700	0.19922100	0.12755600
H	-2.45133500	1.51268300	-0.38847500

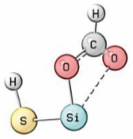

H	-2.64590600	-0.61246400	0.96298900
H	1.20670900	-1.21785300	0.91267700
C	-1.80417000	-0.33837900	0.31993100
O	-1.72491800	-0.62517900	-0.85353600
O	-0.85768300	0.35006300	0.91849300
Si	0.24152300	0.94490700	-0.32939300
S	1.84648100	-0.44794800	0.01853700

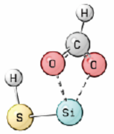

H	-2.58904700	-1.11504100	0.00525600
H	1.15537600	-1.52186300	0.00405000
C	-1.67263300	-0.52490800	0.00245200
O	-1.11704500	-0.14939200	-1.06594600
O	-1.11772200	-0.13838500	1.06724500
Si	0.05197800	0.96391100	-0.00462600
S	1.78874500	-0.33788700	0.00189700

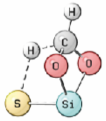

H	-2.11533800	-1.42080400	0.00001000
H	0.02320200	-1.40516900	0.00030400
C	-1.23225900	-0.78492300	0.00026200
O	-0.98533600	-0.01130500	-1.08568800
O	-0.98524700	-0.01064000	1.08579100
Si	-0.01913600	0.94030800	-0.00027900
S	1.59489100	-0.34082700	0.00007500

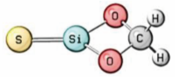

H	-2.62230300	0.00010000	-0.90499600
H	-2.62215700	0.00010000	0.90523400
C	-2.01903200	0.00010700	0.00006900
O	-1.09567300	-1.09655100	-0.00001900
O	-1.09550300	1.09657300	-0.00002500
Si	0.14015000	-0.00009600	-0.00001400
S	2.05787200	0.00002100	-0.00000700

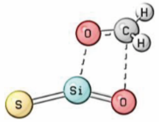

H	-2.38290100	0.06155900	1.01564900
H	-2.99459100	-0.68472900	-0.59331500
C	-2.17964200	-0.50398400	0.11089500
O	-1.08211300	-1.05704600	-0.09336800
Si	0.16803300	0.33153800	-0.01767300
S	2.00840300	-0.24628700	0.02647000
O	-0.92183100	1.42531400	-0.06460500

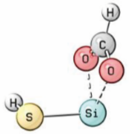

H	-2.48118600	-1.02239700	0.73073100
H	1.69130500	-1.22699400	-0.60820700
C	-1.62595200	-0.47001900	0.34010800
O	-1.05703100	-0.79782100	-0.74087600
O	-1.16313300	0.54996200	0.91525700
Si	0.01685400	0.78306700	-0.60239300
S	1.75443400	-0.24441000	0.30470500

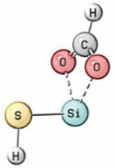

H	2.59955600	-1.10657300	0.01156100
H	-2.62288100	0.50960600	0.00054300
C	1.68340200	-0.51619100	0.00553700
O	1.12811300	-0.12428400	1.06759300
O	1.12665900	-0.14881600	-1.06459100
Si	-0.05957200	0.95124800	-0.01051700
S	-1.70507800	-0.46490900	0.00486800

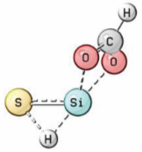

H	2.79698900	0.08506300	-0.86953900
H	-1.40638000	0.61579000	1.33641100
C	1.81795100	0.03717100	-0.39419800
O	1.28256100	-1.05553800	-0.05107700
O	1.14844300	1.07188400	-0.12048900
Si	-0.14588300	-0.03143900	0.68124000
S	-1.85649900	-0.03840600	-0.39165800

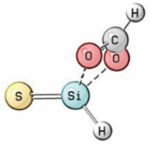

H	-2.80958900	0.00001900	-0.83565100
H	-0.15822700	-0.00004400	1.98249900
C	-1.81942500	0.00000900	-0.38499900
O	-1.19854400	-1.06387600	-0.10040300
O	-1.19853500	1.06388200	-0.10037600
Si	0.17895600	-0.00001100	0.55511700
S	1.90972600	0.00000500	-0.31264100

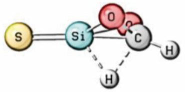

H	2.76642900	-0.00080800	0.70249300
H	0.81286200	-0.00055100	1.48708700
C	1.77733900	0.00026700	0.25674200
O	1.22491800	1.08857000	-0.30152100
O	1.22433400	-1.08840400	-0.30173000
Si	-0.10265600	-0.00037300	0.08320600
S	-2.02501000	0.00022900	-0.00430700

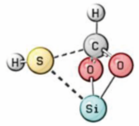

H	0.00279200	2.06846200	-0.00268600
H	1.78985500	-0.14415600	1.22470800
C	-0.29685500	1.02600000	0.00338100
O	-0.82946100	0.46105100	1.09239300
O	-0.86623500	0.46148200	-1.07015500
Si	-0.78897700	-0.98578500	0.00521100
S	1.53748300	-0.10372400	-0.09332300

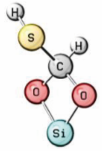

H	-0.43522700	0.15374800	1.67750700
H	-2.36300100	1.00853400	0.25514900
C	-0.24995400	0.05883400	0.60890200
O	0.58059200	1.10672100	0.14612400
O	0.55437000	-1.05949400	0.30442900
Si	1.74379700	-0.02717400	-0.33132100
S	-1.82468100	-0.09454100	-0.28450000

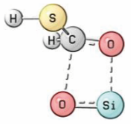

H	-0.36828700	0.07615800	1.57678500
H	-2.30858900	1.03256000	0.62492600
C	-0.46113100	-0.29303600	0.55902200
O	0.99805400	1.18567700	0.14887100
O	0.28076400	-1.20475500	0.09835300
Si	1.85609100	-0.06836400	-0.23300700
S	-1.92326000	0.10995100	-0.26697100

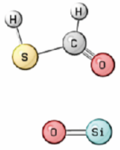

H	1.90034200	1.73041100	0.04948800
H	2.87169200	-0.29267000	0.03838900
C	1.07300500	1.01238100	0.01151200
O	-1.11336300	-1.20396500	0.00678500
O	-0.06939500	1.42469800	-0.01393700
Si	-1.92804000	0.08679000	0.00139300
S	1.57778500	-0.65581000	-0.00745200

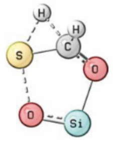

H	-1.13974300	1.51691600	1.17732600
H	-2.14393700	0.84707400	-0.28551600
C	-0.76324100	0.99997200	0.29574400
O	0.36903500	1.37642800	-0.19634200
O	0.52741300	-1.23836700	0.23846400
Si	1.57934600	-0.08297000	-0.05783900
S	-1.33870600	-0.51917100	-0.13709400

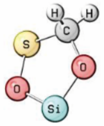

H	-0.75271700	1.55792000	1.21513900
H	-1.13482900	1.96304000	-0.48416400
C	-0.63487800	1.25627700	0.17449400
O	0.73615000	1.16810700	-0.13877700
O	0.08768400	-1.19780800	0.18687500
Si	1.49241600	-0.31504100	-0.03075600
S	-1.36173000	-0.40065300	-0.10825900

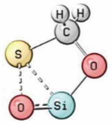

H	-1.40673600	0.73551800	1.42148700
H	-2.05414500	1.08326000	-0.23086300
C	-1.20901900	0.72408000	0.35300900
O	-0.06423400	1.51423200	0.02690500
O	1.35320100	-0.80718600	0.64226000
Si	1.00707200	0.30331900	-0.39364500
S	-0.85598400	-1.00413100	-0.19693500

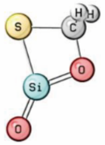

H	1.93691900	1.07615500	-0.89962100
H	1.93698900	1.07663600	0.89990500
C	1.39438300	0.80323200	0.00023600
O	0.09426200	1.39566700	-0.00001700
O	-2.27623100	-0.26294800	0.00042800
Si	-0.79385100	0.01443100	-0.00040600
S	1.02059100	-1.01474700	0.00004300

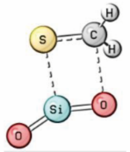

H	2.58666500	0.89270800	-0.46632800
H	1.48495600	1.21681000	0.97767600
C	1.75127300	0.57758600	0.15306500
O	-0.31222400	1.59735700	-0.14362500
O	-2.10745900	-0.74378300	0.06207000
Si	-0.91236200	0.18232800	0.01114600
S	1.09695400	-0.93476400	-0.05833300

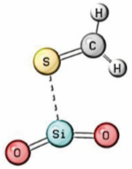

H	3.10561100	0.49425500	0.00007800
H	1.50715800	1.50784700	-0.00000200
C	2.02267900	0.54857500	0.00010000
O	-0.68369100	1.66412500	-0.00002300
Si	-1.01025600	0.17568200	-0.00002400
O	-1.93816200	-1.02501200	0.00007000
S	1.14809800	-0.80412500	-0.00004500

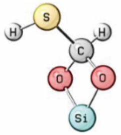

H	-0.40083700	-0.19775500	1.68643800
H	-1.67378600	1.17901700	-0.72036400
C	-0.24342000	-0.08377200	0.61536800
O	0.53460400	1.05394400	0.31823800
O	0.60165000	-1.10889400	0.12611400
Si	1.74199200	0.04998400	-0.33544100
S	-1.87142400	-0.04617500	-0.21980700

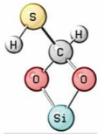

H	-0.43228200	0.00043900	1.66261800
H	-1.42944300	-0.00113500	-1.42824400
C	-0.26153400	0.00024400	0.58584600
O	0.56581500	1.08465000	0.19752600
O	0.56572200	-1.08447600	0.19799700
Si	1.76725400	-0.00013100	-0.29845900
S	-1.89768200	-0.00002000	-0.17095100

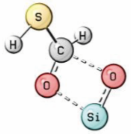

H	-0.35411300	-0.10011700	1.58783800
H	-1.76253100	0.49019100	-1.27755500
C	-0.46836900	0.26713800	0.57043400
O	0.26171200	1.18307700	0.09456800
O	1.02050600	-1.18239400	0.12829800
Si	1.86122900	0.09033800	-0.22705700
S	-1.96175600	-0.20394400	-0.14606300

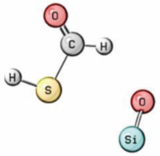

H	-0.63341200	-1.30171700	0.47148300
H	-2.05253300	1.53097300	-0.40051000
C	-1.42552300	-0.65060200	0.08064000
O	1.62703500	-0.89010100	0.52231800
O	-2.47294500	-1.01804700	-0.35262800
Si	2.34897900	0.12659500	-0.33519000
S	-0.92995900	1.07295100	0.17377000

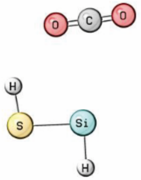

H	-0.98284400	-1.48722300	-0.00002500
H	-2.21535200	1.95547200	0.00009400
C	2.21450800	-0.27406200	-0.00004400
O	1.53035600	-1.20788000	0.00003600
O	2.91219200	0.64424900	-0.00011900
Si	-0.93123500	1.15440800	0.00014300
S	-2.03699600	-0.65478400	-0.00007100

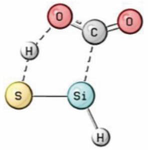

H	-0.58772400	-1.18922200	0.00027400
H	-0.76860000	2.42103500	0.00042700
C	1.38279600	-0.20090300	0.00008700
O	0.89526100	-1.33984700	0.00030600
O	2.45660400	0.32190800	-0.00037400
Si	-0.33102700	1.00122600	0.00017600
S	-1.82006200	-0.36875400	-0.00019700

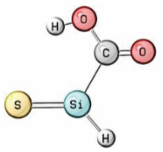

H	0.43562300	1.72946400	0.00047000
C	1.33089600	0.05306300	0.00000600
O	1.34411100	1.39086100	0.00017300
O	2.33180300	-0.60138900	-0.00032400
Si	-0.39808900	-0.78849200	0.00018800
S	-2.00069800	0.30795600	-0.00016000
H	-0.24389000	-2.25202300	0.00063900

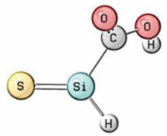

H	1.94786800	-1.51320600	-0.65860300
C	1.30603400	0.18194200	0.01091700
O	2.26972700	-0.62147200	-0.49041800
O	1.54081500	1.32548500	0.25782800
Si	-0.45368000	-0.50125300	0.41819500
S	-2.10038300	0.21493400	-0.29766800
H	-0.31076000	-1.63196400	1.36178400

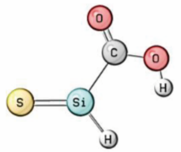

H	-2.16660700	-1.47783200	0.00004000
C	-1.29787000	0.24636200	-0.00001400
O	-2.39792500	-0.54235000	0.00004300
O	-1.39316200	1.43209900	-0.00001000
Si	0.42344500	-0.64045400	-0.00005900
S	2.13610100	0.24700900	0.00003000
H	0.17668500	-2.10411500	0.00012800

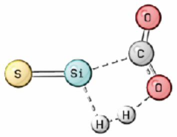

H	0.87355600	1.71101300	-0.00003500
C	1.66797700	-0.16461900	0.00003100
O	2.05536800	1.01157600	0.00016700
O	2.08284100	-1.27507300	0.00006500
Si	-0.45159400	0.13664800	-0.00035400
S	-2.35323100	-0.15017100	0.00016800
H	-0.01307700	1.87434400	0.00026100

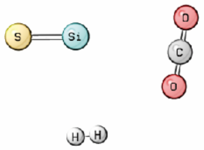

H	-0.20734300	2.69057900	0.85806900
C	-2.64582100	-0.12101700	0.12614400
O	-2.49346900	1.02361900	0.07201600
O	-2.79798500	-1.26452100	0.18019900
Si	0.87539600	-0.39548200	-0.01114400
S	2.79720800	-0.25602000	-0.09504400
H	0.51642700	2.80024600	0.97029500

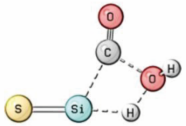

H	2.79960100	-0.97803600	0.54949300
C	1.20810900	0.49893800	-0.03481600
O	2.07054300	-0.94120800	-0.08345700
O	1.82992700	1.46738500	0.01403000
Si	-0.49667800	-0.65132400	0.00309500
S	-2.20210200	0.27899800	0.00493700
H	0.93510500	-1.57044500	0.09250500

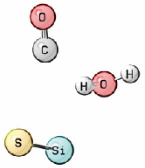

H	0.17002800	1.44754600	-1.12334300
C	2.12958600	-0.74402100	0.52446600
O	0.60151200	1.78205200	-0.32947100
O	2.90979100	-0.69642700	-0.27952300
Si	-1.11824600	0.11212400	1.03339200
S	-1.64170700	-0.61984200	-0.69366000
H	0.88478700	2.67931400	-0.52043000

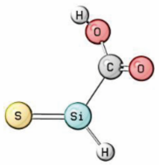

H	1.42731700	1.87498500	0.52310200
C	1.33602800	0.00090100	0.00013800
O	1.52269900	1.32628200	-0.26425500
O	2.22051600	-0.73257000	0.31910000
Si	-0.44611300	-0.70048800	-0.19129900
S	-2.05117600	0.33195800	0.13883200
H	-0.32481500	-2.13457800	-0.50581300

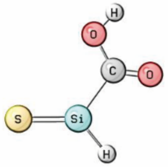

H	2.37361000	1.56953800	-0.13515500
C	1.33670400	0.01474900	0.07482500
O	1.43348700	1.33849800	-0.06789800
O	2.27865600	-0.72929600	0.11139500
Si	-0.45026400	-0.68721400	0.22247000
S	-2.09683800	0.32127900	0.11328300
H	-0.26793800	-2.13454300	0.42165600

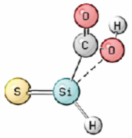

H	-1.56215800	0.94115600	-1.49142800
C	-1.34940300	-0.27199800	0.18876200
O	-1.22685500	0.98612000	-0.58183100
O	-2.24297200	-1.01243000	0.05387800
Si	0.38948800	0.38162300	0.53675300
S	1.98193500	-0.36792800	-0.27947000
H	0.25338500	1.44545400	1.53946400

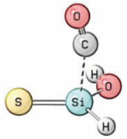

H	0.85939300	1.75504300	-1.33955800
C	-1.68758300	0.03593000	0.11994000
O	0.56026000	1.85027000	-0.43116100
O	-2.63038900	-0.48838300	-0.16982000
Si	0.51274600	0.47336700	0.44728700
S	1.14620300	-1.27580900	-0.16642200
H	0.30945600	0.92007400	1.82849200

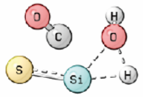

H	1.22513400	1.91597800	-1.38124900
C	-2.34946500	0.24985200	0.30881900
O	0.79769300	1.78555000	-0.51589300
O	-3.22229700	-0.26021000	-0.17462100
Si	0.98428400	0.36183600	0.56270800
S	1.07687500	-1.41656500	-0.21956100
H	1.25850100	1.98152600	0.68751700

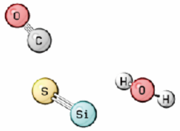

H	2.45560200	-0.43393300	1.22320100
C	-2.67265400	-0.77906500	-0.24203000
O	2.52306400	-1.18758100	0.62612100
O	-3.41963300	-0.37014600	0.48707400
Si	0.74913800	-0.12703700	-0.99741000
S	0.42723600	1.30333300	0.28872500
H	3.42915500	-1.50467300	0.66756800

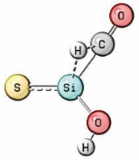

H	-0.61980100	2.47399600	-0.15021000
C	1.51651900	-0.37740500	0.02024400
O	0.11630500	1.89347000	0.06182600
O	2.56029000	-0.87778600	0.07014800
Si	-0.16417200	0.29519000	-0.00200100
S	-1.78764000	-0.76974200	0.01718000
H	1.00857800	-0.15181400	-1.27389900

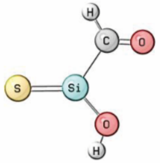

C	-1.43839500	-0.91834500	0.00004000
O	-2.54034100	-0.44836200	-0.00000200
Si	0.13813600	0.18814600	-0.00004800
S	1.95734000	-0.47799400	0.00001500
H	-1.27726300	-2.01339500	-0.00001900
O	-0.40759400	1.71264400	0.00002900
H	0.23977500	2.42307800	0.00001000

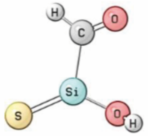

H	-1.21443800	-2.03297300	-0.22339000
H	-0.61753600	2.32176300	0.61194600
C	-1.39243900	-0.95108300	-0.07337100
O	-0.39516100	1.73337600	-0.10899500
O	-2.49801000	-0.51786900	0.09835900
Si	0.14337300	0.21335200	-0.03334100
S	1.95779700	-0.45583000	0.03772100

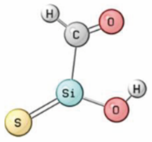

H	1.26668800	-2.08598200	0.00000500
H	1.46388800	1.66275000	0.00029100
C	1.37318900	-0.98708700	0.00016300
O	0.49803900	1.65493200	0.00024500
O	2.46076800	-0.46837300	-0.00032000
Si	-0.16398800	0.17713500	0.00001300
S	-2.02152100	-0.35166300	-0.00005300

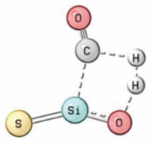

H	2.37069100	0.97162300	0.00032900
H	1.65067200	1.53670200	0.00030500
C	1.51699400	-0.44964700	0.00014100
O	2.17938600	-1.36894400	-0.00010700
Si	-0.28934300	0.48643000	-0.00002100
O	0.33809000	1.90621800	-0.00008000
S	-1.82577100	-0.68241600	0.00001900

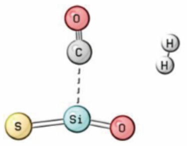

H	3.46250200	1.38155600	0.23161600
H	2.79876200	1.70519200	0.14878700
C	1.35962800	-0.71791600	-0.01670500
O	2.17169200	-1.48073800	-0.02303400
Si	-0.39679900	0.61938200	-0.01092300
O	0.34103800	1.94199900	-0.03715000
S	-1.81035500	-0.69629300	0.02213900

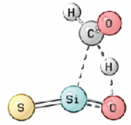

H	1.61301600	-1.04512100	1.47879000
H	1.30012700	0.79580600	0.32971100
C	1.57951900	-0.57267100	0.47949500
O	0.49254200	1.85561000	-0.06593600
O	2.18478100	-1.02595800	-0.45180000
Si	-0.32700000	0.53839700	-0.00927800
S	-1.82692800	-0.65558900	-0.02585600

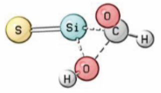

H	2.24733400	0.33299000	1.22693300
H	0.74498000	1.33150300	-1.11041500
C	1.83722600	-0.08520600	0.31165400
O	0.75452600	1.13797300	-0.15877100
O	2.33654900	-0.72614900	-0.54067300
Si	-0.33700300	-0.14191100	0.53381000
S	-2.12664000	-0.15381800	-0.24151400

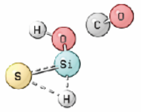

Si	-0.22039300	0.46183500	-0.44501600
S	-1.49847000	-1.01355400	0.18456300
H	-0.54772900	-0.77718300	-1.35743300
C	1.61213500	-0.22709400	0.20678400
O	2.60853800	-0.73089200	0.07740600
O	-0.25500600	1.95664300	0.22924500
H	-0.89231700	2.08492000	0.94073400

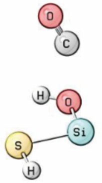

H	1.49887000	-1.85540700	0.89701700
H	0.60691800	1.22626700	-1.56419400
C	-2.29434600	0.05616600	0.32152600
O	0.63007300	1.60998400	-0.68425000
O	-3.23380600	-0.34921800	-0.13574500
Si	0.94716900	0.72039700	0.67286800
S	1.20186100	-1.24247200	-0.25763500

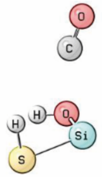

H	0.48350300	-1.64808300	-0.42829200
H	0.84535400	1.30337200	-1.46714300
C	-2.23065800	-0.46715700	-0.04726600
O	0.48397900	1.64883700	-0.64372600
O	-3.34508800	-0.35865000	-0.04190500
Si	0.59648700	0.71159300	0.70735900
S	1.66207200	-1.07100900	-0.13993400

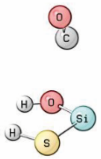

H	-1.00409800	1.09076400	-1.40711300
H	-0.95328400	-1.23438500	-1.55562600
C	2.28515000	-0.22895300	0.10414400
O	-0.91277000	-1.60291700	-0.67046100
O	3.28550500	0.24650100	-0.06618200
Si	-0.93872200	-0.67752000	0.69743600
S	-1.09958100	1.36587200	-0.09581800

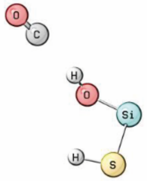

H	1.19074900	-1.45105100	-0.74356800
H	-1.00744300	0.59587200	-0.31315700
C	-3.09683500	-0.11422200	0.04637000
O	-0.12293300	0.84449500	-0.59488300
O	-4.14146100	-0.50090000	0.14883000
Si	1.26186300	1.00803500	0.28770800
S	2.17792300	-0.95754600	0.01993800

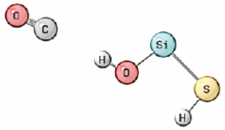

H	2.23345000	-1.44466200	0.00010200
H	-1.08237800	-0.45616500	-0.00346900
C	-3.31810600	-0.11562800	0.00076800
O	-0.13043000	-0.60501500	-0.00235100
O	-4.43097900	-0.00176200	0.00104900
Si	0.80875200	0.76394600	-0.00076900
S	2.74539400	-0.20290200	0.00124600

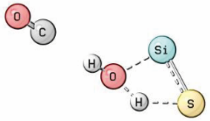

H	0.80748700	-0.84279100	-0.50582100
H	-0.97958700	-0.08591900	-0.52692100
C	-3.14009700	-0.15899400	0.02570600
O	-0.04815600	-0.01214000	-0.78337400
O	-4.23048500	-0.18307700	0.27227000
Si	1.15642900	1.02131300	0.13638400
S	2.31573800	-0.67837300	0.19112200

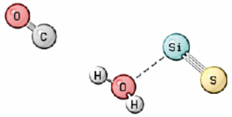

H	0.21271600	-1.50694700	-0.29234500
H	-1.15958600	-0.74478900	-0.45668400
C	-3.22445600	-0.14216900	0.12190600
O	-0.23961500	-0.78687600	-0.75508300
O	-4.25105100	0.21261300	0.38651500
Si	1.14188100	0.88100400	-0.31898300
S	2.51453700	-0.28970000	0.46449400

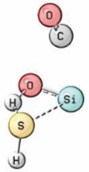

H	-2.01281000	1.76289500	-0.15926400
H	-0.95022200	0.32448300	0.91159900
C	2.12060200	-0.36486800	-0.20769700
O	-1.15323400	-1.07227300	0.98172000
O	3.11740700	-0.00081600	0.14947400
Si	-1.01487100	-1.09021400	-0.57316000
S	-0.70411100	1.49684700	-0.03321600

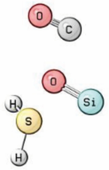

H	0.53081300	1.01798600	-1.02276400
H	0.41058200	2.81360400	-0.28709000
C	1.04474700	-1.72616500	0.58047800
O	-1.50780000	-0.51743700	-0.95184800
O	1.81917600	-1.83212400	-0.22304000
Si	-1.85387100	-0.09518600	0.46182100
S	1.01583200	1.66590600	0.04753800

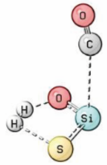

H	1.23254800	0.19527800	-1.83079300
H	0.86887400	0.97103500	-1.55939200
C	-2.18282300	-0.25228400	0.23000900
O	0.40594000	1.65362100	-0.43120100
O	-3.19868200	-0.43292400	-0.20304000
Si	0.65192700	0.54621700	0.65444300
S	1.51315500	-1.06657600	-0.12988400

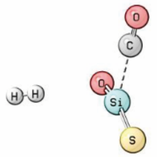

H	1.26328500	4.43758100	-1.32905700
H	1.04707200	3.92957500	-0.83493500
C	-1.67770400	-0.27417200	-0.04117300
O	0.14944400	1.80592400	0.38540500
O	-2.74548100	-0.57724400	-0.13720600
Si	0.45667100	0.34108700	0.14833400
S	1.38317300	-1.33292400	-0.10320200

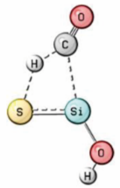

H	-1.04877100	1.03844900	0.00085700
H	2.68133400	-1.00562300	0.00008300
C	-1.81660400	-0.02474500	0.00059900
O	1.82995500	-1.45200500	0.00001900
O	-2.82885500	-0.54697900	-0.00035800
Si	0.46504600	-0.54403900	-0.00002200
S	0.67172600	1.48275400	-0.00009400

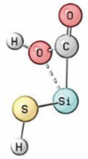

H	2.71786800	0.20386200	-0.18543400
H	-1.17187700	0.31182100	-1.72928000
C	-1.18889500	-0.22440600	0.28666800
O	-1.17682000	0.76892800	-0.87531500
O	-1.96006300	-1.09971300	0.31873300
Si	0.31041500	0.93683800	0.55459100
S	1.64604000	-0.60241900	-0.19480700

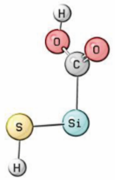

H	2.85594800	0.02545600	-0.01277800
H	-2.50730200	-1.16211600	-0.43161200
C	-1.15887600	0.06030000	0.06764800
O	-1.68167200	-0.81650200	-0.80864300
O	-1.68569500	0.32041000	1.11859100
Si	0.46631700	1.00762700	-0.45308400
S	1.68844400	-0.58519900	0.24388100

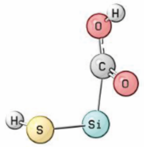

H	-1.86171800	-1.03839500	1.08078700
H	2.69222000	-0.97801900	-0.43580500
C	1.19376200	-0.04325600	0.17050100
O	2.15236000	-0.89780100	0.37107700
O	1.05360200	0.56618500	-0.91066700
Si	-0.36289900	1.02938500	0.40073700
S	-1.78501200	-0.59265700	-0.18509900

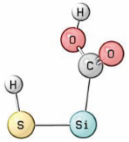

H	-0.93823700	-1.45505200	-0.58367800
H	2.39608800	-1.31896700	0.31993000
C	1.16292500	0.06662600	-0.02775500
O	1.56542200	-0.99100700	0.70179700
O	1.77473500	0.45996500	-0.98813100
Si	-0.45817000	1.01986500	0.48080100
S	-1.79639200	-0.47847000	-0.25064100

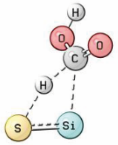

H	-0.15030200	-0.58225500	-0.14940000
H	2.77750700	-0.57801600	0.81152700
C	1.21816400	-0.09698200	-0.14378200
O	1.82655100	-0.48754300	0.98809300
O	1.78407600	0.09416300	-1.18149400
Si	-0.66379800	1.02938600	0.26744200
S	-1.84550200	-0.59513800	-0.12477600

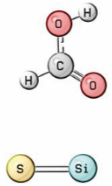

H	1.04080900	-1.13582000	0.00229000
H	3.44657300	0.32248300	-0.00349600
C	1.65796400	-0.23324100	0.00155900
O	2.95371300	-0.51144100	-0.00288800
O	1.21150300	0.88415300	0.00411200
Si	-1.41784000	0.99929700	-0.00191600
S	-1.74419500	-0.92244200	0.00055500

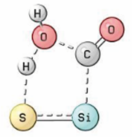

H	0.19741300	1.30617000	0.05678500
H	-1.47552700	1.90599100	-0.31297700
C	-1.22456300	-0.18163900	0.02968800
O	-0.93603800	1.35190600	0.26811300
O	-2.33464600	-0.43444800	-0.22276500
Si	0.56785200	-1.22954900	0.12423900
S	1.67756500	0.48448100	-0.12650400

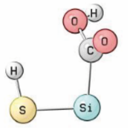

H	0.91648500	1.42314900	-0.62156700
H	-2.08691600	0.98923000	1.31362400
C	-1.16859800	-0.07312300	-0.04529100
O	-1.53799300	1.12948500	0.53457900
O	-1.77448200	-0.52027800	-0.97278200
Si	0.44416500	-1.01352000	0.52052500
S	1.77897000	0.45887400	-0.26262800

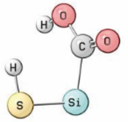

H	0.96100800	1.41411500	-0.69936600
H	-1.19665300	1.43407500	1.20545500
C	-1.17811300	-0.03734800	-0.01009700
O	-1.71719300	1.10304700	0.46583100
O	-1.68230500	-0.60016600	-0.94641400
Si	0.41816300	-0.99265500	0.54033800
S	1.79037700	0.45312600	-0.26034900

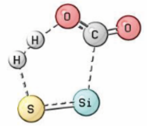

H	0.95658900	1.82002300	0.00042000
H	0.06526100	1.76090500	-0.00018200
C	-1.41574500	0.17405800	0.00001000
O	-1.23207400	1.38108200	-0.00038000
O	-2.31350700	-0.61413900	0.00045800
Si	0.45447700	-1.11655600	-0.00031600
S	1.84216200	0.30443600	0.00021800

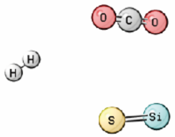

H	-1.67550100	3.98755500	0.54903900
H	-1.97233300	3.38036600	0.24963500
C	-1.82661400	-0.56918500	-0.01700000
O	-2.36026700	0.44869600	-0.10337200
O	-1.30255200	-1.59858700	0.06951100
Si	1.80719000	-0.79593400	0.05474400
S	1.16308800	1.02433700	-0.07451200

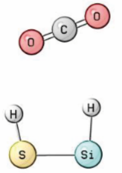

H	-0.63688000	-1.34047300	-0.00041400
H	0.00886700	1.10441400	0.00445100
C	2.35004500	-0.17454500	0.00023000
O	1.75225800	-1.16528800	0.00401700
O	2.95807400	0.80585800	-0.00359400
Si	-1.50281000	1.25948200	0.00163100
S	-1.88222400	-0.84212400	-0.00197700

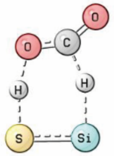

H	0.18622500	-1.14185300	0.00004900
H	-0.57613200	0.78365900	0.00010900
C	-1.64019900	-0.08177500	0.00001100
O	-1.18419500	-1.21564100	-0.00003800
O	-2.63633800	0.55208300	0.00001300
Si	1.05515000	1.25375300	-0.00001700
S	1.62645400	-0.71220200	0.00001300

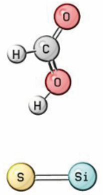

H	0.59434600	-0.59453200	0.71057500
H	1.93359700	-1.13257100	-0.84496100
C	2.24874000	-0.30207300	-0.19507700
O	1.36997400	-0.02003500	0.78074100
O	3.26463700	0.29812000	-0.33431600
Si	-1.71908900	1.07806000	-0.07062600
S	-1.81437700	-0.86112400	-0.07986200

Figure S10. Cartesian coordinates of stationary points and transition states in the reactions of HSiSH with CO_2.

Reactions of HSiSH with CO_2: Part II Intrinsic reaction coordinate calculation (IRC)

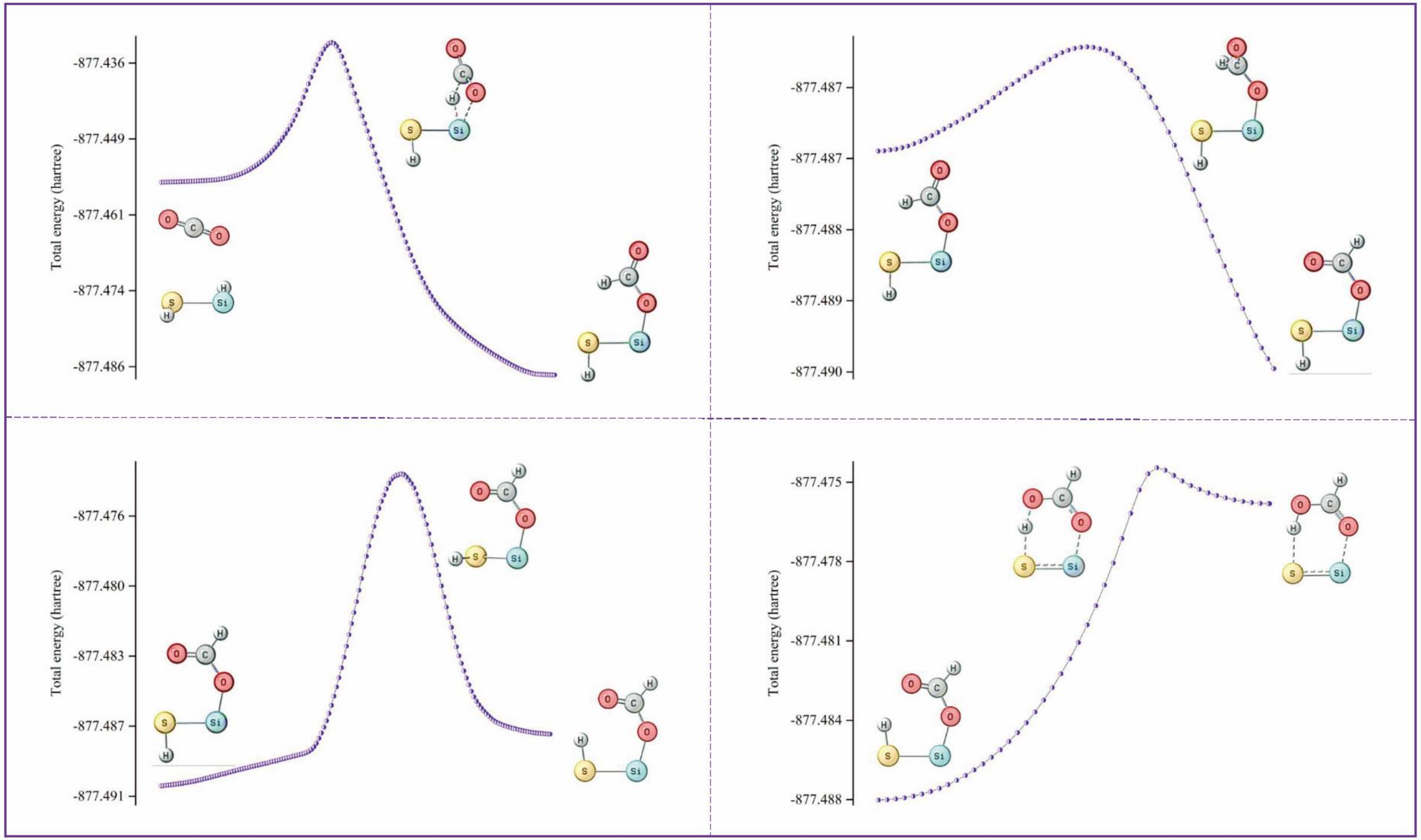

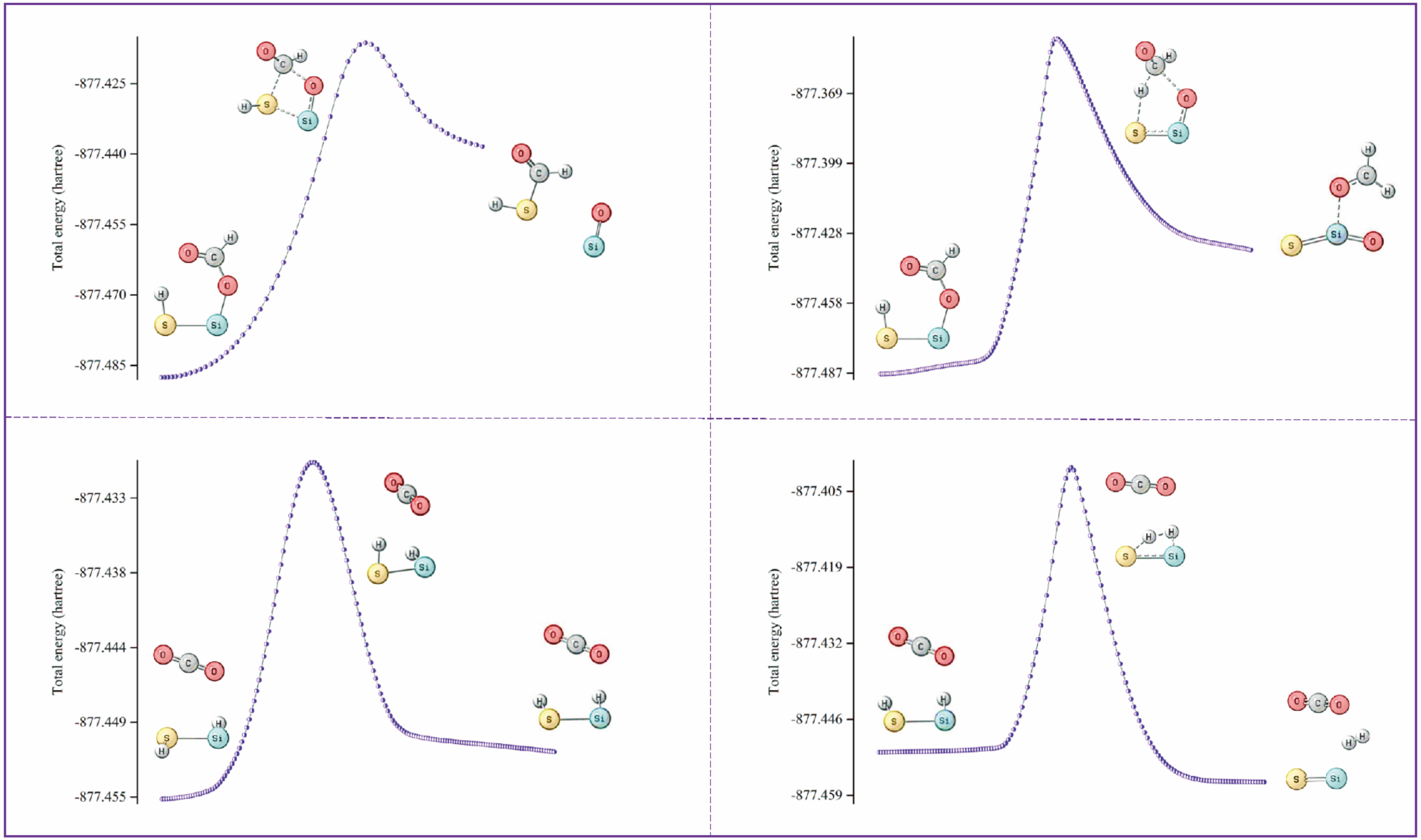
-877.425
-877.440
-877.455
-877.470
-877.485
Total energy (hartree)
-877.369
-877.399
-877.428
-877.458
-877.487
Total energy (hartree)
-877.433
-877.438
-877.444
-877.449
-877.455
Total energy (hartree)
-877.405
-877.419
-877.432
-877.446
-877.459
Total energy (hartree)

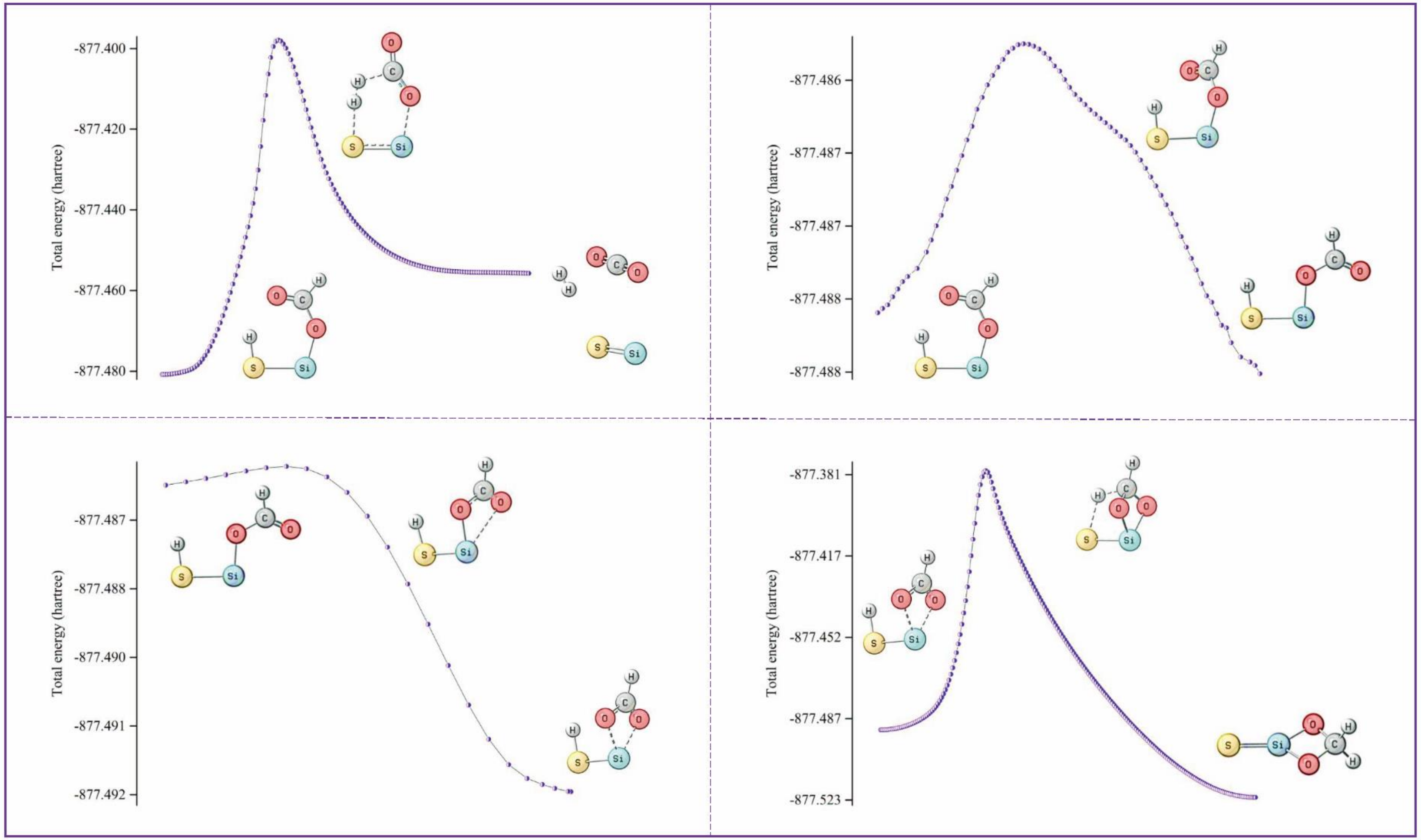

Total energy (hartree)
-877.400
-877.420
-877.440
-877.460
-877.480
Total energy (hartree)
-877.486
-877.487
-877.487
-877.488
-877.488
Total energy (hartree)
-877.487
-877.488
-877.490
-877.491
-877.492
Total energy (hartree)
-877.381
-877.417
-877.452
-877.487
-877.523

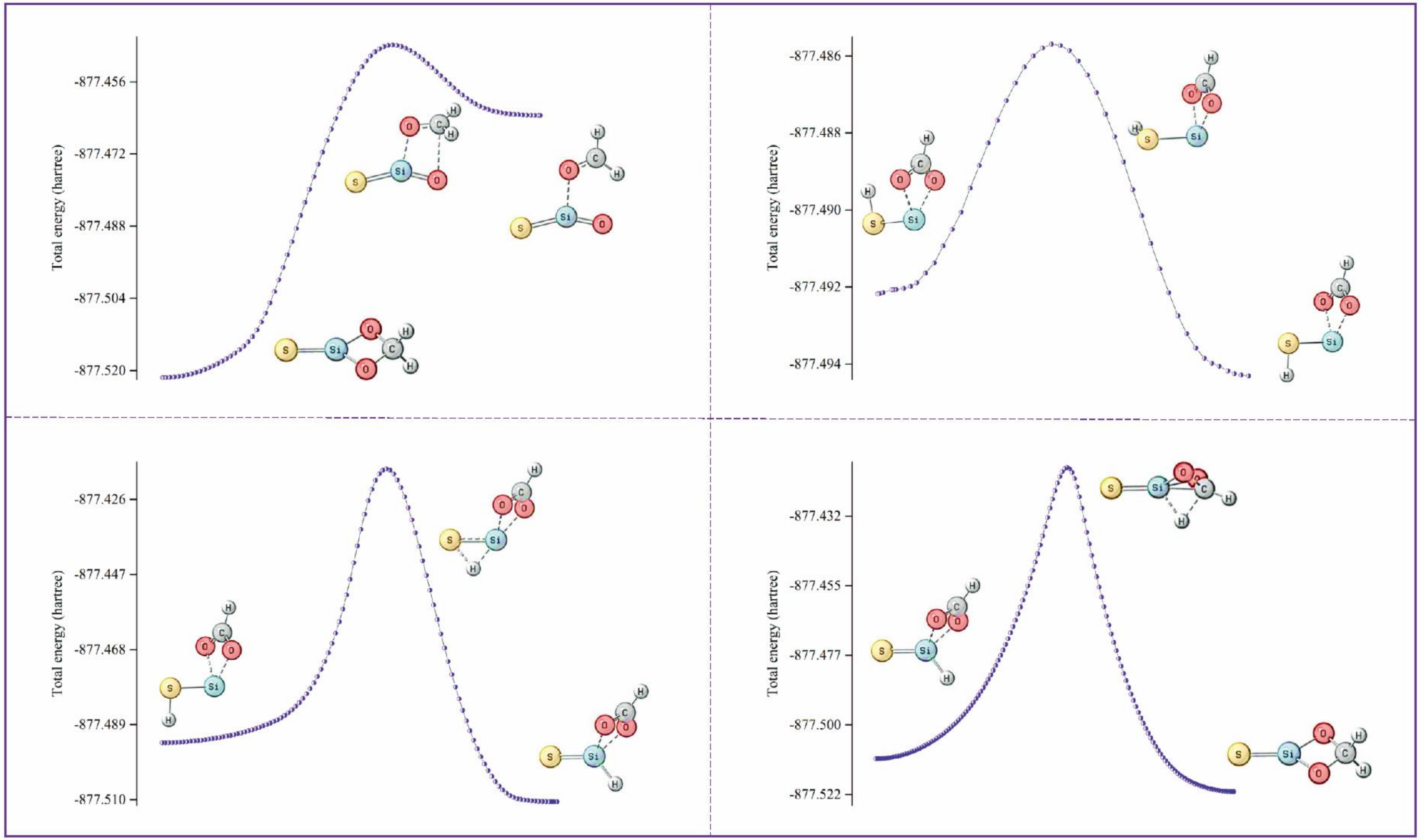
Total energy (hartree)
-877.456
-877.472
-877.488
-877.504
-877.520
Total energy (hartree)
-877.486
-877.488
-877.490
-877.492
-877.494
Total energy (hartree)
-877.426
-877.447
-877.468
-877.489
-877.510
Total energy (hartree)
-877.432
-877.455
-877.477
-877.500
-877.522

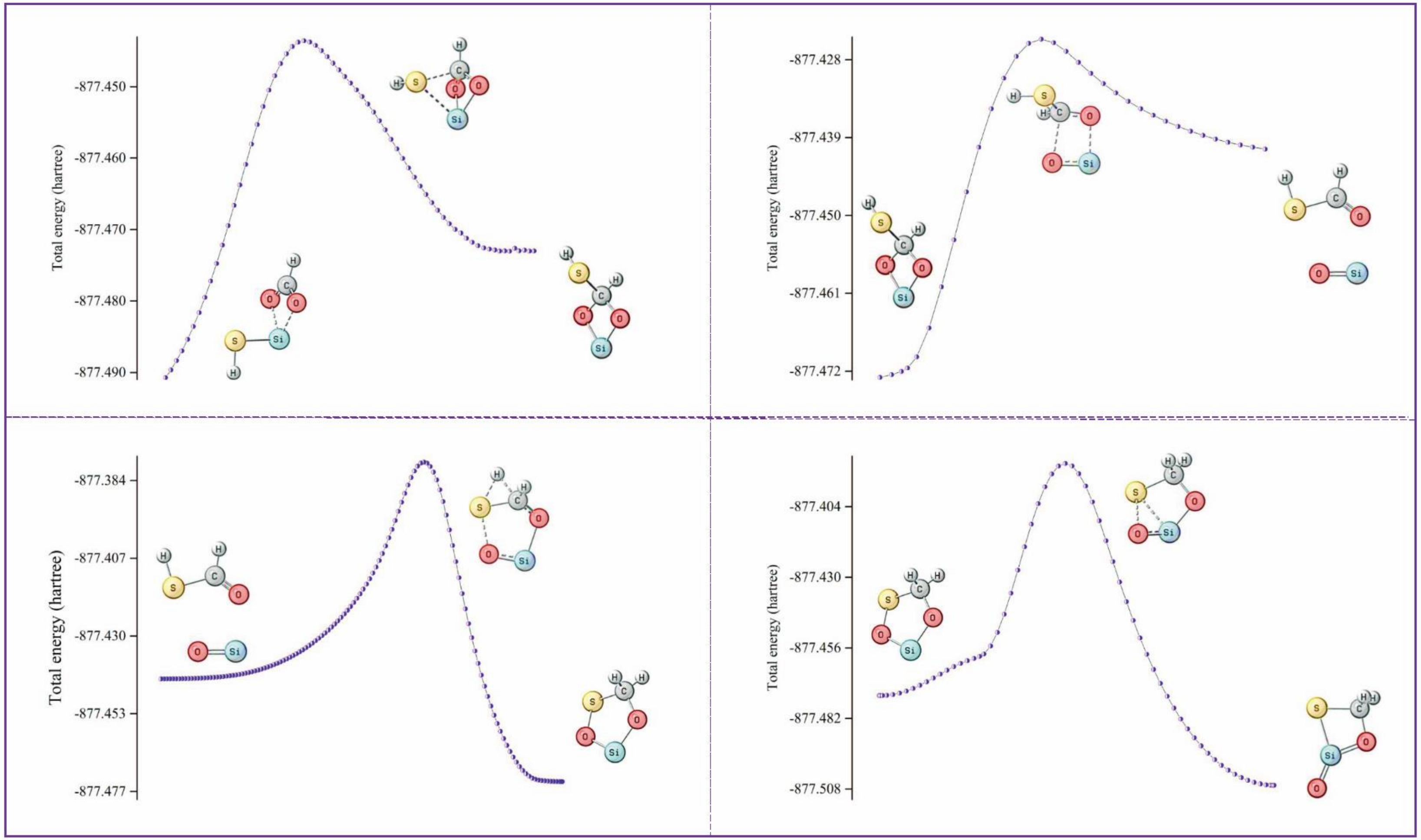

Total energy (hartree)
-877.450
-877.460
-877.470
-877.480
-877.490
Total energy (hartree)
-877.428
-877.439
-877.450
-877.461
-877.472
Total energy (hartree)
-877.384
-877.407
-877.430
-877.453
-877.477
Total energy (hartree)
-877.404
-877.430
-877.456
-877.482
-877.508

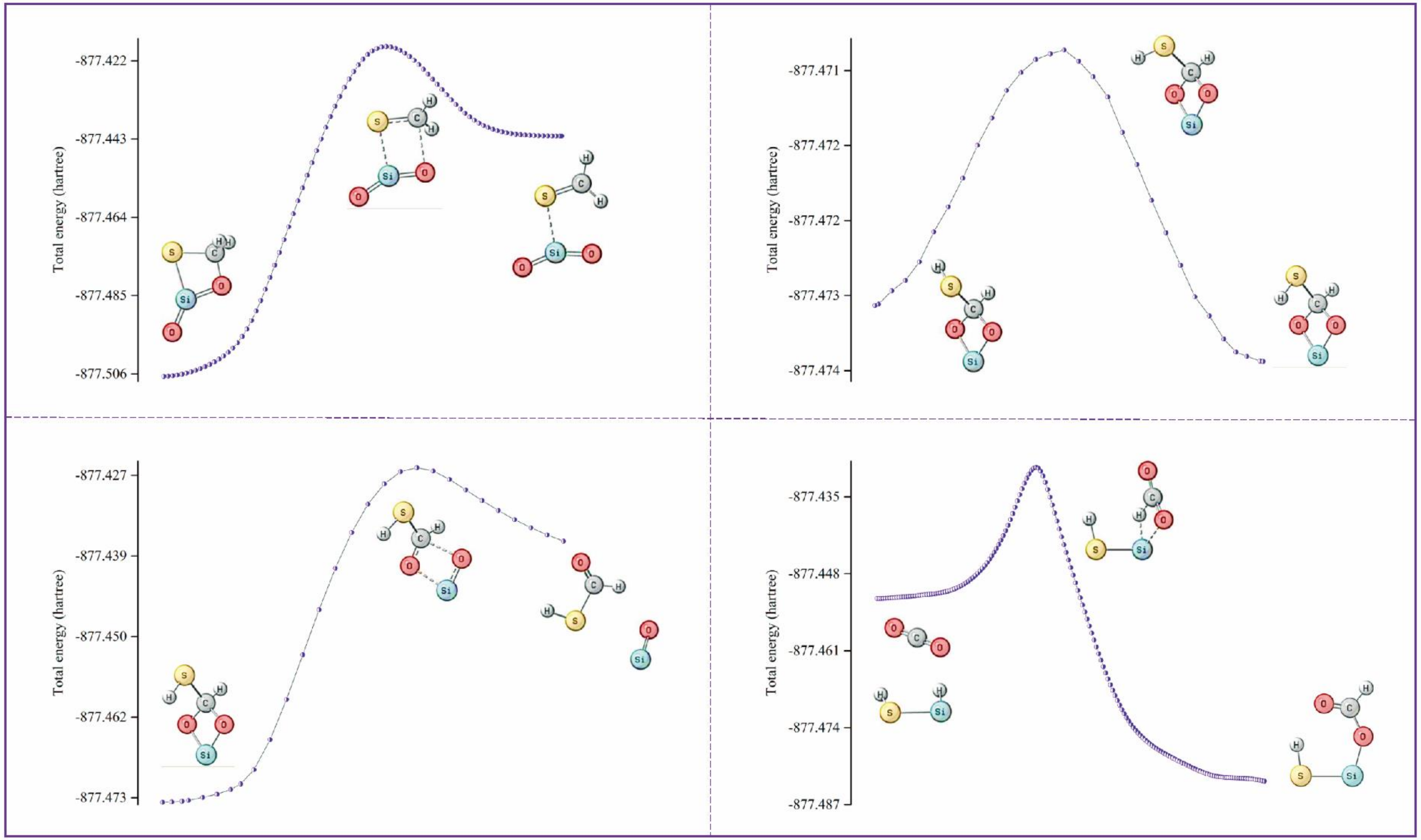
Total energy (hartree)
-877.422
-877.443
-877.464
-877.485
-877.506
-877.471
-877.472
-877.472
-877.473
-877.474
-877.427
-877.439
-877.450
-877.462
-877.473
-877.435
-877.448
-877.461
-877.474
-877.487

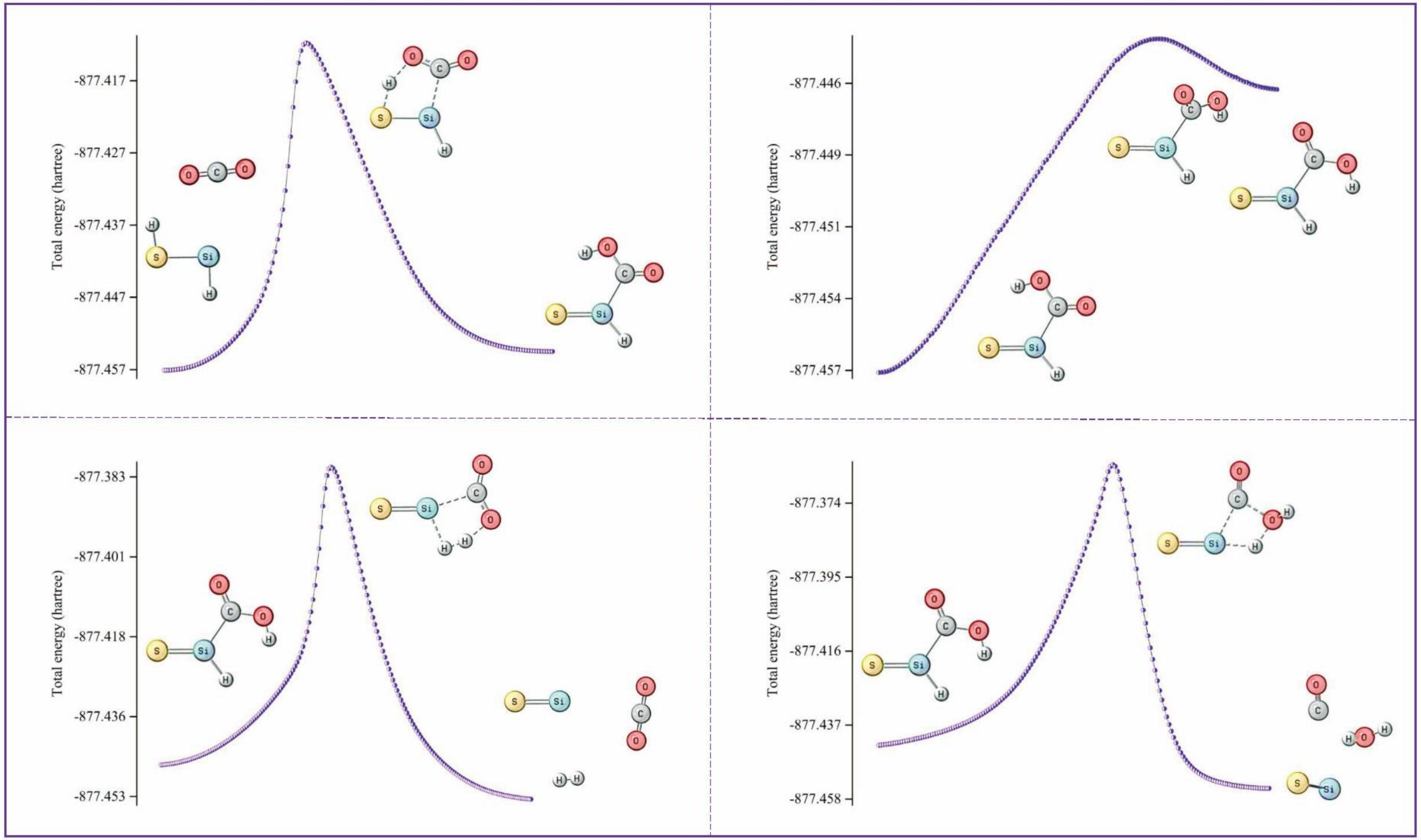
-877.417
-877.427
-877.437
-877.447
-877.457
Total energy (hartree)
-877.446
-877.449
-877.451
-877.454
-877.457
Total energy (hartree)
-877.383
-877.401
-877.418
-877.436
-877.453
Total energy (hartree)
-877.374
-877.395
-877.416
-877.437
-877.458
Total energy (hartree)

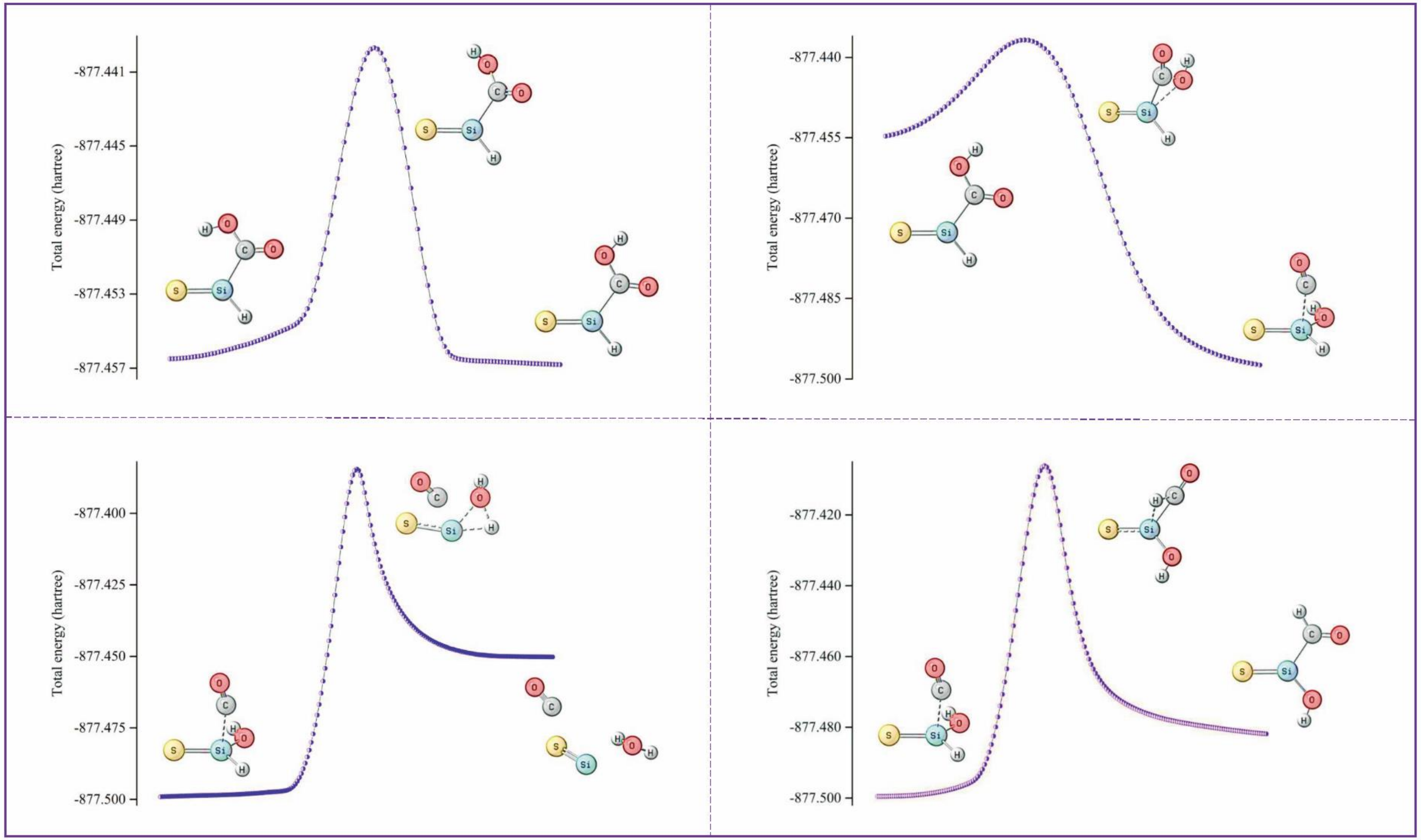

Total energy (hartree)
-877.441
-877.445
-877.449
-877.453
-877.457
-877.440
-877.455
-877.470
-877.485
-877.500
-877.400
-877.425
-877.450
-877.475
-877.500
-877.420
-877.440
-877.460
-877.480
-877.500

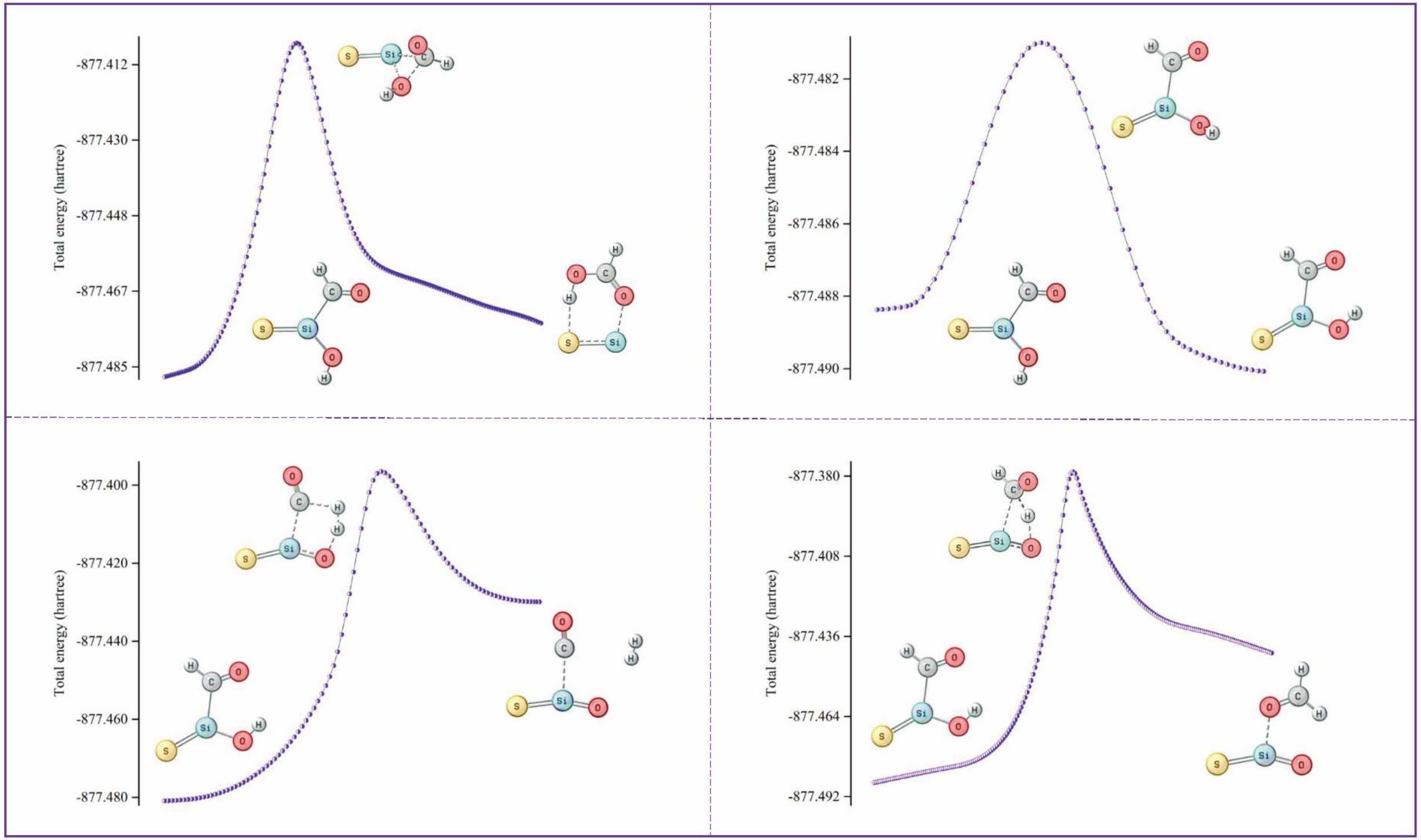

Total energy (hartree)
-877.412
-877.430
-877.448
-877.467
-877.485
-877.482
-877.484
-877.486
-877.488
-877.490
-877.400
-877.420
-877.440
-877.460
-877.480
-877.380
-877.408
-877.436
-877.464
-877.492

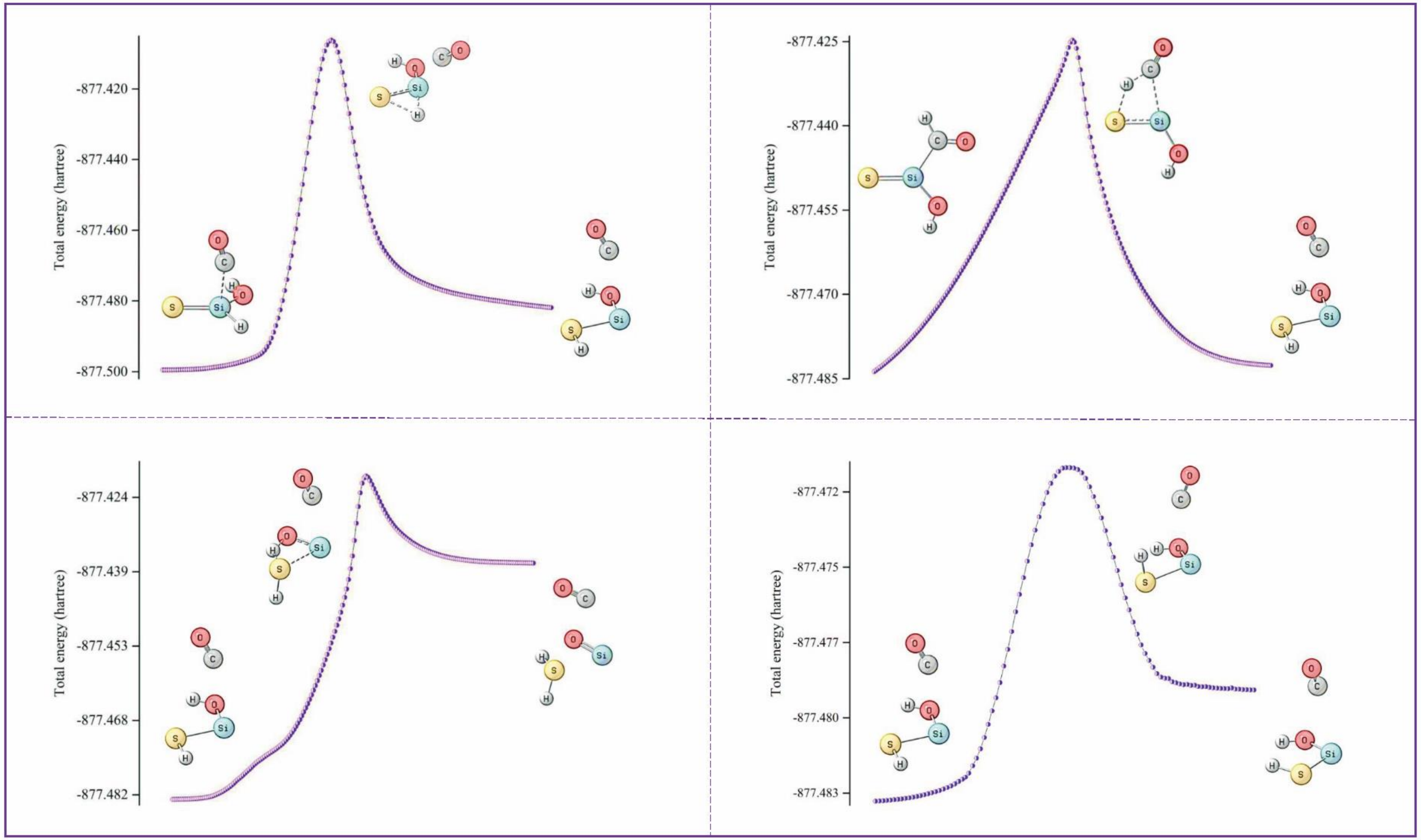

Total energy (hartree)
-877.420
-877.440
-877.460
-877.480
-877.500
-877.425
-877.440
-877.455
-877.470
-877.485
-877.424
-877.439
-877.453
-877.468
-877.482
-877.472
-877.475
-877.477
-877.480
-877.483

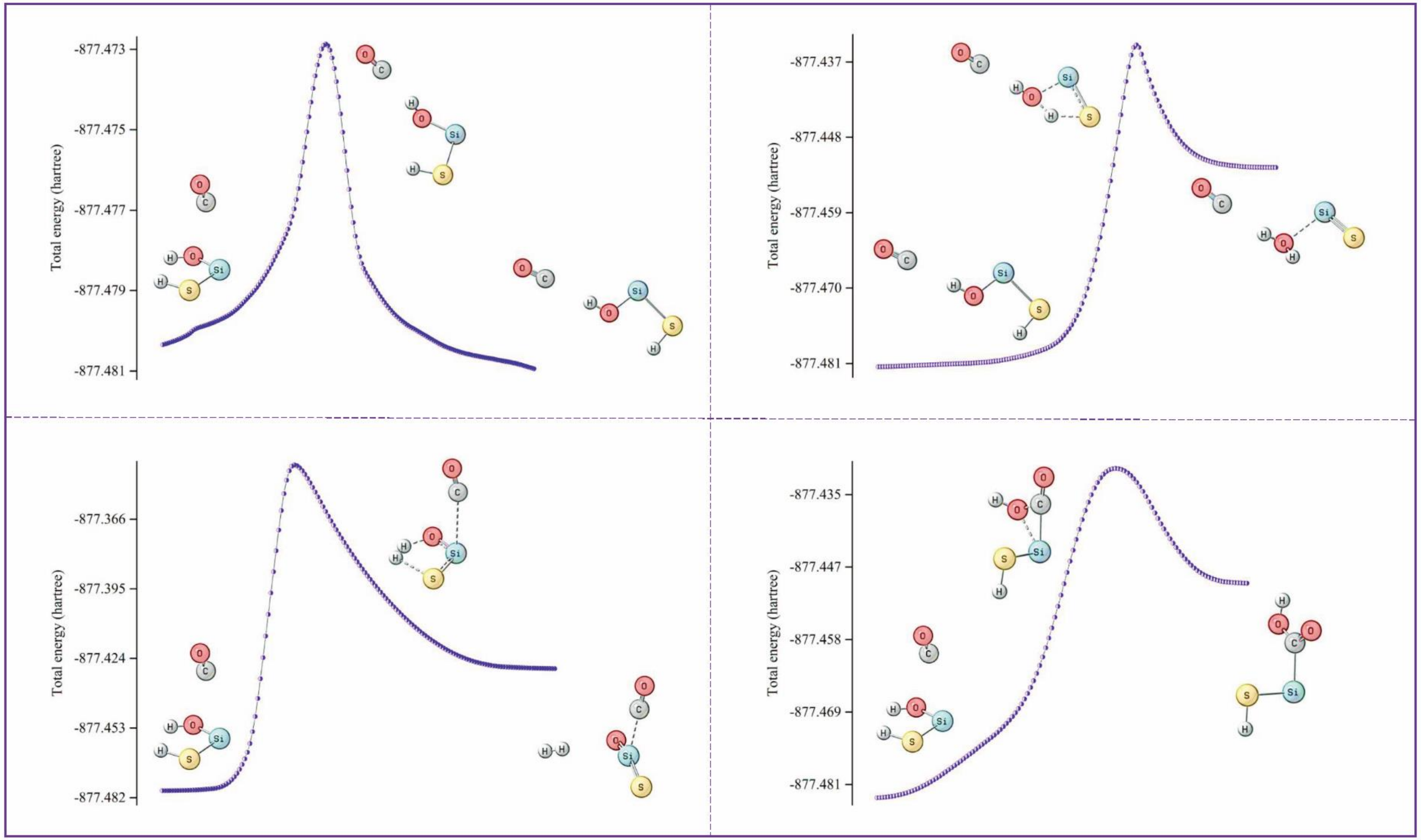
Total energy (hartree)
-877.473
-877.475
-877.477
-877.479
-877.481
-877.437
-877.448
-877.459
-877.470
-877.481
-877.366
-877.395
-877.424
-877.453
-877.482
-877.435
-877.447
-877.458
-877.469
-877.481

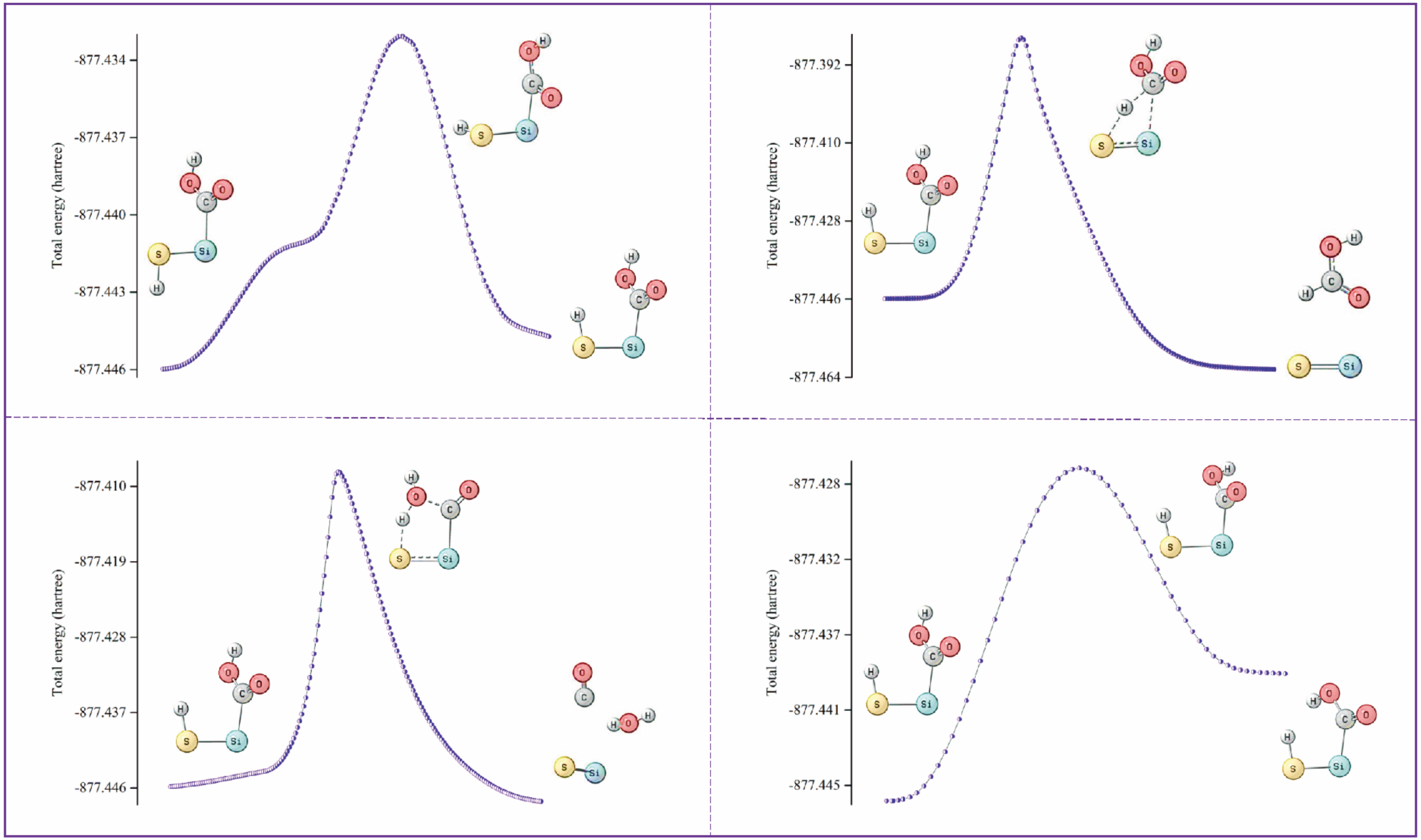
Total energy (hartree)
-877.434
-877.437
-877.440
-877.443
-877.446
Total energy (hartree)
-877.392
-877.410
-877.428
-877.446
-877.464
Total energy (hartree)
-877.410
-877.419
-877.428
-877.437
-877.446
Total energy (hartree)
-877.428
-877.432
-877.437
-877.441
-877.445

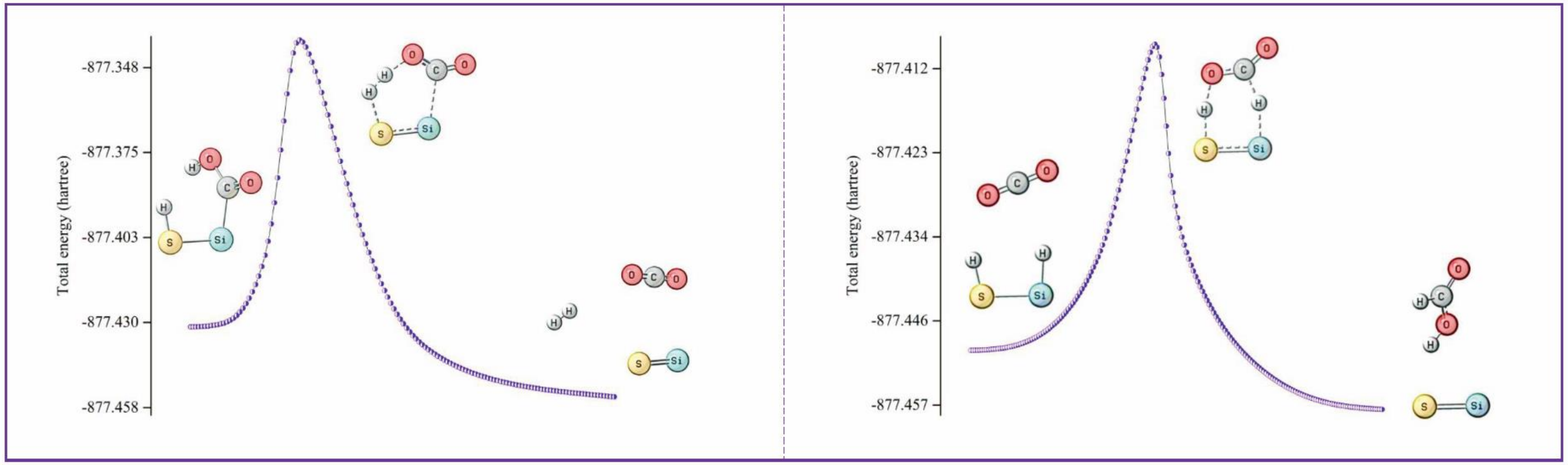

Figure S11. Intrinsic reaction coordinate calculation for the reactions of HSiSH with CO_2

Reactions of HSiSH with CO_2:   Part III Reaction pathways of HSiSH + CO_2

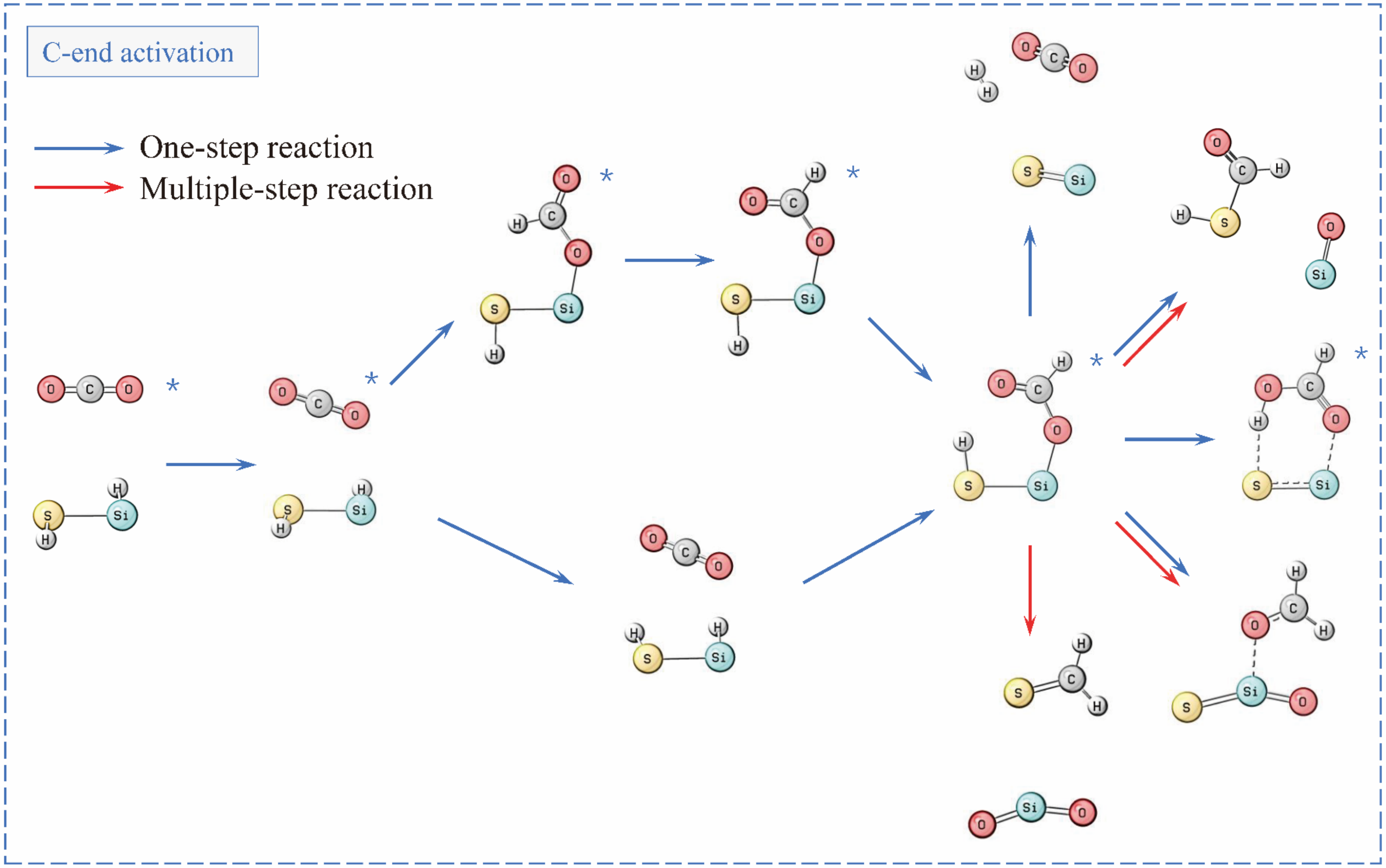

Figure S12. A brief summary (TS omitted) of C-end activation pathways for the reaction of HSiSH + CO_2 (the optimal path is marked by asterisk).

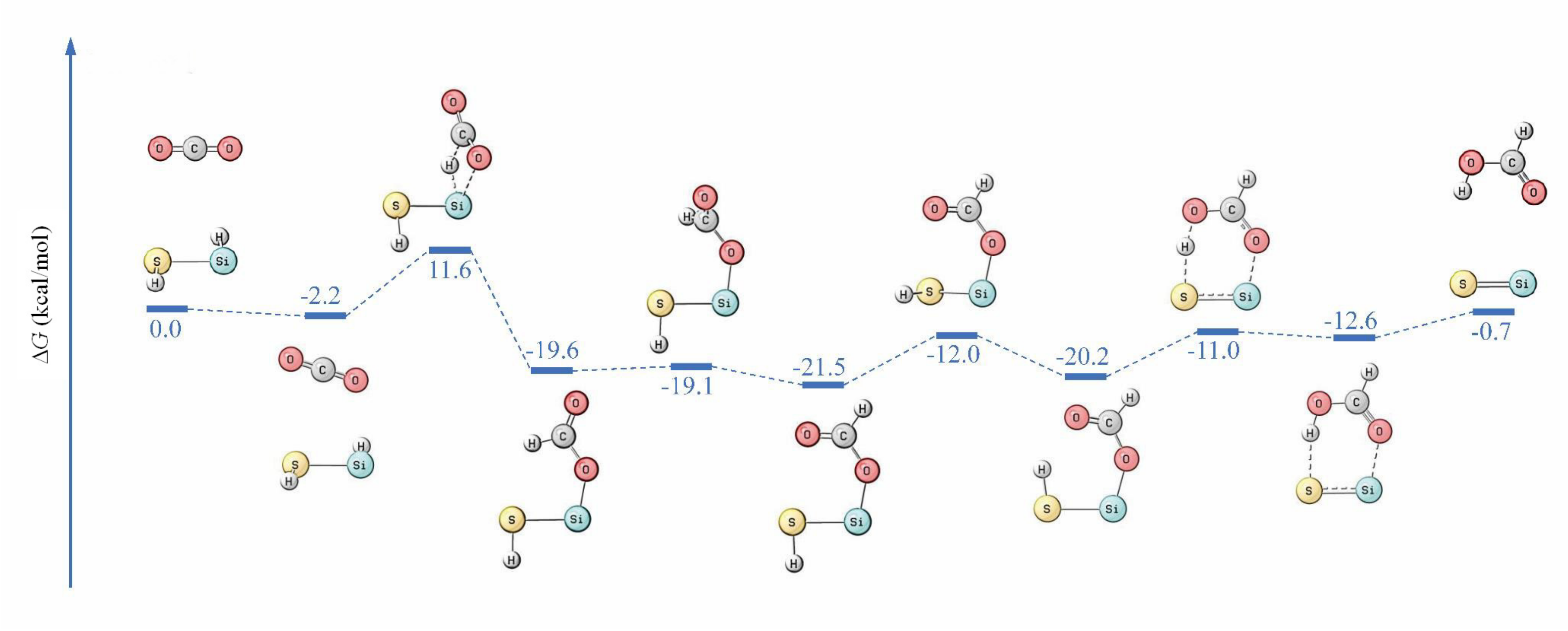

ΔG (kcal/mol)
0.0
-2.2
11.6
-19.6
-19.1
-21.5
-12.0
-20.2
-11.0
-12.6
-0.7

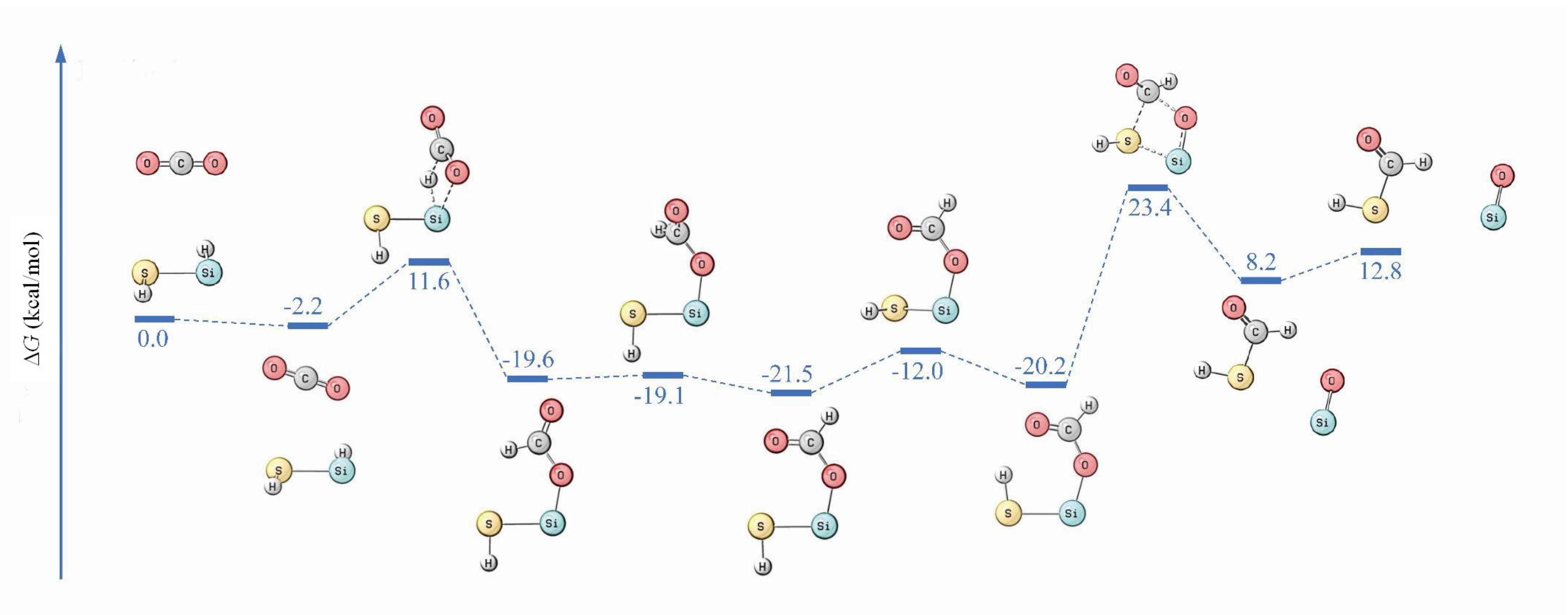

ΔG (kcal/mol)
0.0
-2.2
11.6
-19.6
-19.1
-21.5
-12.0
-20.2
23.4
8.2
12.8

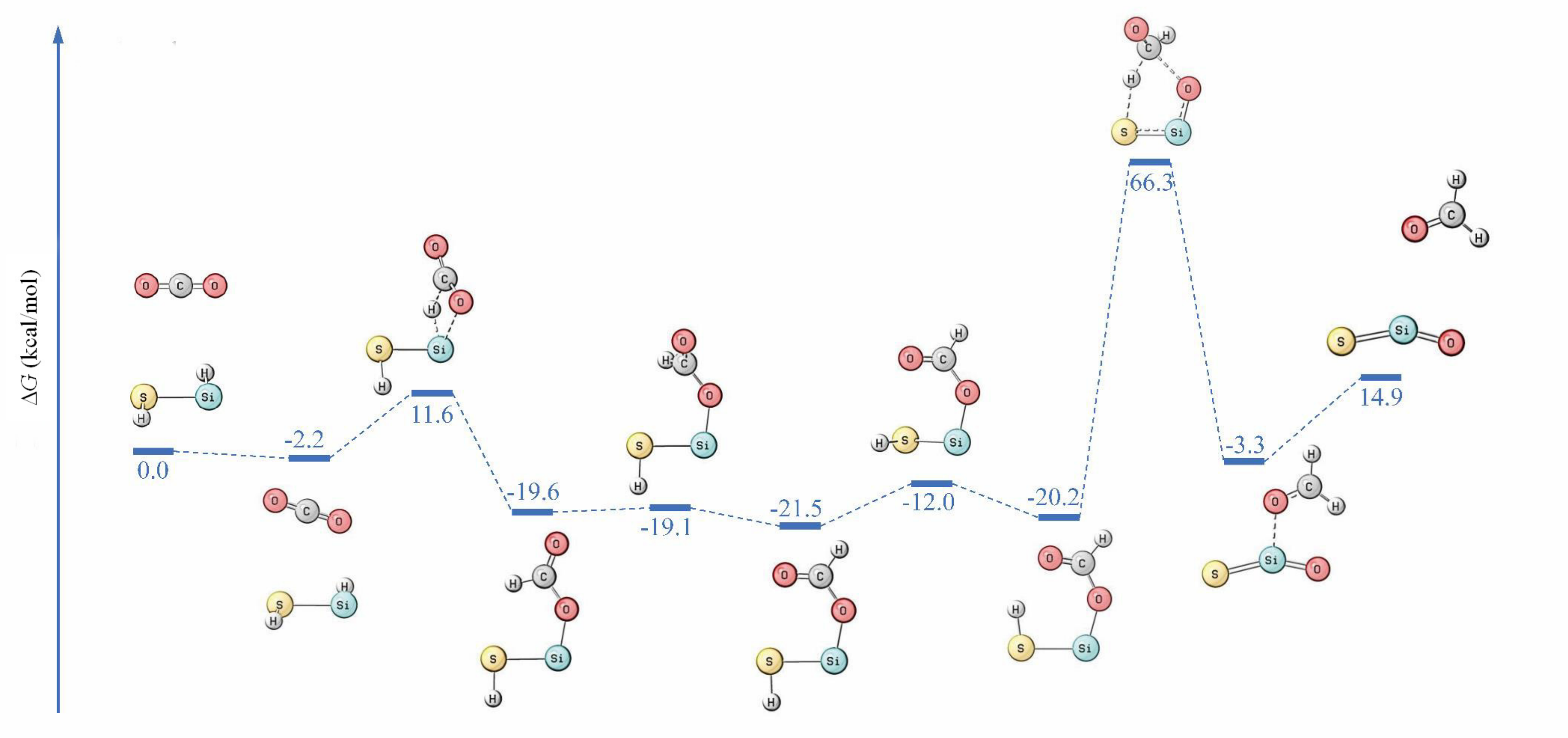
ΔG (kcal/mol)
0.0
-2.2
11.6
-19.6
-19.1
-21.5
-12.0
-20.2
66.3
-3.3
14.9
O
C
S
Si
H

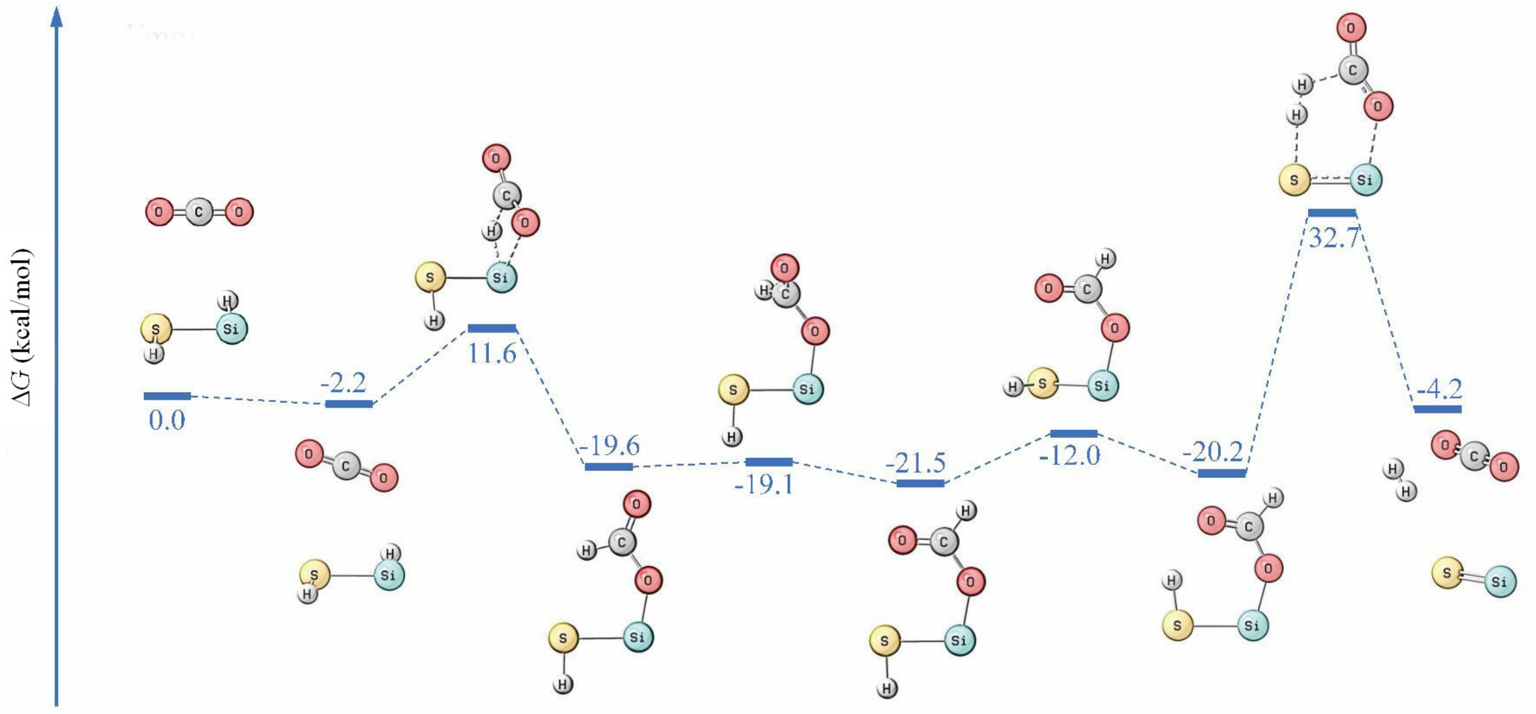

ΔG (kcal/mol)
0.0
-2.2
11.6
-19.6
-19.1
-21.5
-12.0
-20.2
32.7
-4.2

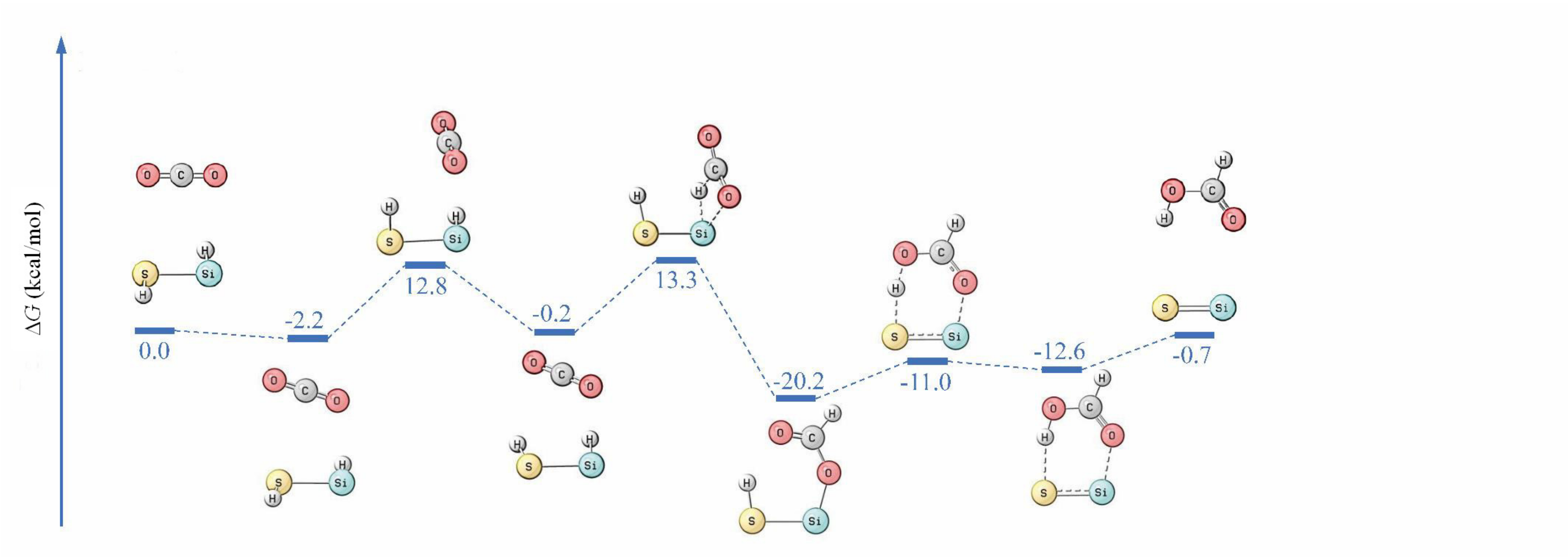

ΔG (kcal/mol)
0.0
-2.2
12.8
-0.2
13.3
-20.2
-11.0
-12.6
-0.7

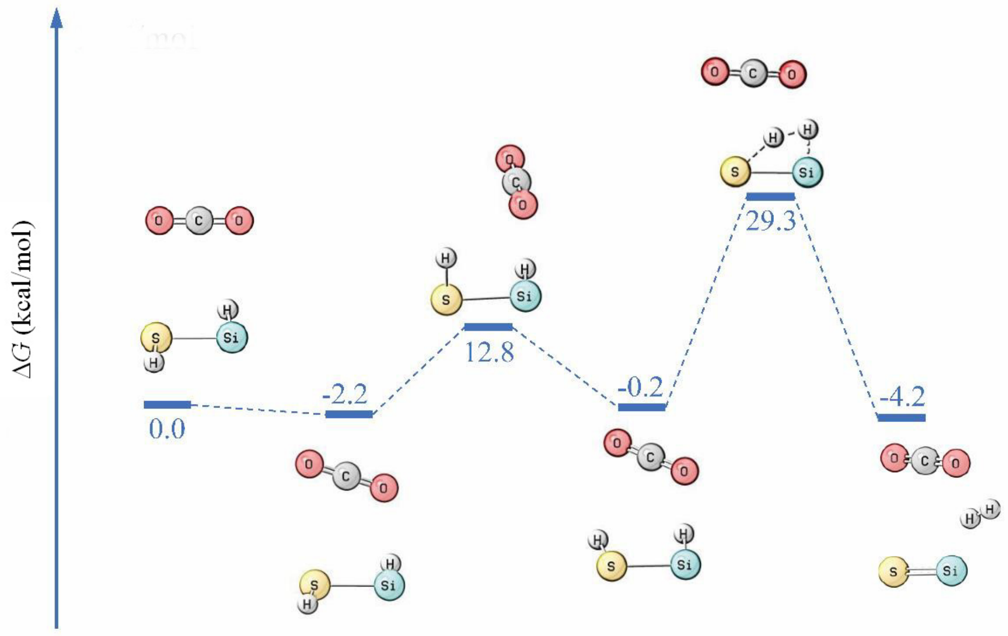
ΔG (kcal/mol)
0.0
-2.2
12.8
-0.2
29.3
-4.2
O
C
S
Si
H

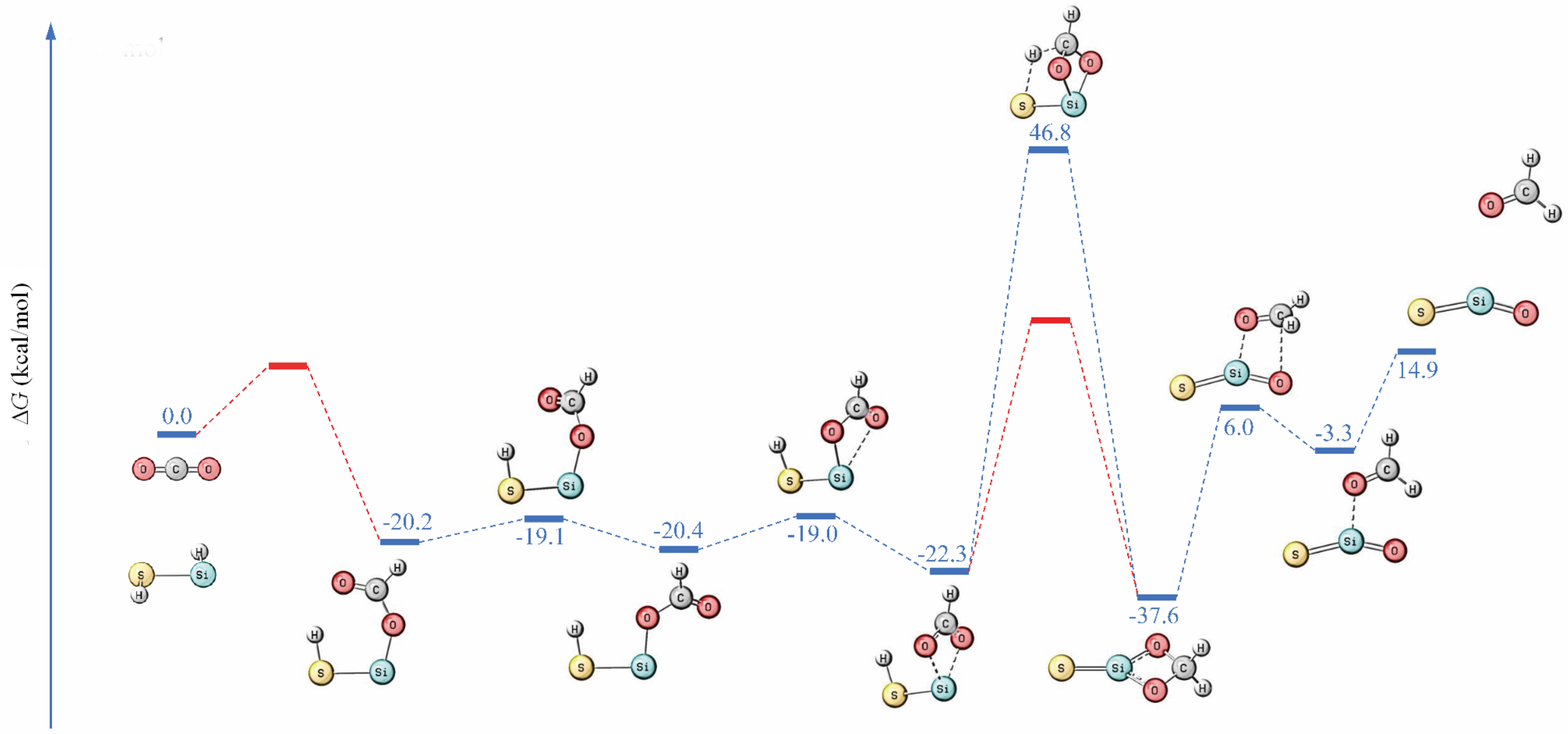
ΔG (kcal/mol)
0.0
-20.2
-19.1
-20.4
-19.0
-22.3
46.8
-37.6
6.0
-3.3
14.9

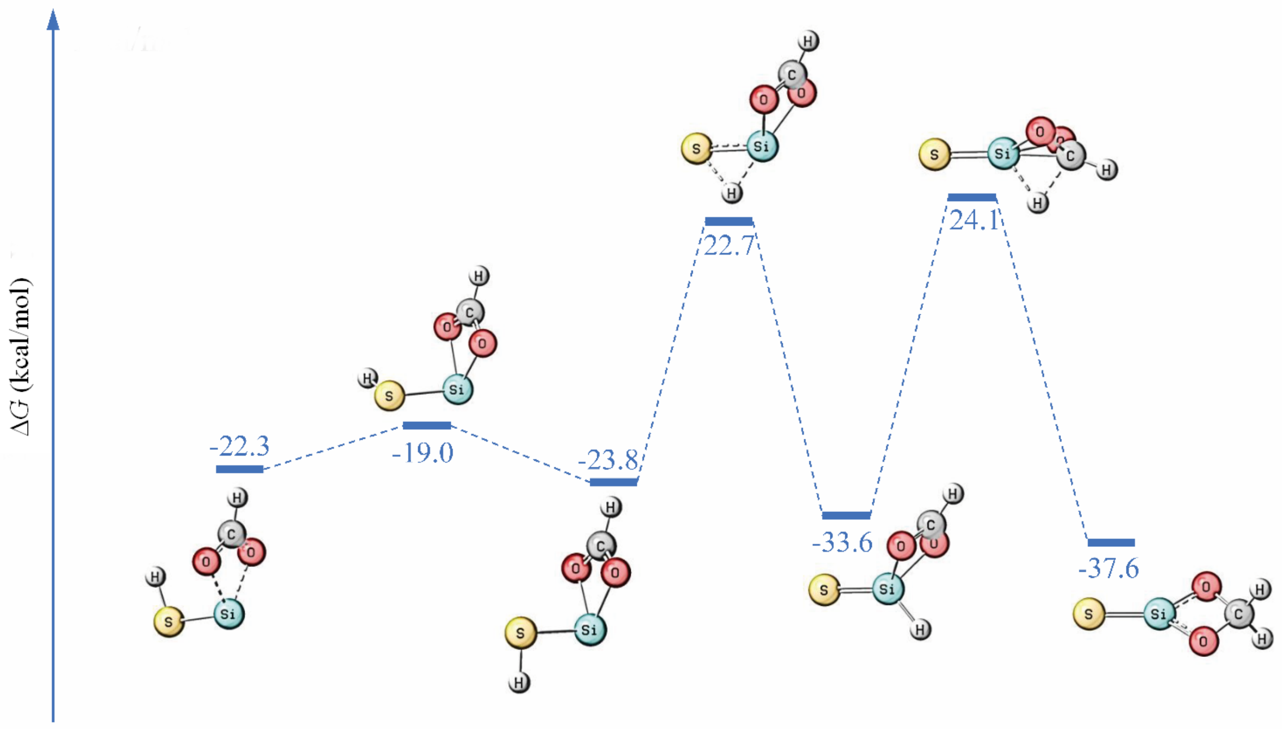
ΔG (kcal/mol)
-22.3
-19.0
-23.8
22.7
-33.6
24.1
-37.6
H
S
Si
O
C

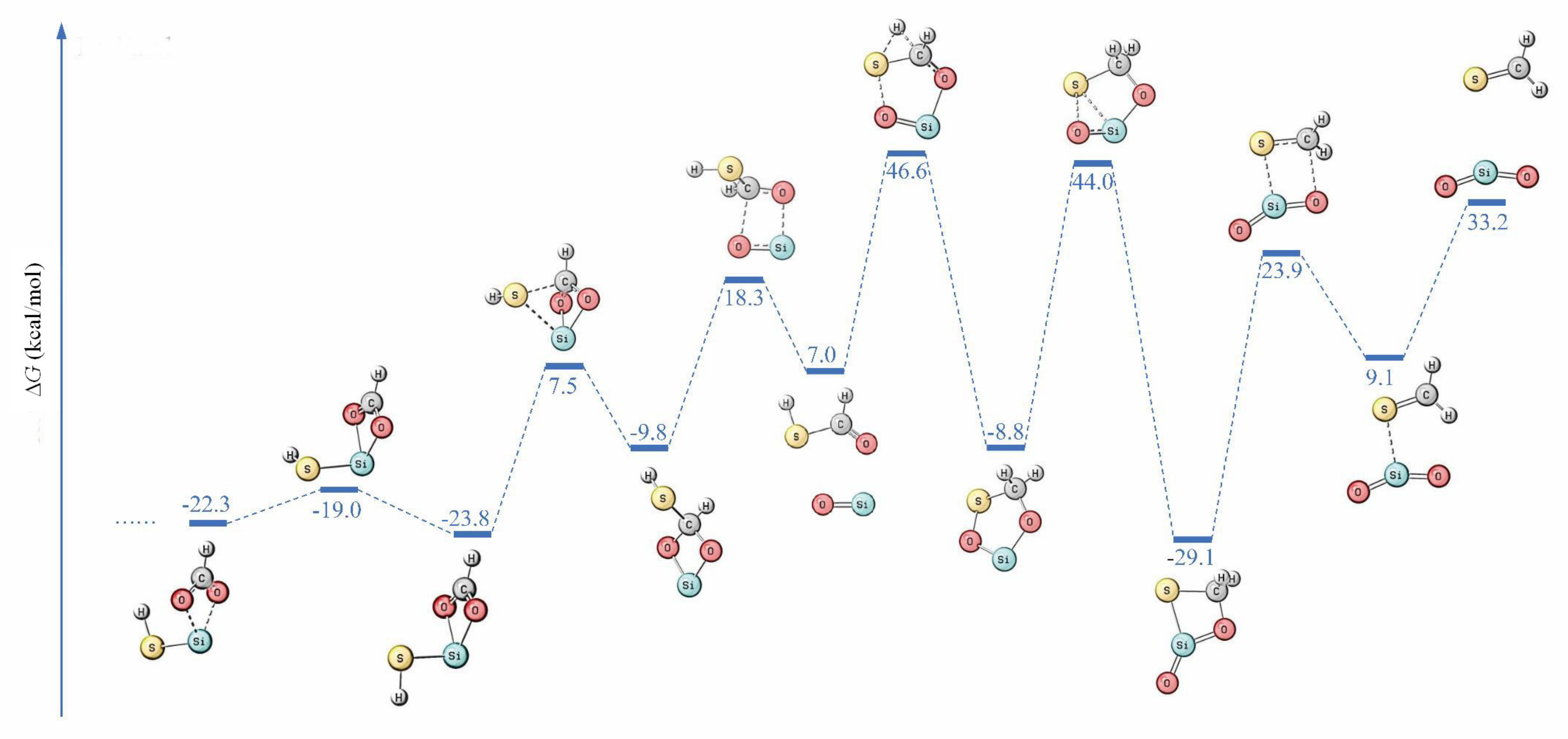
ΔG (kcal/mol)
-22.3
-19.0
-23.8
7.5
-9.8
18.3
7.0
46.6
-8.8
44.0
-29.1
23.9
9.1
33.2

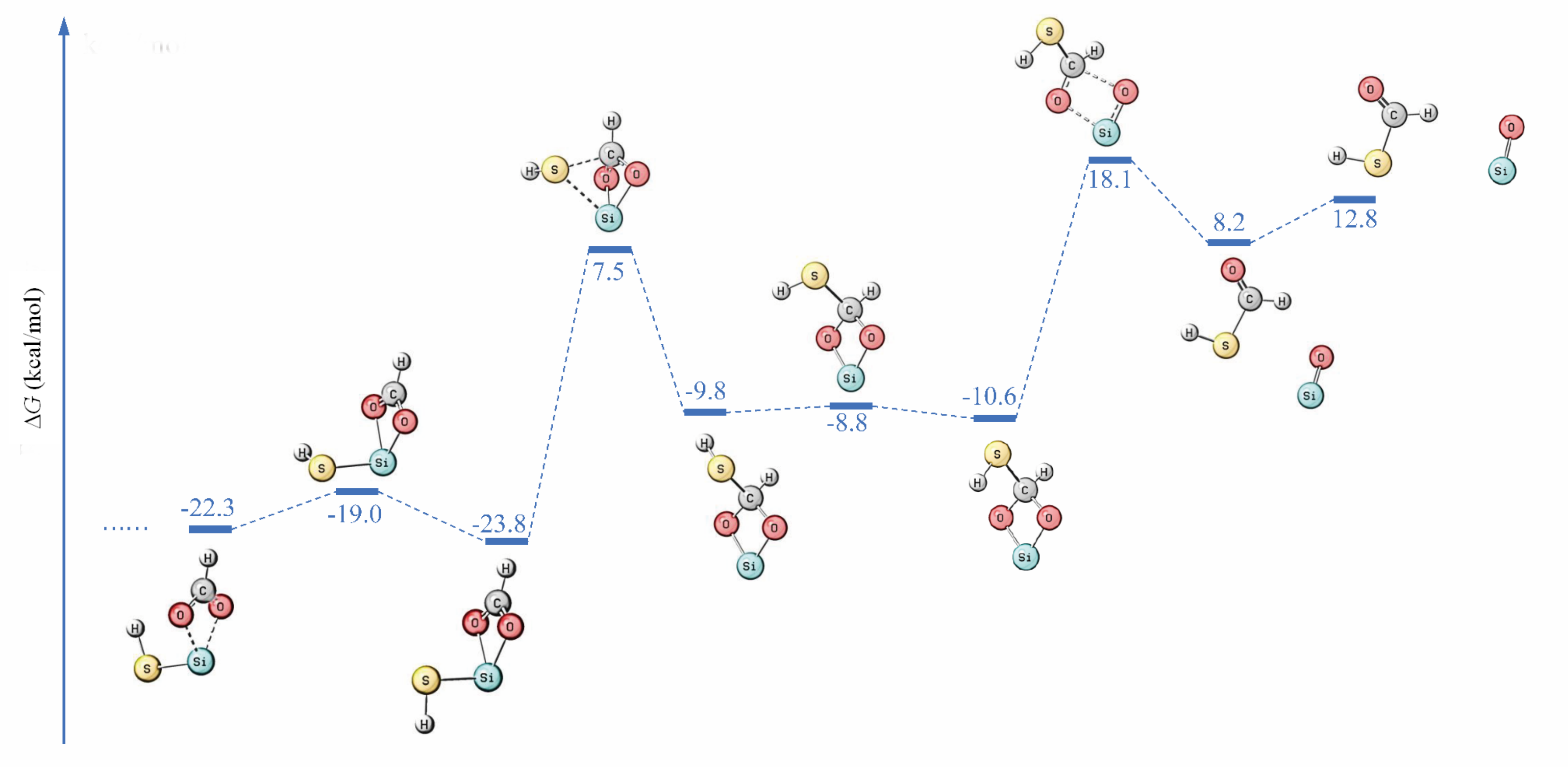

Figure S13. C-end activation pathways for the reaction of HSiSH + CO_2.

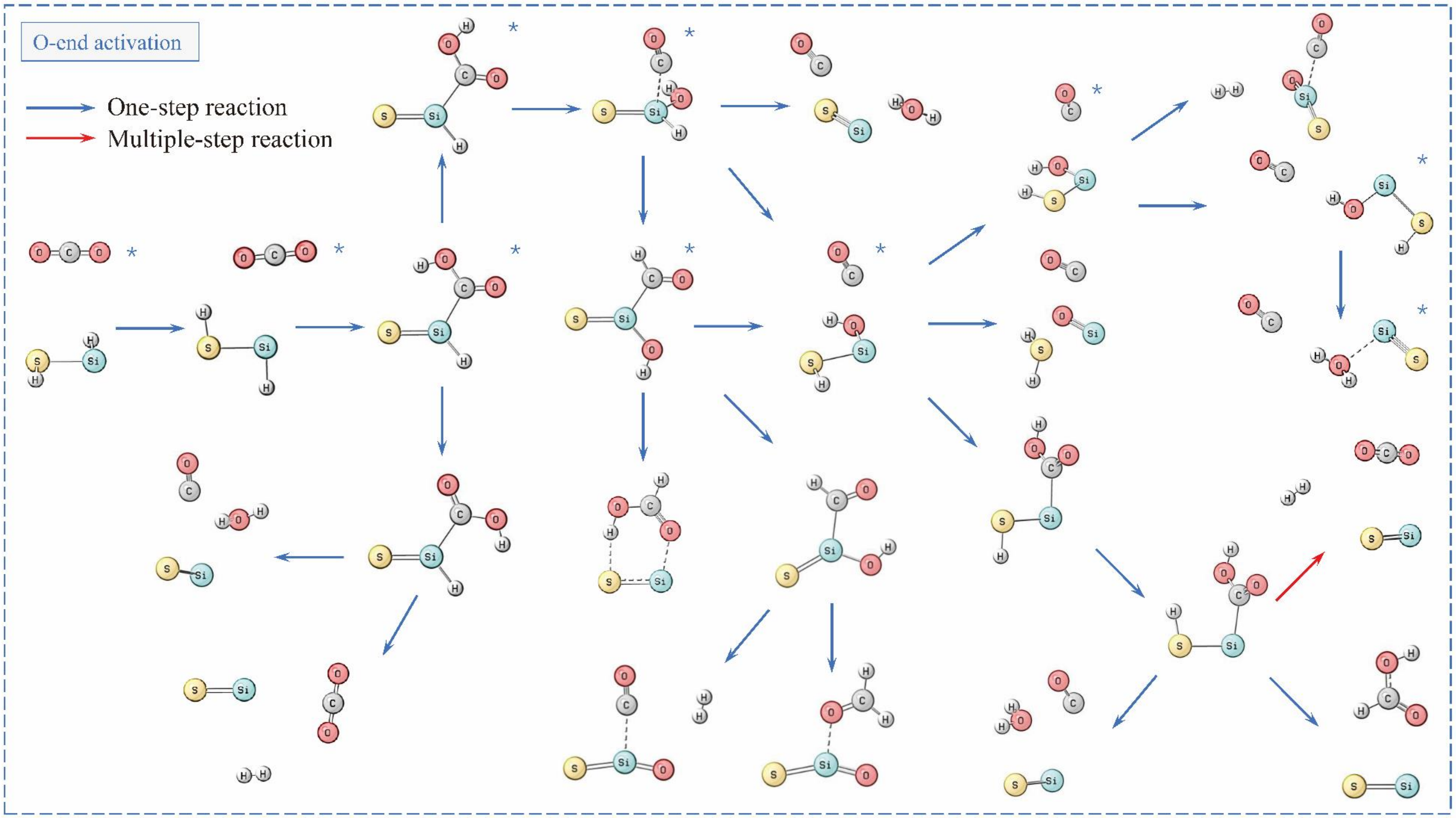

Figure S14. A brief summary (TS omitted) of O-end activation pathways for the reaction of HSiSH + CO_2 (the optimal path is marked by asterisk).

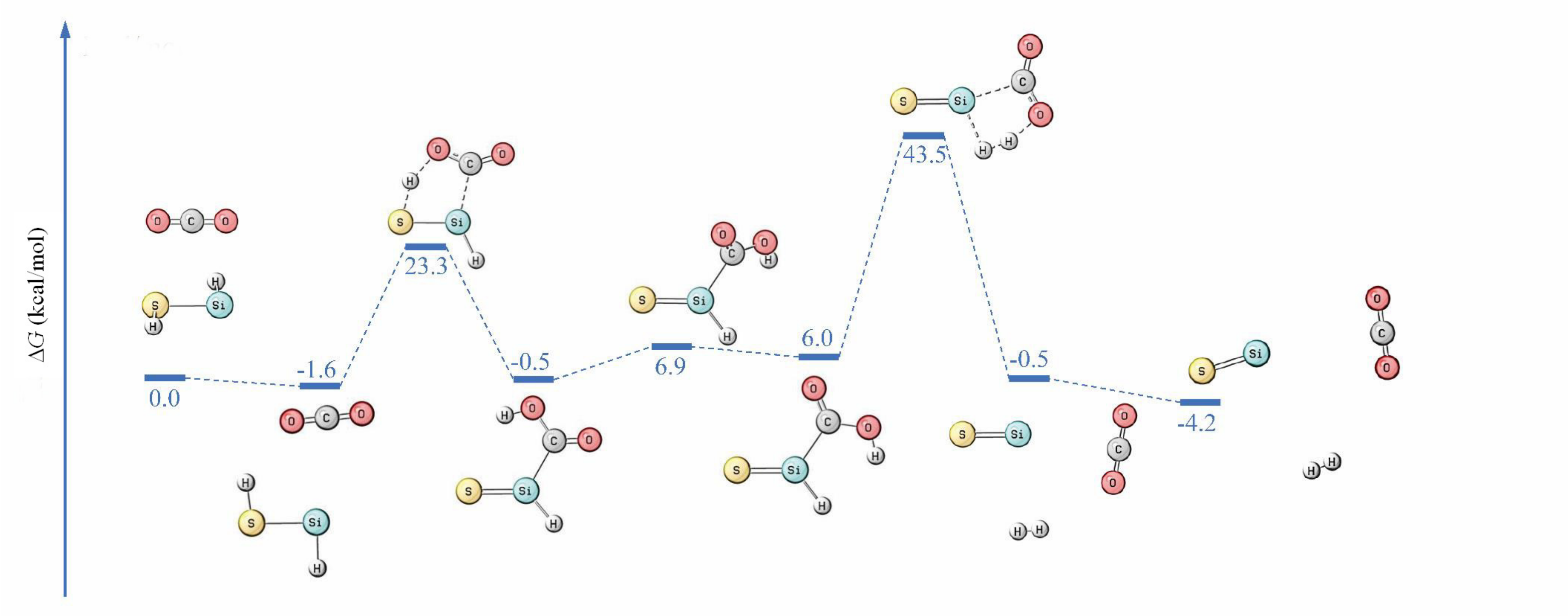
ΔG (kcal/mol)
0.0
-1.6
23.3
-0.5
6.9
6.0
43.5
-0.5
-4.2

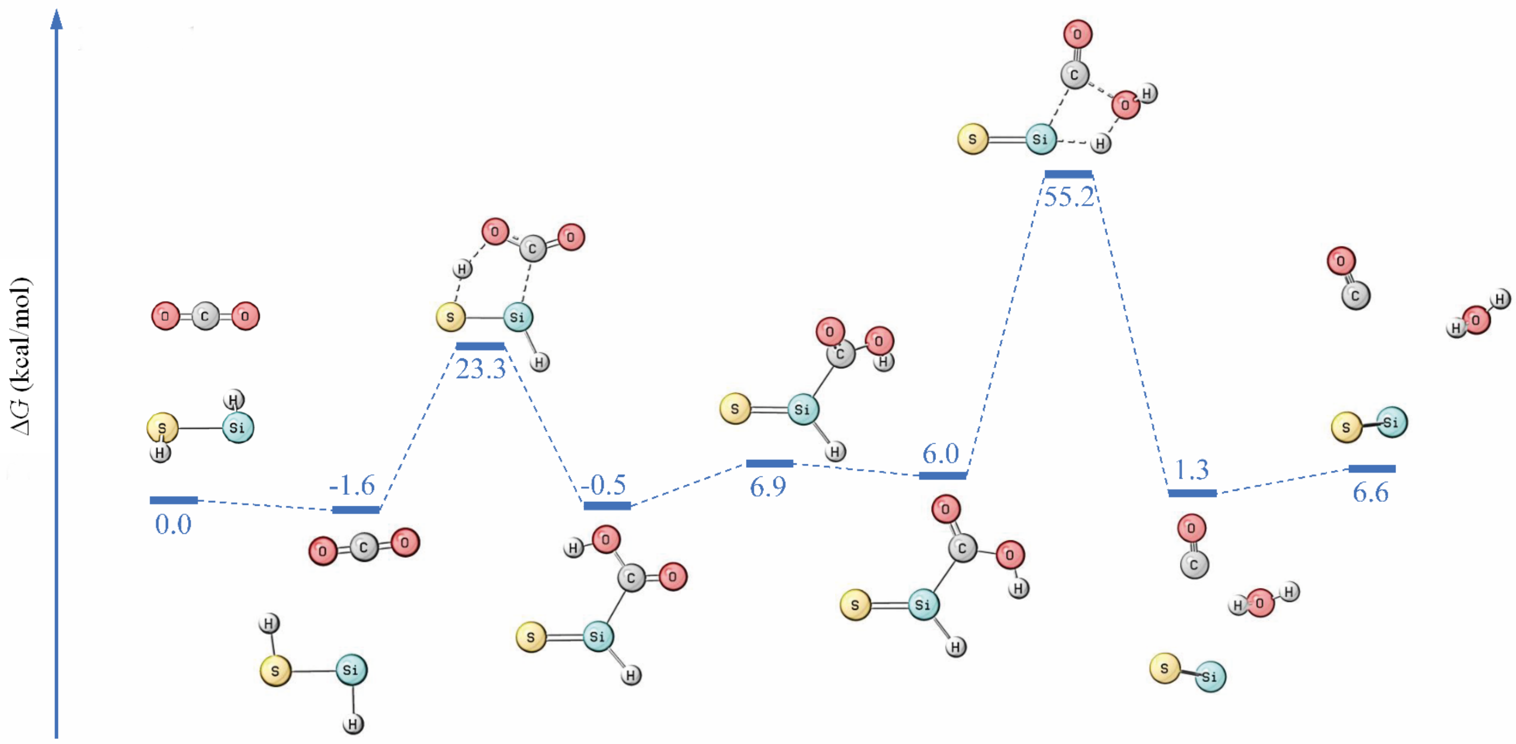

ΔG (kcal/mol)
0.0
-1.6
23.3
-0.5
6.9
6.0
55.2
1.3
6.6

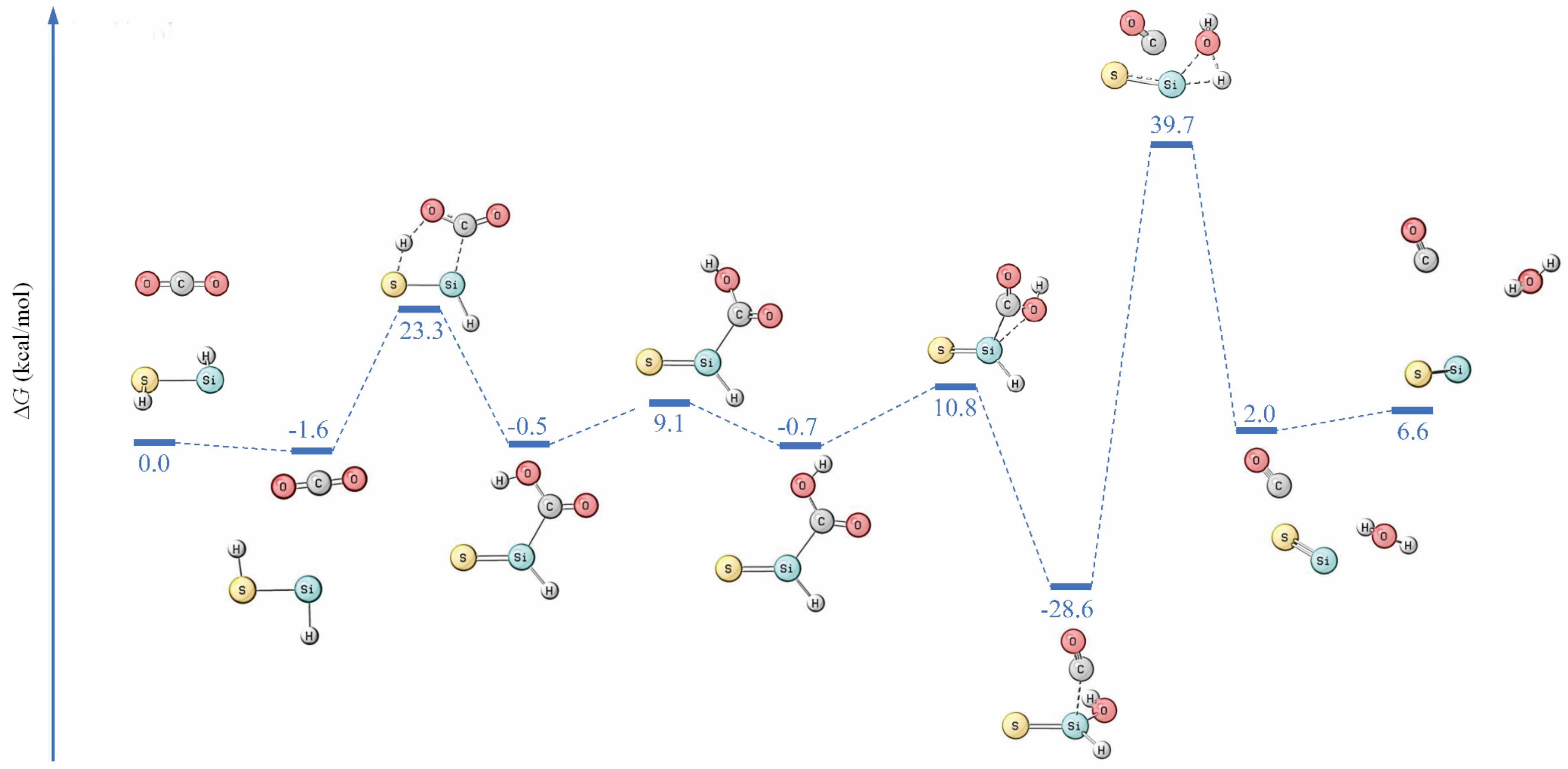

ΔG (kcal/mol)
0.0
-1.6
23.3
-0.5
9.1
-0.7
10.8
-28.6
39.7
2.0
6.6

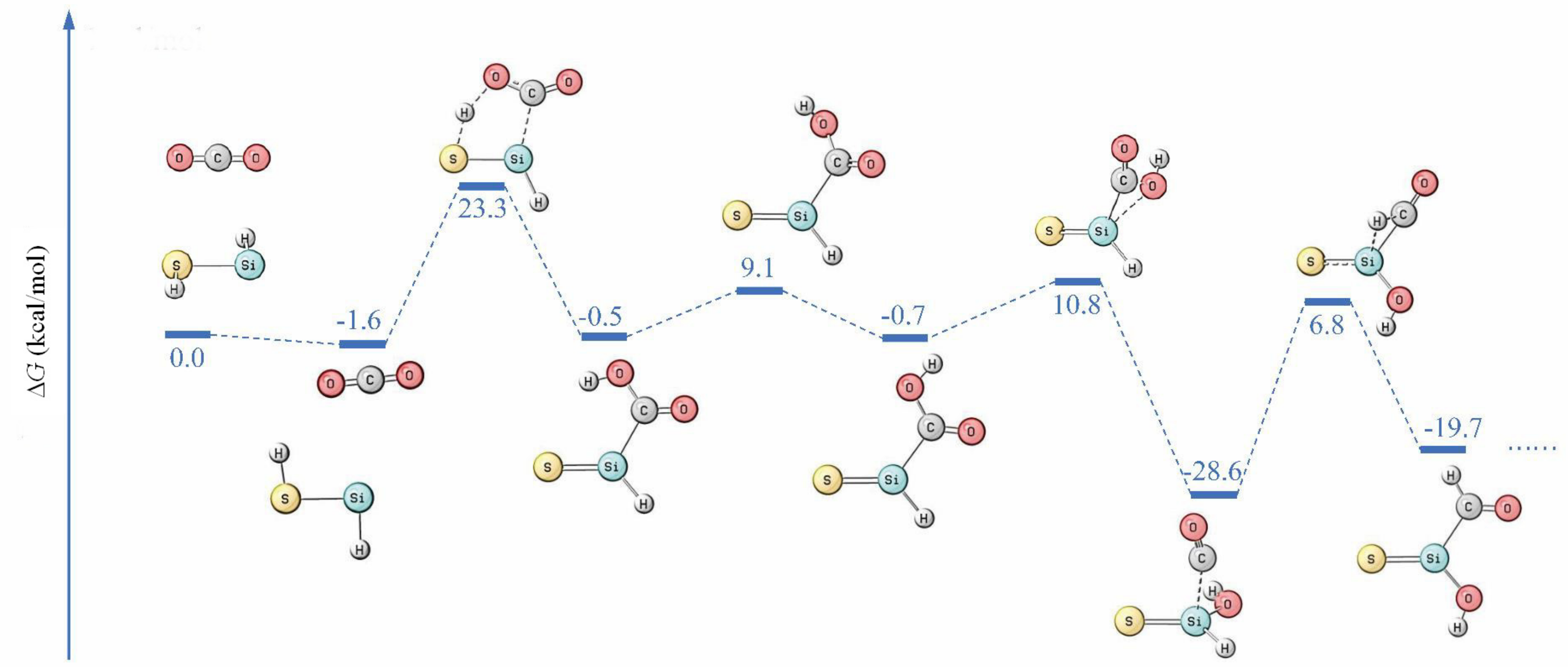
ΔG (kcal/mol)
0.0
-1.6
23.3
-0.5
9.1
-0.7
10.8
-28.6
6.8
-19.7
......

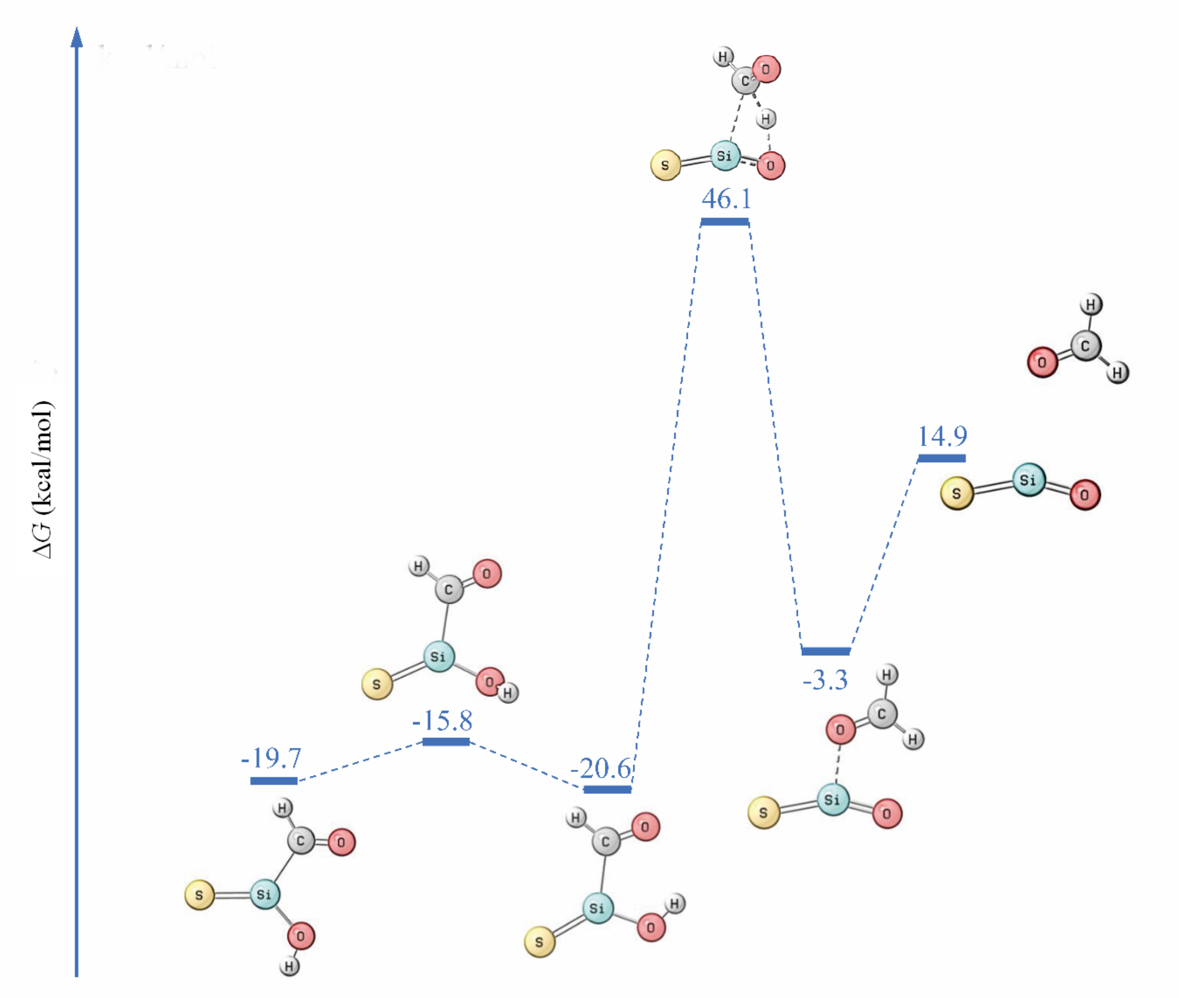
ΔG (kcal/mol)
46.1
14.9
-3.3
-19.7
-15.8
-20.6

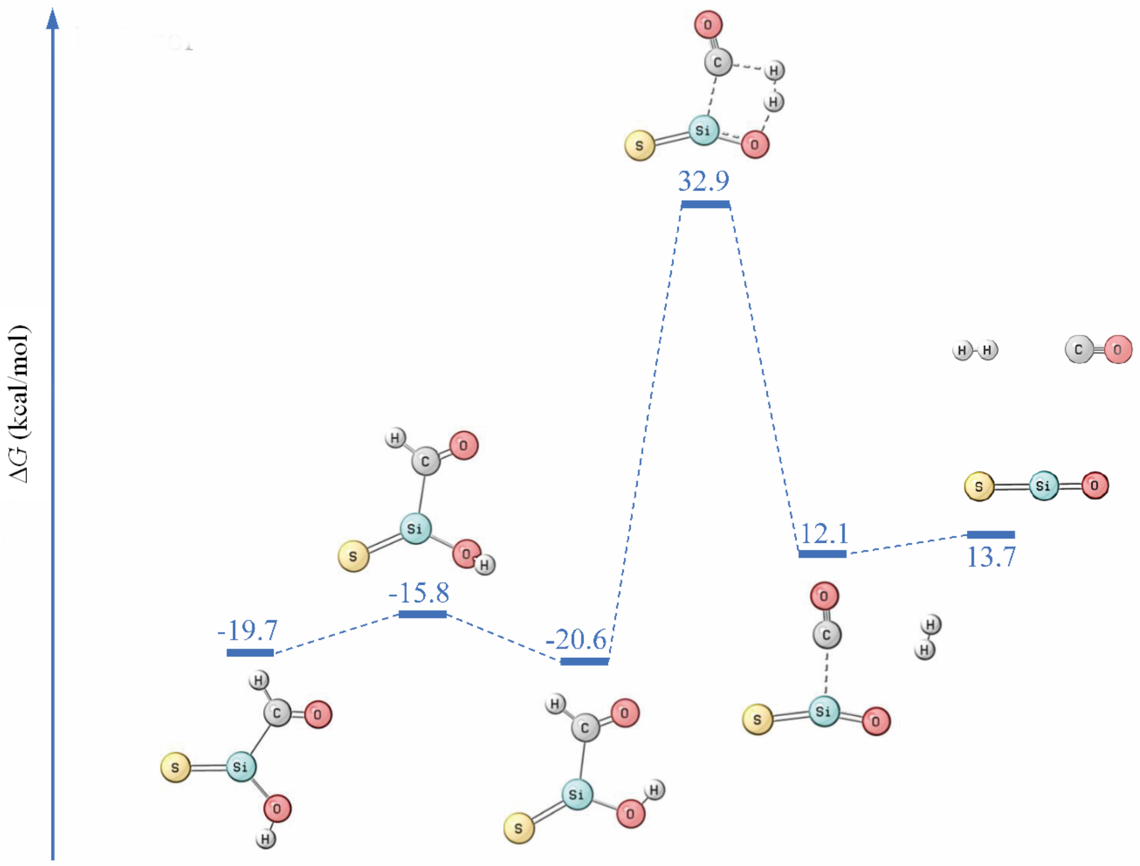
ΔG (kcal/mol)
32.9
12.1
13.7
-19.7
-15.8
-20.6

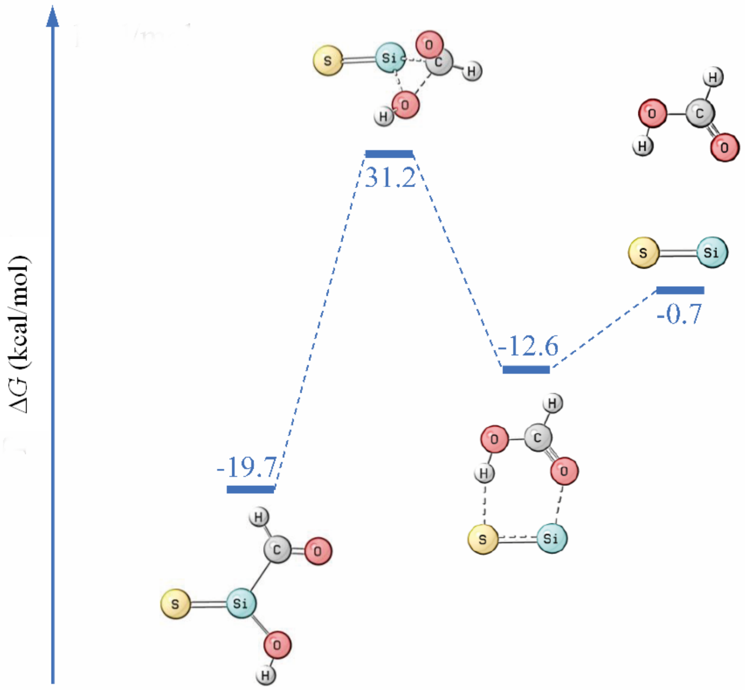
ΔG (kcal/mol)
31.2
-19.7
-12.6
-0.7
S
Si
O
C
H

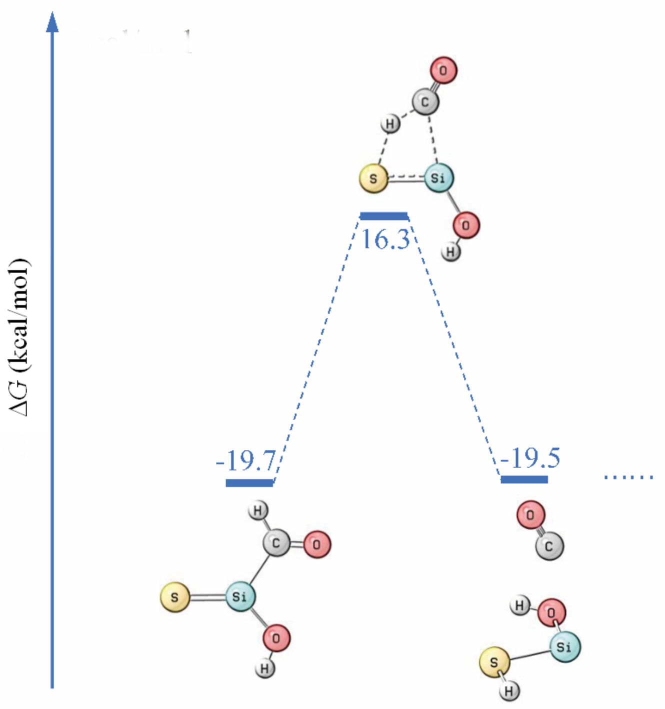
ΔG (kcal/mol)
16.3
-19.7
-19.5
......

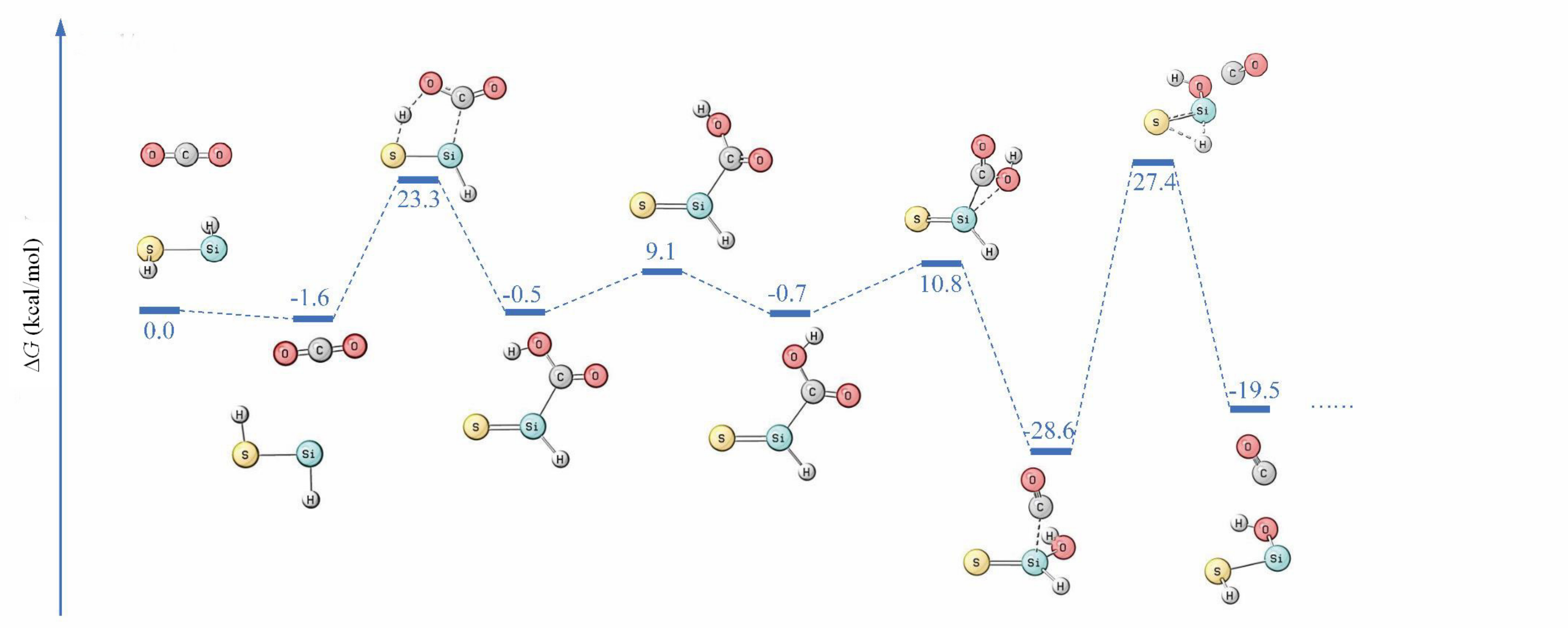
ΔG (kcal/mol)
0.0
-1.6
23.3
-0.5
9.1
-0.7
10.8
-28.6
27.4
-19.5

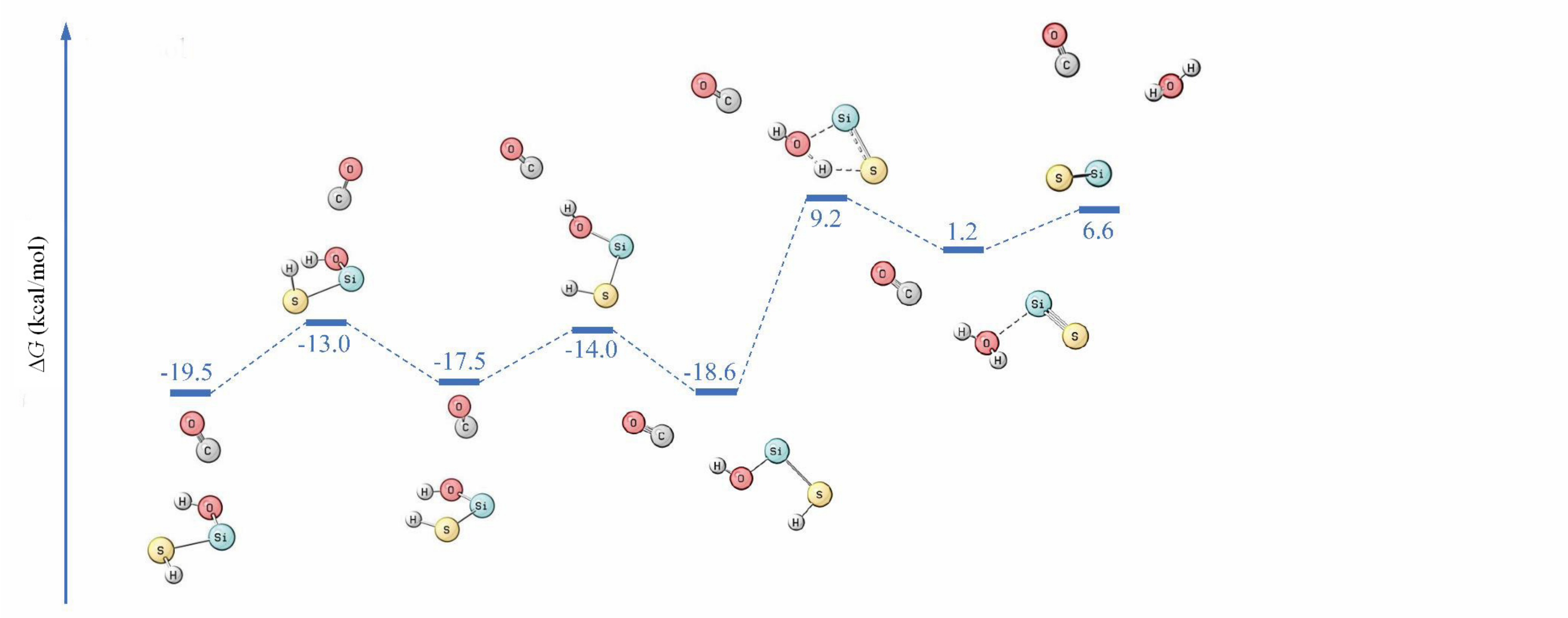
ΔG (kcal/mol)
-19.5
-13.0
-17.5
-14.0
-18.6
9.2
1.2
6.6

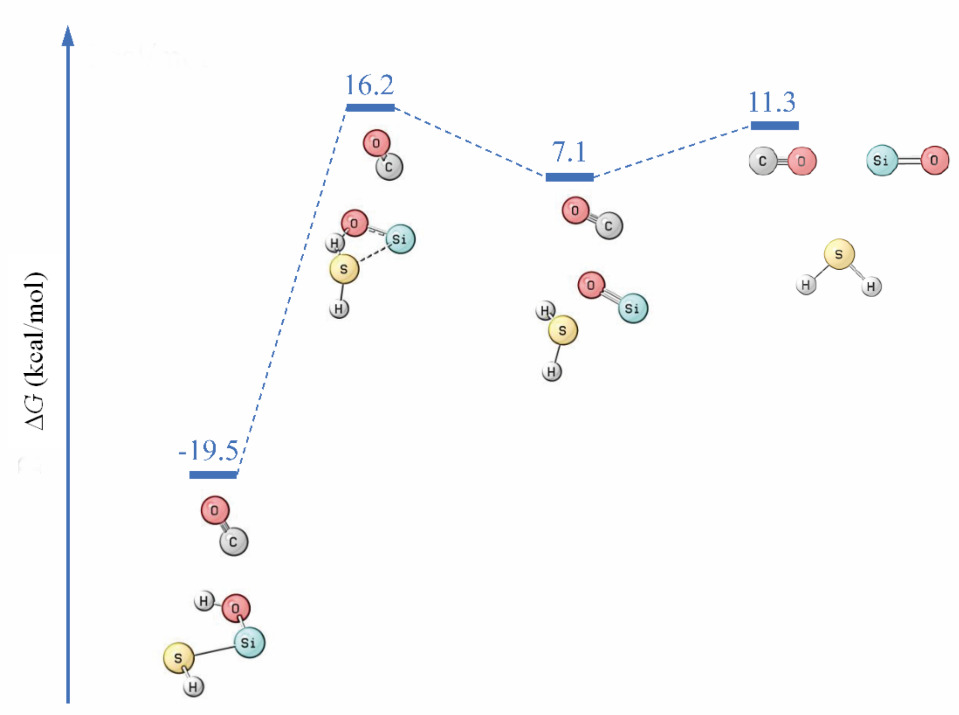

ΔG (kcal/mol)
-19.5
16.2
7.1
11.3

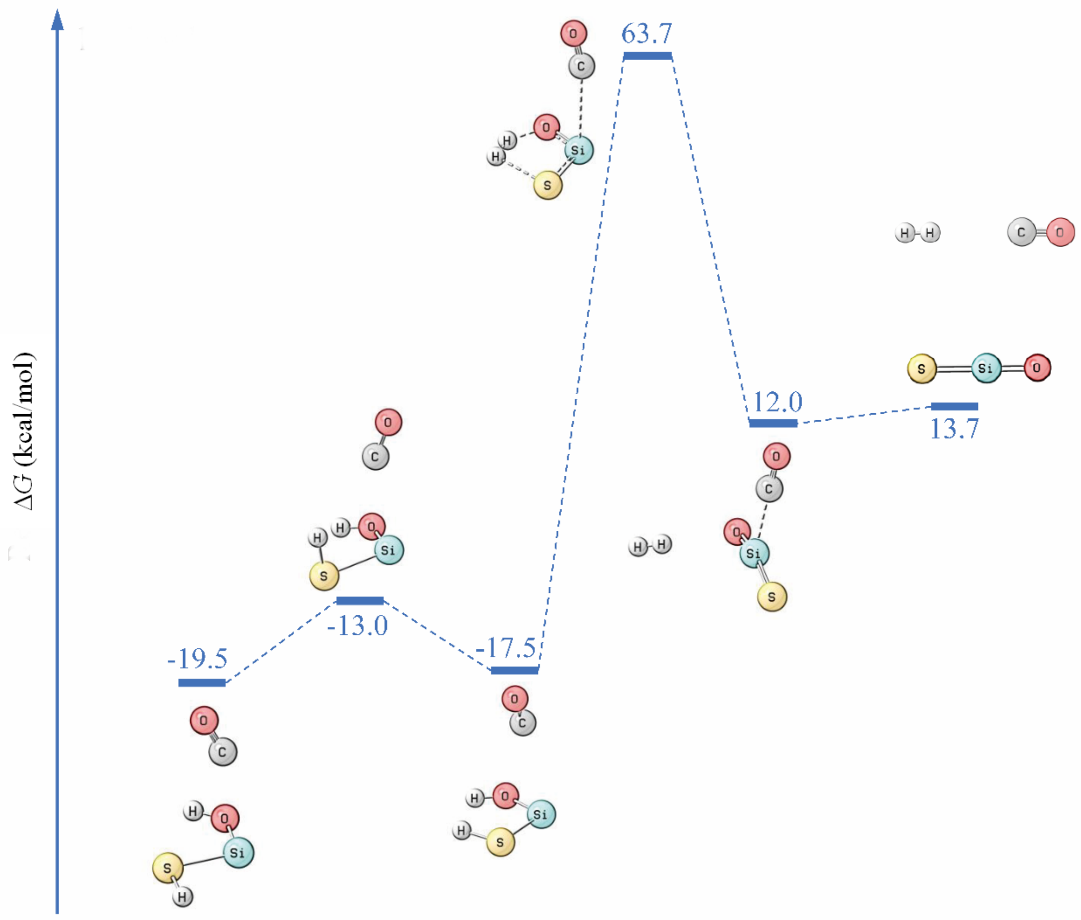

ΔG (kcal/mol)
63.7
12.0
13.7
-19.5
-13.0
-17.5

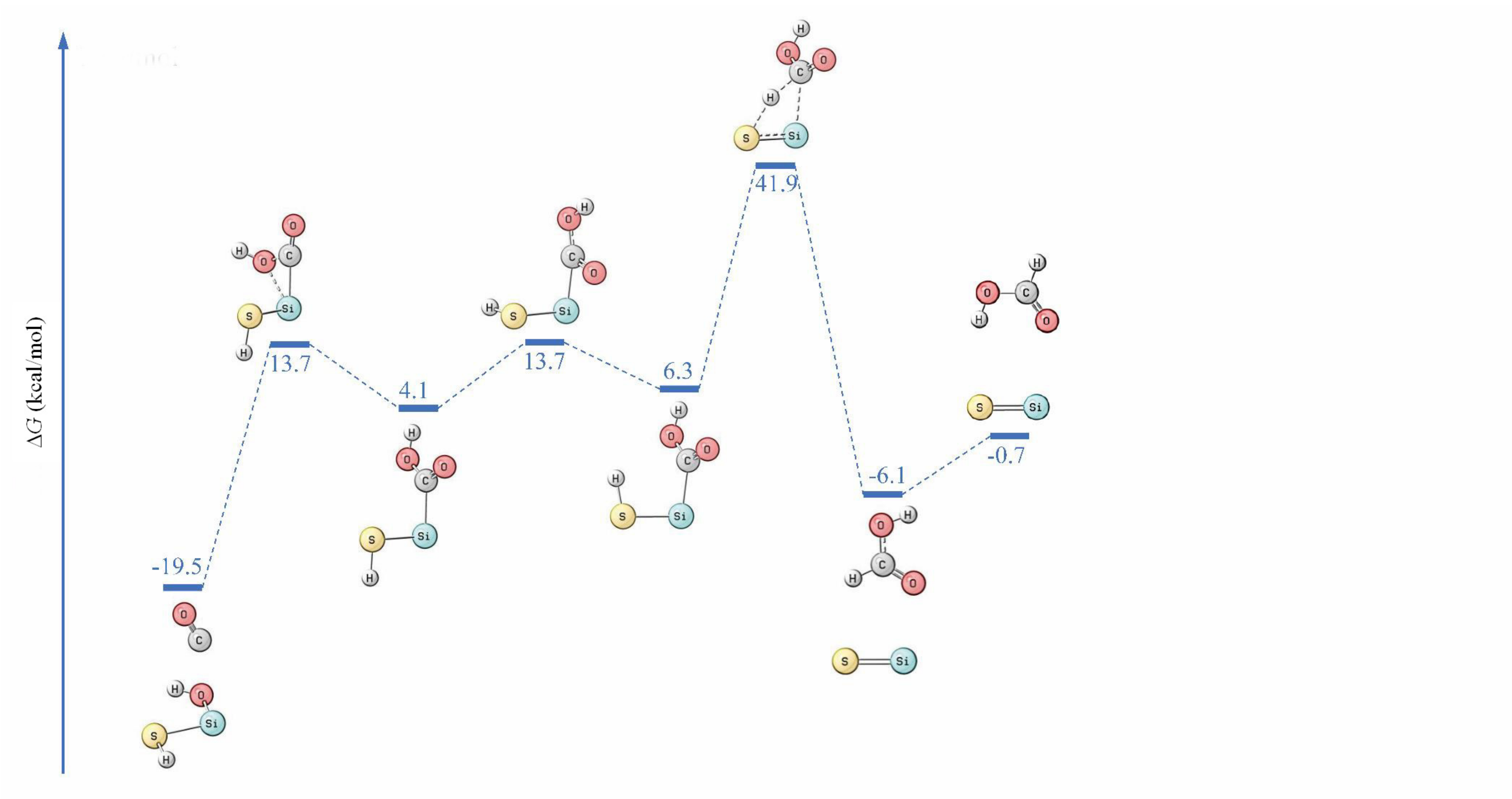
ΔG (kcal/mol)
-19.5
13.7
4.1
13.7
6.3
41.9
-6.1
-0.7

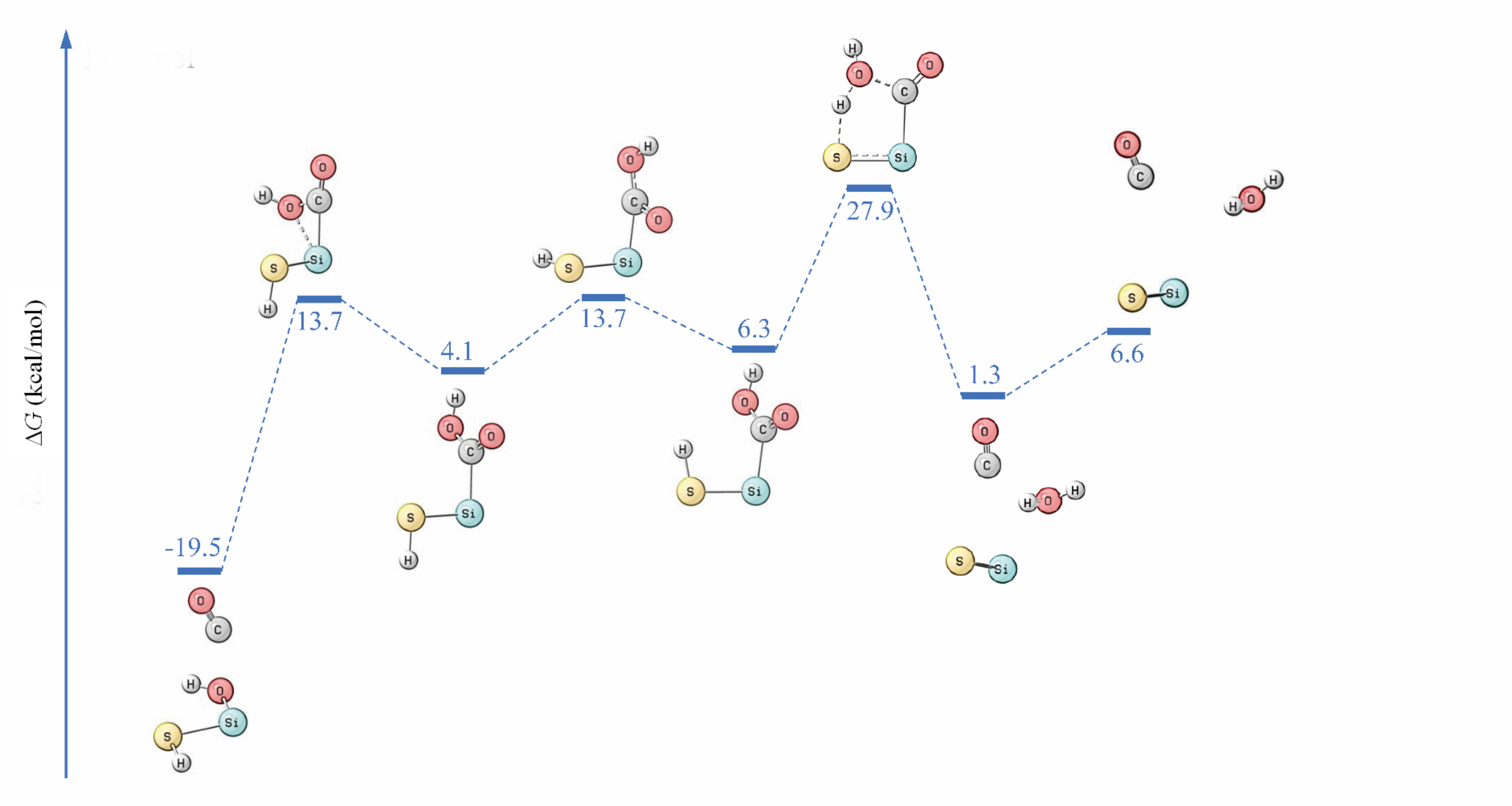
ΔG (kcal/mol)
-19.5
13.7
4.1
13.7
6.3
27.9
1.3
6.6
O
C
H
O
S
Si
H

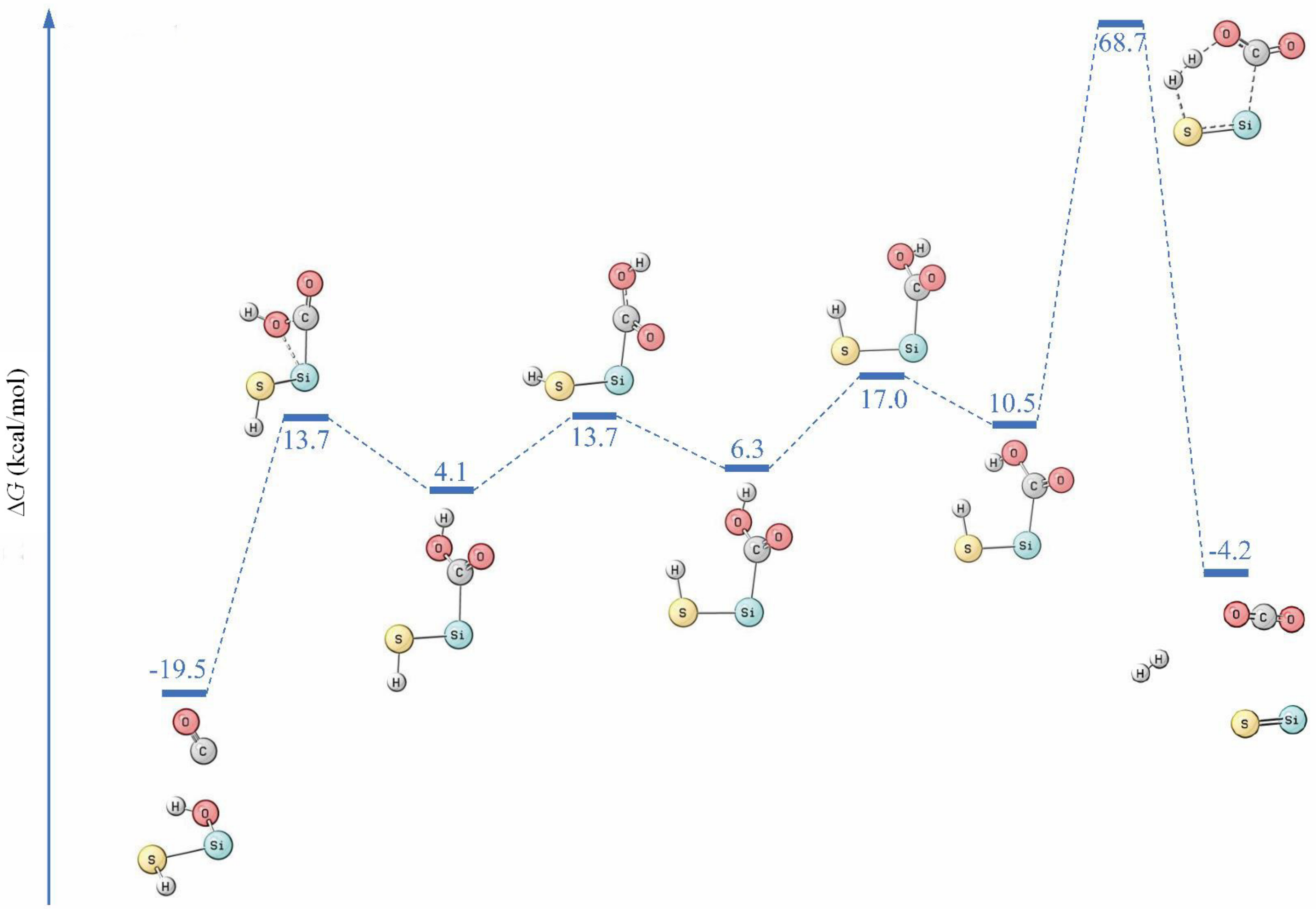

Figure S15. O-end activation pathways for the reaction of HSiSH + CO_2.

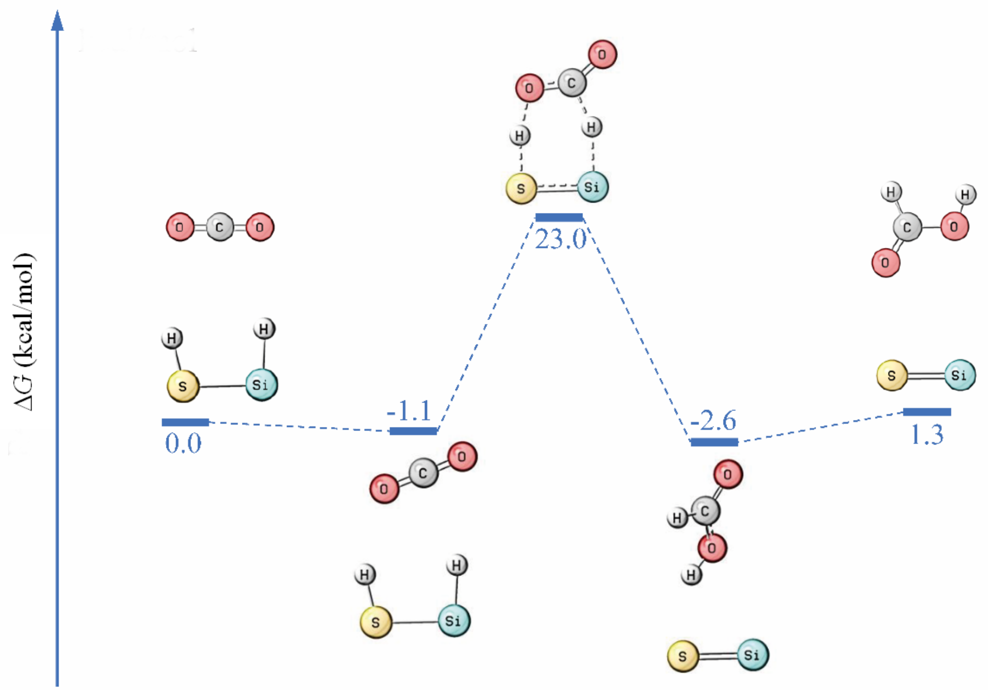

Figure S16. Double-end activation pathway for the reaction of HSiSH + CO_2.
